# Endothelial dysfunction in neuroprogressive disorders—causes and suggested treatments

**DOI:** 10.1186/s12916-020-01749-w

**Published:** 2020-10-19

**Authors:** Gerwyn Morris, Basant K. Puri, Lisa Olive, Andre Carvalho, Michael Berk, Ken Walder, Lise Tuset Gustad, Michael Maes

**Affiliations:** 1 IMPACT – the Institute for Mental and Physical Health and Clinical Translation, School of Medicine, Barwon Health, Deakin University, Geelong, Australia; 2 C.A.R, Cambridge, UK; 3 School of Psychology, Faculty of Health, Deakin University, Geelong, Australia; 4 Department of Psychiatry, University of Toronto, Toronto, ON Canada; 5 Centre for Addiction and Mental Health (CAMH), Toronto, ON Canada; 6 Orygen, The National Centre of Excellence in Youth Mental Health, the Department of Psychiatry and the Florey Institute for Neuroscience and Mental Health, University of Melbourne, Parkville, VIC Australia; 7 Department of Circulation and medical imaging, Norwegian University of Technology and Science (NTNU), Trondheim, Norway; 8 Nord-Trøndelag Hospital Trust, Levanger Hospital, Levanger, Norway; 9 Department of Psychiatry, King Chulalongkorn University Hospital, Bangkok, Thailand; 10 Department of Psychiatry, Medical University of Plovdiv, Plovdiv, Bulgaria

## Abstract

**Background:**

Potential routes whereby systemic inflammation, oxidative stress and mitochondrial dysfunction may drive the development of endothelial dysfunction and atherosclerosis, even in an environment of low cholesterol, are examined.

**Main text:**

Key molecular players involved in the regulation of endothelial cell function are described, including PECAM-1, VE-cadherin, VEGFRs, SFK, Rho GEF TRIO, RAC-1, ITAM, SHP-2, MAPK/ERK, STAT-3, NF-κB, PI3K/AKT, eNOS, nitric oxide, miRNAs, KLF-4 and KLF-2. The key roles of platelet activation, xanthene oxidase and myeloperoxidase in the genesis of endothelial cell dysfunction and activation are detailed. The following roles of circulating reactive oxygen species (ROS), reactive nitrogen species and pro-inflammatory cytokines in the development of endothelial cell dysfunction are then described: paracrine signalling by circulating hydrogen peroxide, inhibition of eNOS and increased levels of mitochondrial ROS, including compromised mitochondrial dynamics, loss of calcium ion homeostasis and inactivation of SIRT-1-mediated signalling pathways. Next, loss of cellular redox homeostasis is considered, including further aspects of the roles of hydrogen peroxide signalling, the pathological consequences of elevated NF-κB, compromised S-nitrosylation and the development of hypernitrosylation and increased transcription of atherogenic miRNAs. These molecular aspects are then applied to neuroprogressive disorders by considering the following potential generators of endothelial dysfunction and activation in major depressive disorder, bipolar disorder and schizophrenia: NF-κB; platelet activation; atherogenic miRs; myeloperoxidase; xanthene oxidase and uric acid; and inflammation, oxidative stress, nitrosative stress and mitochondrial dysfunction.

**Conclusions:**

Finally, on the basis of the above molecular mechanisms, details are given of potential treatment options for mitigating endothelial cell dysfunction and activation in neuroprogressive disorders.

## Background

Recent large meta-analyses of prospective studies have shown that individuals diagnosed with major depressive disorder (MDD) have a significantly increased risk for the development of cardiovascular diseases (CVDs) even when the data are adjusted for confounding variables [1–3]. The evidence suggests that MDD patients experience a 30% increase in CVDs and an approximately 36% increase in mortality due to CVDs compared to age- and sex-matched population norms [2, 3]. The situation in bipolar disorder (BPD) is similar with meta-analyses of prospective studies revealing that the incidence of death due to cardiovascular disease (CVD) is approximately double when compared to the general population [3–5]. The risk of developing CHD may be even greater [5–7]. The importance of CVD as a source of morbidity and mortality in BPD is thrown into stark relief by the presence of data suggesting that this condition may be responsible for up to 40% of deaths in this group of patients [4]. Perhaps unsurprisingly, the weight of evidence also suggests a significantly increased risk of developing CVD in patients afforded a diagnosis of schizophrenia (SZ) [3, 8, 9].

However, authors investigating lipid profiles in patients with neuroprogressive disorders have reported somewhat counterintuitive results with low total and low-density lipoprotein (LDL) cholesterol being the predominant observations. For example, low total cholesterol (TC) and LDL cholesterol (LDLC) have been reported by several prospective studies and meta-analyses investigating lipid profiles in patients afforded a diagnosis of MDD [10–14]. Interestingly, TC and LDL levels may be normalised in responders to electroconvulsive therapy (ECT) [10, 14], reviewed in [15]. Low high-density lipoprotein (HDL) is another common finding in these individuals, which is somewhat more in line with a lipid profile expected in patients at increased risk of developing CVD [13, 14, 16, 17]. Total and LDL cholesterol also appear to be lower in treatment-naïve patients with BPD compared with age- and sex-matched controls [18]. There is also an accumulating body of evidence to suggest that TC levels are lower in patients enduring acute mania compared with levels seen in patients in the depressive phase of their illness [19–21]. In addition, two large meta-analyses have shown decreased levels of TC and LDL in patients with first-episode SZ [22, 23].

This pattern of reduced levels of total and LDL cholesterol is often seen in other illnesses characterised by increased cardiovascular risk and is often described as the lipid paradox [24, 25]. The weight of evidence suggests that the cause of this phenomenon is, at least in part, elevated levels of systemic inflammation [26–30].

This may well be the case in patients with neuroprogressive disorders, as chronic peripheral inflammation, as evidenced by elevated tumour necrosis factor-alpha (TNF-α) and other pro-inflammatory cytokines (PICs), plays a major role in the pathophysiology of SZ [31–33], BPD [34–37] and MDD [38–41].

Importantly, peripheral inflammation also plays a major role in the development of atherosclerosis and CVD [42–44] independently of cholesterol or LDL levels [45–47]. This point is further emphasised by data suggesting that the reduction of systemic inflammation leads to a reduction of cardiovascular events while controlling for levels of total and LDL cholesterol [45–47].

Hence, the presence of systemic inflammation in patients with neuroprogressive disorders may explain increased cardiovascular risk in these individuals even in the context of low TC and HDL. It is also noteworthy that systemic oxidative stress [48–50] and mitochondrial dysfunction [51–53] are also acknowledged players in the pathogenesis of atherosclerosis. This is of interest as oxidative stress [54–58] and mitochondrial dysfunction are involved in the pathophysiology of all the aforementioned neuroprogressive illnesses [58–61].

Mitochondrial dysfunction [62–64], oxidative stress [65, 66] and inflammation [67, 68] are also causatively associated with the development of endothelial dysfunction, activation and senescence. These are relevant observations as endothelial dysfunction [69, 70] and endothelial senescence [71–73] are among the earliest observed abnormalities in the development of atherosclerosis and play an indispensable role in the development of fibrous lesions, consisting of a lipid-rich necrotic core and a cap composed of migratory smooth muscle cells, in large arteries characteristic of the disease. Unsurprisingly, endothelial dysfunction plays an important role in the development of CVD associated with increased risk in apparently disease-free patients with normal Framingham scorers [74–77]. Furthermore, several prospective studies and meta-analyses have demonstrated the presence of endothelial dysfunction in all phases of BPD [78], reviewed in [79]. Similar findings have been reported by researchers investigating the presence of endothelial dysfunction in SZ [80, 81] and MDD [6, 82].

Given the above, it seems reasonable to suggest that the endothelial dysfunction secondary to inflammation, oxidative stress and mitochondrial dysfunction seen in neuroprogressive disorders may be a major factor explaining increased cardiovascular risk in these patients. We have recently proposed that high levels of inflammation, oxidative stress and mitochondrial dysfunction involved in the pathophysiology of MDD, BPD and SZ could potentially explain high levels of obesity, insulin resistance, metabolic syndrome, type 2 diabetes mellitus (T2D) and hypertension seen in patients with these illnesses [56].

This paper examines potential routes whereby systemic inflammation, oxidative stress and mitochondrial dysfunction may drive the development of endothelial dysfunction and atherosclerosis, even in an environment of low cholesterol. In order to do so, we will examine the processes involved in the development of endothelial dysfunction and atherosclerosis in the absence of systemically elevated levels of inflammation, oxidative stress and mitochondrial dysfunction. We will then examine how this triad of abnormalities may mimic such processes. In particular, we will examine how circulating levels of PICs and reactive oxygen species (ROS) may induce inflammation, oxidative stress and mitochondrial dysfunction within endothelial cells (ECs) either directly or indirectly via inducing high levels of platelets, myeloperoxidase and xanthene oxidase activity, which are all independently associated with increased cardiovascular risk [45, 83–85]. We begin with the mechanisms which maintain an anti-inflammatory environment in ECs in physiological conditions and also drive the induction of a pro-inflammatory environment as a prelude to the development of EC dysfunction.

## The development of atherosclerosis

The endothelium plays many vital physiological roles in addition to the delivery of blood which are broadly connected with the maintenance of homeostasis. Metabolically active ECs regulate vasomotor tone, leucocyte trafficking and egress, platelet activity, angiogenesis and multiple aspects of innate and humoral immunity—reviewed in [86]. In physiological conditions of normal blood flow, high shear stress maintains an anti-inflammatory signalling cascade mediated by elevated levels of Krüppel-like factor 2 (KLF2) and via a 5′ AMP-activated protein kinase (AMPK)-dependent mechanism [87–89]. This constitutive activation of KLF2 also plays a major role in maintaining endothelial barrier integrity and EC anti-oxidant systems via the upregulation of nuclear factor erythroid 2-related factor 2 (Nrf2) and endothelial nitric oxide synthase (eNOS) activity, coupled with an increase in occludin synthesis [89–91]. High shear stress also exerts other important and beneficial effects on EC function and metabolism via increased production of nitric oxide, suppression of mitochondrial ROS production and regulation of glycolysis [92].

However, in atheroprone areas of arterial branches and bends, denuded levels of glycolax [93], decreased activity of manganese superoxide dismutase (MnSOD) [94] and low or oscillatory blood flow induce a chronic inflammatory state in resident ECs via the initial upregulation of JNK, p38 MAPK, RelA, IKK, p65 and ultimately the persistent activation of NF-κB [95–99], reviewed in [100].

Disturbed or oscillatory flow patterns can also result in the development of inflammatory status within ECs by inducing the development of endoplasmic reticulum (ER) stress and activation of the unfolded protein response (UPR) via the activation of the PI3k Akt signalling pathway [101, 102]. Activation of the UPR can exacerbate the inflammatory environment within ECs by stimulating further increases in levels of NF-κB activation [103, 104]. Disturbed blood flow can also induce EC senescence via the activation of the p53/p21 pathways leading to a senescence-induced secretory phenotype characterised by low levels of NO, increased activity of the transcription factors pCREB and Elk and elevated levels of p38 MAPK, PICs and ROS [105, 106]. Senescence and UPR activation may increase EC activation and dysfunction as a result of increased activity of NF-κB, p38 MAPK, pCREB and Elk, which lead to increased levels of PICs and ROS production coupled with reduced levels of NO due to inhibition of eNOS [105, 106]. It is important to stress that EC senescence and upregulation of the UPR are considered to be major independent risk factors for the development of atherosclerosis because of their role in exacerbating EC activation and dysfunction, as discussed above [71, 107, 108].

EC activation results in increased permeability to circulating lipoproteins coupled with a significant accumulation of extracellular matrix proteins, which facilitates the sequestration of the highly atherogenic oxidised apolipoprotein B (apoB), the main constituent of LDL in the intima region of the arterial wall [70, 109], reviewed in [69]. The activation of the endothelium also promotes the recruitment of circulating monocytes and their ultimate recruitment into the arterial intima via the upregulation of EC chemokines, most notably CCL5, CXCL1, the cytokines MCP-1 and IL-8 and the surface adherence proteins VCAM-1, ICAM-1 and P-selectin EC and several glycosaminoglycans [110–112]. The internalisation of oxidised LDL (oxLDL) by macrophage scavenger receptors and subsequent foam cell formation is a vital step in the development of atherosclerosis, and this process has been the subject of intense research and discussed in depth in several excellent reviews [113–115]. The argument examined here is that abnormally high levels of EC dysfunction, senescence and activation enable excessive levels of LDL and macrophage recruitment into the intima, thereby fostering the development of atherosclerosis in a low cholesterol environment. In the case of neuroprogressive illnesses, the proposed sources of such endothelial dysfunction and activation are excessive levels of PICs, ROS, reactive nitrogen species (RNS) and mitochondrial dysfunction, which is discussed and detailed below. However, while the inflammatory consequences of low or oscillatory blood flow patterns have been discussed above, no information has been provided which explains the mechanisms involved and how they might be compromised in an environment of chronic inflammation and oxidative stress. Hence, this area will be addressed in the next section of the paper with a focus on three main players, namely the mechanosensitive proteins platelet endothelial cell adhesion molecule-1 (PECAM-1) and VE-cadherin and a family of flow-sensitive microRNAs (miRNAs).

## Molecular players involved in regulating EC function

**Fig. 1:**
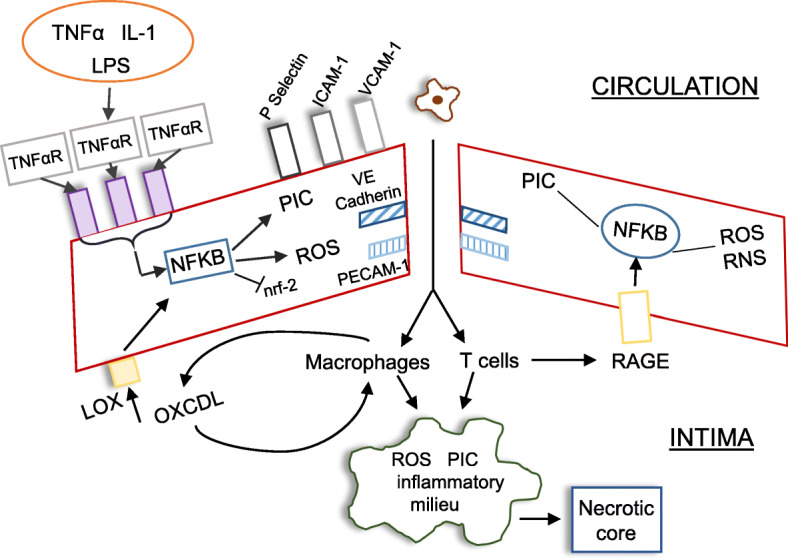
The antagonistic relationship between NF-κB and KLF in endothelial dysfunction. In physiological conditions, the vascular endothelial is largely maintained in quiescent and impermeable state by the constitutive activity of KLFs and the mechanosensory proteins VE-cadherin and PECAM-1. The upregulation of the former results in the upregulation of nrf-2 and eNOS together with concomitant inhibition of mtROS production while inhibiting the transcriptional activity of NF-κB, while the activity of VE-cadherin and PECAM-1 physically increases the contact between two adjacent ECs. In an environment of chronic inflammation, however, the activation of NF-κB, induced by inflammatory mediators such as TNF-α or LPS, directly or indirectly inhibits the activity of KLF, PECAM-1 and VE-cadherin leading to a loss of tight junction integrity and the development of EC activation. The latter is associated with upregulation of surface chemokine receptors and adhesion factors resulting in the recruitment of LDL, activated monocytes and T cells into the vascular intima. The resultant oxidation of LDL and internalisation by monocyte-derived macrophages leads to foam cell formation and the development of a plaque with a highly necrotic core. Oxidised LDL can provoke increased activation and dysfunction of ECs via engagement with LOX-1 receptors allowing for the development of self-amplifying vascular and systemic inflammation

Unsurprisingly, there has been extensive research aimed at delineating the mechanisms which enable changes in blood flow dynamics to produce beneficial or pathological consequences within ECs, and several mechanosensory sensors and transducers have been proposed, reviewed in [116]. The weight of evidence thus far suggests that the process is initiated and regulated by a “mechanosensory” complex of proteins located at EC junctions composed of PECAM-1 indirectly connected to the cytoskeleton via vimentin, VE-cadherin and the functionally pleiotropic vascular endothelial growth factor receptors VEGFRs 1 and 2 [117, 118]. Fluid stress modulates tension between PECAM-1 and VE-cadherin, which in physiological conditions results in increased tension across PECAM-1 and reduced tension across VE-cadherin [117, 119, 120]. A diagrammatic representation of this mechanosensory complex and its mode of action is provided in Fig. 1.

Briefly, PECAM-1 transduced forces activate as yet unidentified src family kinases (SFKs) leading to the transactivation of VEGFRs; VE-cadherin, on the other hand, serves as an adaptor interacting with VEGFRs, inducing their activation in flow [121]. The flow sensing capacity of VE-cadherin is dependent on the SFK-mediated phosphorylation status of Tyr 658, which is at maximum during shear stress [121, 122]. This is important as phosphorylation-dependent VE-cadherin signalling via the scaffolding protein Rho GEF TRIO and upregulation of RAC-1 enable and regulate the actin cytoskeleton reorganisation which determines the EC responses to different flows [123, 124]. The level of VE-cadherin phosphorylation also plays a large role in maintaining tight junction integrity [125] and forms part of the EC defences against inflammatory agents and leucocyte binding [124].

Levels of phosphorylation also determine the activity of PECAM-1. In this instance, phosphorylation levels of the so-called tyrosine-based inhibition sequence (ITAM) largely determine its signalling capabilities, which regulate actin cytoskeleton rearrangement, tight junction integrity and intracellular signalling pathways, reviewed in [126]. Briefly, in physiological conditions, phosphorylation of ITAM leads to the recruitment of SHP-2 and subsequent phosphorylation and activation of MAPK/ERK pathways and STAT-3, ultimately leading to the inhibition of NF-κB activity [127–129]. Conversely, in conditions of low shear stress, reduced levels of ITAM phosphorylation relieve the inhibition of NF-κB nuclear translocation, leading to a cascade of inflammatory signalling thought to be mediated via the activation of the PI3K/AKT pathway [130–132].

It should also be noted that PECAM-1 is associated with eNOS at the plasma membrane and this association allows the regulation of this protein’s activity and that of VE-cadherin by changes in levels of NO [130, 132, 133]. Unsurprisingly, dysfunction of PECAM-1 and/or VE-cadherin is associated with the development of atherosclerosis and CVDs [130, 132, 133]. Importantly, such dysfunction may be induced by a range of atherogenic pro-inflammatory miRNAs, reviewed in [134]. The role of miRNAs in the regulation of EC function and their potential role in the development of EC pathology are discussed below.

Flow-sensitive miRNAs, often described as “mechanomiRs”, modulate the expression of EC genes and hence play indispensable roles in the regulation of EC homeostasis and the development of atherosclerosis, and can regulate endothelial dysfunction and atherosclerosis [135, 136]. miRNAs such as miR-200, 92a, 143/145, 134 and 155 have been identified as major players in the development of EC dysfunction [135, 136], and their transcription and translation are increased as a result of disturbed flow [137–139]. Readers interested in a detailed consideration of this topic are referred to excellent treatments of the subject by [139, 140].

**Fig. 2:**
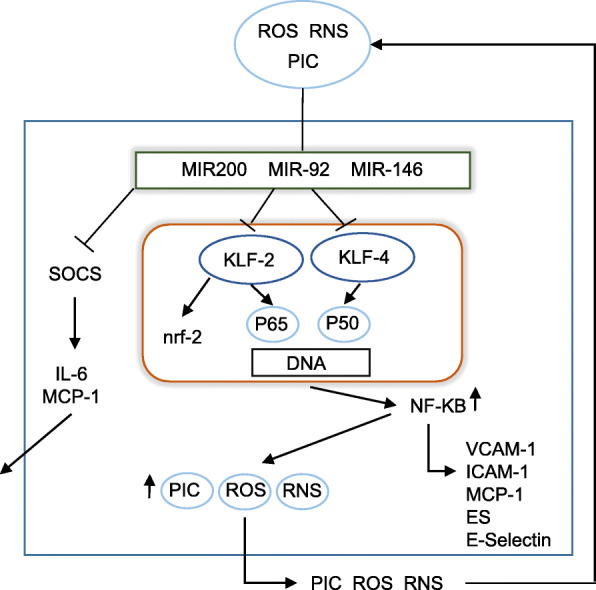
The pathogenic effects of upregulated atherogenic mechanosensory miRNAs. In conditions of high sheer stress, mechanosensitive miRNAs play a major role in maintaining the function and integrity of the vascular epithelium. However, in an environment of chronic inflammation and oxidative stress, the consequent upregulation of atherogenic miRNAs such as miRNA-92 induces EC dysfunction and activation by inhibiting the activity of KLFs and, to a lesser extent, SOCS-1. The resultant upregulation of NF-κB and SOCS-1 increases the internal production of PICs, MCP-1 and IL-6 and stimulates the increased expression of adhesion factors and chemokines on the EC surface. The resultant release of cytokines into the environment increases the inflammatory milieu and may establish a self-amplifying environment of inflammation and oxidative stress with the ECs and beyond

Upregulation of miR-92 activity would appear to play an indispensable role in the development of EC dysfunction as evidence from animal studies suggests that the development of atherosclerosis may be arrested or even reversed by inhibition of this molecule [141, 142]. Mechanistically, this miR exerts pathology mainly by inducing decreases in the activity of KLF-4 and KLF-2 leading to the upregulation of NF-κB [143–146]. The antagonistic relationship between these KLFs and NF-κB is due to the fact that they compete for access to p300/CBP which acts as an essential coactivator for both transcription factors; hence, a decrease in KLF-2 and KLF-4 leads to upregulated NF-κB activity and vice versa [147, 148]. Elevated miR-92a activity also results in increased phosphorylation of the NF-κB subunit p65 via a mechanism which remains to be delineated [146]. The net effect of upregulated miR-92a activity is increased expression of inflammatory and endothelial adhesion markers such as PICs, E-selectin, CCL2 and VCAM-1, and decreased activity of eNOS, which in their entirety increase atherosusceptibility [143, 149]. The weight of evidence also suggests that other miRNAs involved in inducing EC dysfunction, such as miRs 155, 200, 34 and 146, also inhibit KLF-4 and KLF-2, leading to the upregulation of NF-κB [137, 150–154]. The actions of KLFs in regulating the development of EC activation are diagrammatically represented in Fig. 2.

The paper now moves on to discuss how the various elements driving the pathophysiology of neuroprogressive illnesses might conspire to produce very high levels of endothelial dysfunction and increased levels of atherosclerosis. The discussion commences with a consideration of the effects of platelet activation (PA), increased xanthene oxidase (XO) activity and elevated levels of myeloperoxidase (MPO), which are all characteristic abnormalities found in an environment of chronic inflammation and oxidative stress.

## The roles of platelet activation, xanthene oxidase and myeloperoxidase in the genesis of EC dysfunction and activation

### Role of activated platelets

Platelets may be activated by high levels of circulating PICs and ROS [155–157], reviewed in [158]. This is of importance from a pathophysiological perspective as PA is a major source of systemic inflammation and oxidative stress [159, 160]. Activated platelets secrete high levels of PICs and ROS and a plethora of chemokines, TNF superfamily members and adhesion factors which make an independent and collective contribution to initiating or exacerbating levels of EC activation and dysfunction [161–163], reviewed in [164]. For example, the TNF superfamily member LIGHT enhances platelet EC adhesion, EC dysfunction and EC activation by stimulating elevated activity of NF-κB via a pathway dependent on MAPK [165–167]. It should also be noted that platelet-mediated release of LIGHT may also be a source of increased systemic inflammation [165]. The weight of evidence suggests that platelet-secreted CD40L, another TNF superfamily member, also plays a major role in initiating or exacerbating EC dysfunction and activation via several routes [168]. Such routes include increased activity and transcytosis of metalloproteins, reduction of NO production and elevated transcription of NF-κB [168–170]. Platelet-derived CD40L also appears to make an independent contribution to the initiation and/or exacerbation of systemic inflammation and oxidative stress [169].

There are some 50 members of the chemokine family, and many are secreted by activated platelets; clearly, a detailed consideration of this area is beyond the scope of this paper. Hence, readers interested in the area are encouraged to consult the work of [171]. However, two platelet-derived chemokines, CCL5, also known as RANTES, and CCL4, also known as platelet factor 4 (PF4), have been the subject of intense research, reviewed in [172, 173], and as their activities are germane to the central theme of this paper, their modes of action will be briefly discussed below.

RANTES promotes leucocyte recruitment to the endothelium in much the same manner as other platelet-derived cytokines. However, this chemokine also promotes leucocyte survival and polarised activation towards a PIC- and ROS-secreting phenotype coupled with increasing adhesion of such leucocytes to ECs [174, 175]. PF4 possesses several unusual properties, in addition to leucocyte recruitment, which encourage the development of endothelial dysfunction and increased systemic inflammation. Such properties include the promotion of monocyte differentiation into macrophages, suppression of macrophage apoptosis, anchoring macrophages to ECs and binding to LDL [176, 177]. The weight of evidence suggests that engagement of PF4 and LDL increases the binding affinity of the latter to LDL receptors on platelets, macrophages and ECs while inhibiting endocytotic “machinery” retaining the lipoprotein at the surface, allowing enhanced exposure to ROS and inflammatory molecules resulting in its increased oxidation [177–179]. Moreover, there is evidence to suggest that the internalisation of PF4-oxLDL complexes by macrophage scavenger receptors increases the efficiency of foam cell formation over tenfold [180]. These data are of interest as they offer another route by which levels of oxLDL and the efficiency of the lipoprotein in inducing foam cell formation may be increased and thereby potentially compensate for relatively low levels of LDL in the circulation. Finally, there are data to suggest that initial activated platelet-mediated oxidation of LDL further enhances PA via a MAPK- and NADPH oxidase 2 (NOX2)-dependent signalling pathway, further increasing systemic levels of ROS, RNS and PICs [177].

### Role of xanthene oxidase

High levels and activity of XO constitute a characteristic feature of many illnesses and conditions, such as T2D and metabolic syndrome, whose pathophysiology is driven at least in part by chronic systemic oxidative stress and inflammation [181, 182]. The weight of evidence also suggests that high levels of circulating XO act as a major driver of endothelial dysfunction and atherosclerosis [183], reviewed in [184]. The pathogenic role of XO is further emphasised by data produced by several meta-analyses and prospective studies demonstrating a significant and large improvement in endothelial function following XO inhibition in patients with CVD [84, 185–187]. A recent meta-analysis of large prospective randomised controlled trials (RCTs) has also reported large reductions in cardiovascular morbidity and mortality achieved by the inhibition of XO by allopurinol [188].

**Fig. 3:**
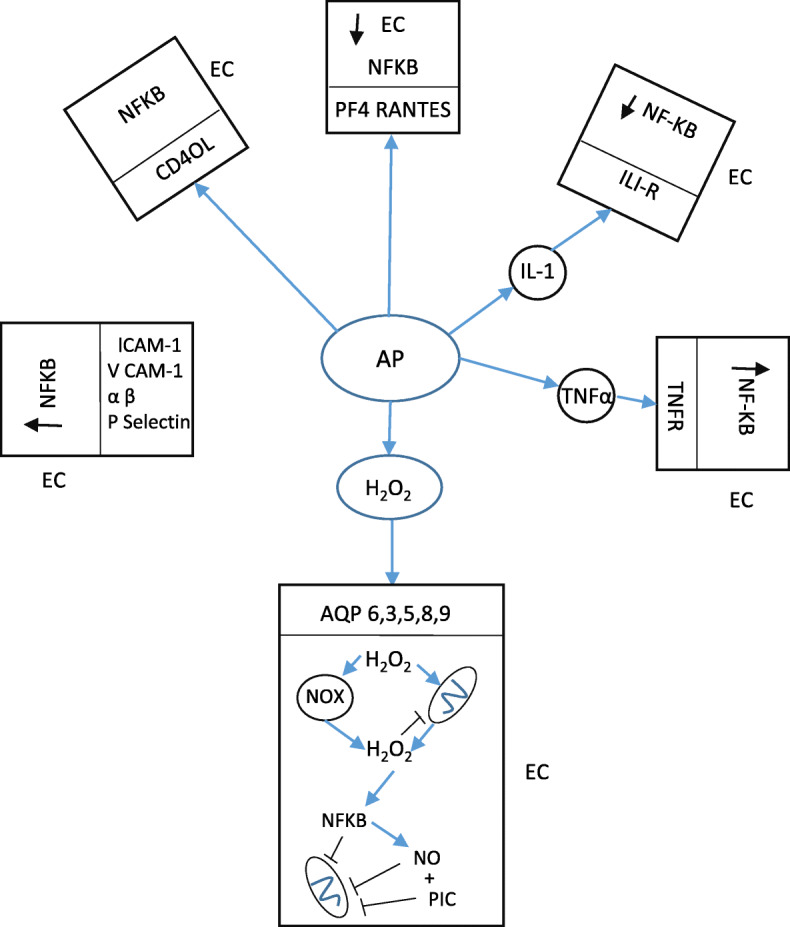
The damaging effects of activated platelets on endothelial cell function and activation. Activated platelets release large quantities of PICs, ROS and chemokines such as CD40, RANTES and PF4. PICS and CD40 can engage their cognate receptors on the surface of ECs activating downstream signalling pathways culminating in the activation of NF-κB. PF4 and RANTES may also engage with the surface of ECs, thereby summoning leucocytes and stimulating their differentiation and activation via a range of mechanisms ultimately also resulting in EC NF-κB activation. In addition, high levels of circulating hydrogen peroxide, produced by the activity of platelets, neutrophils and allopurinol, may directly enter ECs via aquaporin receptors. Such influx results in the activation of hydrogen peroxide production by NOX enzymes and mitochondria ultimately acting as another vehicle driving NF-κB upregulation. The subsequent upregulation of NO, PICs and ROS also compromises mitochondrial ATP production while the NF-κB-mediated downregulation of SIRT-1, PGC-1α and PPAR-γ inhibit mitochondrial biogenesis and disrupt many mechanisms regulating mitochondrial dynamics. The result is self-amplifying inflammation oxidative stress and mitochondrial dysfunction within the EC and potentially an increase in systemic inflammation

One mechanism which appears to be associated with the positive effects of XO is a decrease in systemic and vascular oxidative stress [84, 185–187]. This is unsurprising given the fact that circulating activated XO is a major, if not the predominant, source of hydrogen peroxide and superoxide in patients displaying high levels of systemic inflammation and oxidative stress [183, 189, 190]. The source of increased circulating XO in such conditions is not fully delineated but appears to be associated with increased transcription stimulated by the presence of high levels of TNF-α and other PICs [191, 192]. In contrast, the mechanism explaining ROS production by XO is well documented and occurs as a result of their role in catalysing the oxidation of hypoxanthine [193, 194]. The pathways involved in purine catabolism are illustrated in Fig. 3. The direct effect of XO in inducing EC dysfunction appears to be induced by binding to the EC membranes before being internalised via endocytosis [195–197]. Once internalised, XO acts as a source of increased superoxide and hydrogen peroxide levels contributing to increasing levels of oxidative stress and inflammation [198]. Increased levels of XO activity can also make an indirect contribution to increasing levels of inflammation and oxidative stress by catalysing the production of uric acid (UA).

### Increased levels of uric acid

Impaired EC function is also associated with increased UA levels in the plasma [195, 199]. Moreover, several authors have reported an inverse association between UA levels and EC function [200, 201]. There is also evidence to suggest that increased levels of circulating UA are associated with an increased risk of cardiovascular morbidity and mortality [202–204]. There remains the question as to whether such an association is a consequence of increased XO activity, but nevertheless, the current weight of evidence strongly suggests that elevated UA levels are an independent predictor of CVD [203, 205].

The internalisation of UA into ECs appears to be facilitated by a range of different surface membrane urate transporters such as Glut-9 and URAT-1 [206–208]. Readers interested in the classification and mechanisms enabling the performance of these receptors are invited to consult an elegant and comprehensive review on the subject by [209]. The consequences of UA internalisation include increased levels of PICs, chemokines, EC adhesion molecules and ROS, coupled with elevated activity of NF-κB and reduced production of NO which all contribute to the development of EC dysfunction [208, 210–212].

The mechanisms underpinning this pattern of pathology and the subsequent development of endothelial dysfunction appear to be numerous. For example, UA internalisation may induce ROS-, PIC- and NF-κB-mediated EC dysfunction by the stimulation of HGMB1/Rage signalling [211, 213]. Readers interested in a detailed consideration of the mechanism involved are referred to the work of [214]. There may also be other mechanisms involved as UA may act as an alarmin, and in some circumstances, high levels of this purine lead to activation of the NLRP3 inflammasome [215, 216]. Internalised UA may also increase activity of NF-κB levels, and inflammatory and oxidative and nitrosative stress (I&ONS) within ECs, by inhibiting eNOS via disruption of the association of the enzyme with its primary activator calmodulin [217] and inducing the activation of NOX [218–220]. There is also evidence to suggest that circulating UA can activate PPRs on ECs, which would be another route resulting in the activation of NF-κB [221]. Other mechanisms whereby high levels of circulating UA may induce elevated levels of I&ONS in ECs involve the activation of the (pro)rennin receptor found on the surface of ECs [212, 222] and activation of the vascular renin angiotensin system and/or ERK signalling [223–225].

### Myeloperoxidase

Elevated levels and activity of MPO in the circulation are an accepted marker of systemic oxidative stress and inflammation [226, 227]. This is unsurprising given that elevated MPO levels in the circulation are the result of ROS- and PIC-mediated degranulation of neutrophils, which act as the main reservoir of this enzyme in humans [226–228]. From a pathological perspective, it is important to note that active MPO is a major source of ROS, RNS and reactive radicals responsible for causing severe cellular damage, most pertinently to the protective endothelial glycocalyx layer, tight junction integrity and individual ECs [227, 229].

There is also evidence to suggest that MPO binding to APOB-100 is one of the molecular players responsible for the oxidation of LDL [230]. Furthermore, the MPO-oxLDL complex (MOX-LDL) also has a potent effect on EC and macrophage activation, with the resultant secreting of PICs and ROS which appears to be greater than that achieved by oxLDL alone, thereby making a significant contribution to increasing levels of intracellular and extracellular inflammation and oxidative stress [230–232]. In addition, it would appear that the internalisation of MOX-LDL by macrophage scavenger receptors greatly increases the efficiency of foam cell formation [230, 233]. This is of interest given the relatively low levels of LDL generally present in patients with neuroprogressive disorders as it offers a plausible mechanism which might increase the atherogenicity of the LDL present.

Several research teams have reported an association between, on the one hand, chronically elevated MPO activity and increased EC dysfunction and, on the other hand, increased cardiovascular morbidity and mortality [234, 235]. There is some suggestion that increased levels and activity of MPO may also be a consequence of elevated XO activity [236, 237]. This would be consistent with increased levels of oxidative stress driven by the superoxide, hydrogen peroxide and UA produced by circulating XO, which can induce neutrophil degranulation and MPO release into the circulation [227, 238]. The reduction in MPO levels achieved by XO inhibition in prospective RCTs also hints at the dependence of elevated levels of MPO on increased XO activity [236, 237]. However, it would appear that the adverse effects of MPO are independent of those exerted by XO and UA.

The internalisation of MPO by ECs is achieved via a different mechanism from that of the internalisation of XO and UA. In this instance, the transfer of MPO into ECs is achieved either by the contact of neutrophils and ECs via beta integrins [235] or via the engagement of free MPO with EC surface cytokeratin-1 receptors [238]. This internalisation is thought to contribute to the development and/or exacerbation of EC dysfunction by increasing the catabolism of NO and via the chlorination of arginine thereby inhibiting the activity of eNOS [239–241].

Having reviewed the roles of PA, XO, UA and MPO in the development of EC dysfunction and activation, the next section considers the role of circulating ROS, RNS and PICs in the development of such pathology.

## Roles of circulating ROS, RNS and PICs in the development of EC dysfunction

### Paracrine signalling by circulating hydrogen peroxide

In a state of systemic ONS, circulating ROS directly interact with ECs resulting in increased ROS production within these cells. Mechanistically, this is achieved via the diffusion of hydrogen peroxide into ECs, which is facilitated by the presence of plasma membrane water channels or aquaporins (AQPs), most notably AQP1 and 3, leading to the activation of several NOXs [242–244]. Evidence suggests that in this scenario, activated NOX2 and NOX4 are the most important players being, in the main, generators of superoxide radicals and hydrogen peroxide, respectively [245, 246]. Readers interested in the role of NOXs in the regulation of cellular redox homeostasis and their responses to various stimuli are referred to elegant reviews by [247, 248], and these matters will not be considered further here. From a pathological perspective, however, the activation of NOX 2 and NOX 4 increases ROS production by mitochondria and XO, which act to increase ROS production further by NOXs, forming a self-amplifying positive feedback loop [249, 250]. This level of ROS production could of course be further amplified by internalised MPO, XO or UA originating in the cytoplasm via the mechanisms discussed above. In any event, spirally increasing levels of ROS can induce several dimensions of pathology including inhibited eNOS activity, loss of cellular redox homeostasis and increasing mitochondrial damage contributing to the development of endothelial dysfunction, activation and senescence via several routes which are discussed below.

### Inhibition of eNOS

Increasing levels of hydrogen peroxide may stimulate increased activation of eNOS via a pathway involving the phosphorylation of Akt and AMPK [251, 252]. This is important from a pathological perspective as the increased level of NO produced by the stimulation of this enzyme within the EC may react with superoxide produced by NOXs and XO to form peroxynitrite with devastating consequences as far as the production of NO by eNOS is concerned for reasons explained below [253–255].

In physiological conditions, eNOS exists as a dimer with a reductase domain composed of flavins, a calmodulin binding site and NADPH, together with an oxidase domain composed of a haem active site bound to arginine, oxygen and tetrahydrobiopterin (BH_4_) [256, 257]. This structure allows the transfer of electrons from NADPH to the haem site where the bound oxygen is reduced before being incorporated into arginine to form NO and citrulline [256, 257]. Crucially, BH_4_ is an indispensable cofactor in this reaction [258], reviewed in [259].

Increased levels of peroxynitrite readily disrupt the dimeric eNOS complex, via oxidation and glutathionylation of the zinc-sulphur complex, of cysteine residues in the reductase domain, which leads to utilisation of oxygen as the terminal electron donor rather than arginine. In addition, peroxynitrite induces the oxidation of BH_4_, leading to its dissociation from the enzyme’s active site [260, 261]. The net effect is the formation of BH_4_-depleted eNOS monomers which produce high levels of superoxide rather than NO, which is described as uncoupling [254, 262]. The structure of eNOS is depicted in Fig. 2. For the sake of completeness, it should be noted that increased ROS can also adversely affect the activity of eNOS via the MAPK-induced phosphorylation of Thr495/Tyr657 and by stimulating increased production of asymmetric dimethylarginine (ADMA) which acts as an endogenous inhibitor of the enzyme, reviewed in [263]. Unsurprisingly, eNOS uncoupling and the generation of superoxide play a major role in the development of atherosclerotic plaques and the onset of CVD [255, 264]. Mechanistically, this is partly due to increased levels of hydrogen peroxide and superoxide production by NOX, XO, eNOS uncoupling and mitochondria, which may induce damage to DNA, lipids and proteins within the organelle leading to a cycle of ever-increasing levels of superoxide production by electron transport chain (ETC) enzymes coupled with ever-increasing bioenergetic decline and increasing hydrogen peroxide levels in the cytosol [249, 250, 255, 263].

We now move to discuss the pathological consequences of excessive mitochondrial ROS (mtROS) production in ECs and elsewhere.

### Increasing levels of mtROS

#### Overview

Excessive levels of mtROS production are causatively associated with the pathogenesis of EC senescence [72, 265, 266], the development of EC dysfunction [267, 268] and in the development of atherosclerosis [269, 270]. The importance of mitochondrial dysfunction in the genesis of atherosclerosis is emphasised by data suggesting that the severity of atherosclerosis in humans correlates with the level of mtROS production in ECs [271]. Clearly, induced EC cell senescence is one factor explaining the relationship between excessive mtROS and increased development of EC dysfunction and accelerated atherosclerosis [72, 272], reviewed in [107]. However, other factors are also involved which we discuss below.

#### Compromised mitochondrial dynamics

mtROS is a major cause of compromised mitochondrial dynamics, typified by an imbalance between mitogenesis and mitophagy, accompanied by increased levels of fission and decreased levels of fusion, leading to a disruption of networks and fragmentation of individual mitochondria [273–276]. This is of major pathophysiological importance as mitochondria perform essential roles in EC signalling affected by changes in mitochondrial dynamics in response to environmental cues [277, 278]. Unsurprisingly, defects in mitochondrial dynamics are causatively associated with increased EC activation and dysfunction [279, 280].

#### Loss of calcium homeostasis

Excessive levels of mtROS production may be a source of dysregulated calcium homeostasis resulting in a distinctive pattern of increased intramitochondrial Ca^2+^ in mitochondria and a loss of Ca^2+^ from the ER [266, 281, 282]. This is of importance as this pattern of Ca^2+^ distribution within EC mitochondria also plays a major role in the development of EC senescence [266, 281, 282]. The increase in intramitochondrial calcium ions may also exert detrimental effects on ATP production and lead to further increases in mtROS production, creating a spiral of ever-increasing mitochondrial dysfunction, reviewed in [58]. Given the importance of disturbed mitochondrial dynamics in the genesis of EC dysfunction, it should be noted that elevated mitochondrial calcium ion levels regulate many aspects of mitochondrial dynamics, such as organelle biogenesis and motility, via several mechanisms which include elevating the expression of PGC-1α and increasing mitochondrial fission [283–285].

#### Inactivation of SIRT-1-mediated signalling pathways

Excessive levels of mtROS production and elevated cytosolic ROS also decrease the activity of sirtuins (SIRTs), most notably SIRT-1 [286, 287]. This is pertinent as these deacetylases normally play an important role in inhibiting the development of senescence in vascular ECs, reviewed in [288]. The inactivation of SIRTs may also be another factor in the development of compromised mitochondrial dynamics, mitophagy and mitogenesis via reduced activity of PGC-1α and PPAR-γ [289, 290]. Impaired activity of this coactivator and transcription factor can also promote disturbances in mitochondrial dynamics by preventing the upregulation of UCP-2 [291, 292]. The activity of this protein is important in maintaining mitochondrial networks and preventing mitochondrial dysfunction via the activation of p53 [293]. Unsurprisingly, UCP-2 also plays an important role in maintaining EC function and the prevention of senescence in an environment of oxidative stress [294, 295]. Readers interested in a more comprehensive explanation of the factors involved in preventing and inducing the development of EC senescence are invited to consult the following reviews [107, 265].

Clearly, excessive levels of mtROS production exert several pathological consequences as outlined above. We now consider the pathological consequences of increased mtROS and NOX in the cytosol, which may result in dysfunctional cellular signalling normally regulated by physiological levels of hydrogen peroxide and NO, leading to a loss of redox homeostasis.

## Loss of cellular redox homeostasis

### Physiological and pathological roles of hydrogen peroxide signalling

In the absence of ONS, cytosolic hydrogen peroxide, ultimately derived from the activity of the ETC and NOX enzymes, plays an indispensable role in the regulation of cellular signalling pathways and redox homeostasis [296], reviewed in [244]. Crucially, this radical species also plays an essential role in the maintenance of EC quiescence function [297–299]. Hydrogen peroxide signalling also plays a vital role in fostering cell survival in an environment of increasing ONS via the activation of several kinases including PI3/Akt [300].

These roles are mainly affected by the two-electron oxidation of cysteine thiolate anions to sulfenic acid which may then form intramolecular or extramolecular disulphide bonds or undergo further oxidation to sulfenic acid. Readers interested in a detailed consideration of the biochemistry and thermodynamic parameters involved are invited to consult the work of [244, 301]. However, there are two key points to make from the perspective of this paper. First, these oxidative modifications act as redox switches changing the activity, function and location of proteins and enzymes in a hydrogen peroxide concentration-dependent manner, which in turn affect the performance of signalling systems as the cellular redox environment changes [244]. Second, within physiological limits, these modifications are reversible and are recovered by anti-oxidant enzymes and systems such as the thioredoxin glutathione systems, with peroxiredoxins and glutaredoxins playing prominent roles [297, 302].

However, in an environment of chronic ONS, increasing hydrogen peroxide levels have pathogenic consequences, not least by inducing over-oxidation of crucial functional cysteine groups in the thioredoxin [58, 303] and glutathione systems, potentially rendering both systems inactive [304]. It should be noted that such inactivation may be reversible if caused by oxidation of thiolate anions to sulfenic acid but the weight of evidence suggests that the oxidation of the latter to sulfonic acid is not [305]. This essentially permanent disruption of redox-based cellular signalling may be one factor explaining the relatively disappointing responses achieved by anti-oxidant therapy in neurodegenerative and neuroprogressive illnesses. The loss of redox homeostasis and increasing levels of hydrogen peroxide may also be accompanied by increased activity of NF-κB.

### Pathological consequences of elevated NF-κB

Over time, excessive levels of hydrogen peroxide induce the activation of NF-κB in ECs and other tissues [246, 306]. This is a major driver of EC senescence and activation [307, 308], reviewed in [309]. As previously discussed, one major cause of endothelial activation is inhibition of KLF-2 and KLF-4 and readers interested in a detailed consideration of the various streams of pathology flowing from this scenario and the complicated interplay between these transcription factors are invited to consult the work of [310, 311]. However, it should be noted that NF-κB can induce EC dysfunction via a number of other routes including the disruption of EC fatty acid metabolism and by stimulating the switch in energy production from oxidative phosphorylation to energy production via glycolysis [312–314], reviewed in [315].

There is also a wealth of evidence from in vivo and in vitro studies reporting a causative association between elevated levels of NF-κB activity and increased transcription of inducible nitric oxide synthase (iNOS) leading to excessive production of NO in the intracellular and extracellular environments [316, 317]. This association is unsurprising given that the promoter region of the iNOS gene possesses several NF-κB binding sites and given the promiscuous nature of NF-κB as a transcription factor [318]. The pathological consequences of increased levels of NO are well documented, not least by acting as a source of increased peroxynitrite production, as highlighted above. Increased levels of this radical also lead to compromised cellular redox signalling by dysregulating the S-nitrosylation of proteins, which is an abnormality playing a causative role in the development of CVDs and also appears to play a role in the pathogenesis of neurodegenerative and neuroprogressive illnesses [319–321].

In addition, NF-κB plays a major role in the activation of atherogenic and inflammatory miRNAs which are known to play an important role in the development of EC dysfunction and atherosclerosis in an environment of ONS [322], reviewed in [323]. This is achieved by stimulating the transcription of these miRNAs and via a more general role in initiating, maintaining and amplifying the production of PICs, RNS and ROS [290, 324]. Hence, the activation of NF-κB would appear to be a pivotal event leading to the disruption of NO-mediated redox signalling and a significant increase in the EC population of atherogenic miRNAs which have a range of pathological consequences relevant to the central theme of this paper. Therefore, we will consider each in turn, beginning with the effects of disrupted S-nitrosylation.

### Compromised S-nitrosylation and the development of hypernitrosylation

In physiological conditions, reversible S-nitrosylation is the other major player regulating the activity of redox-sensitive proteins, enzymes and signalling pathways. The basic mechanisms involved are reviewed in [325, 326]. Increased protein nitrosylation is initially a defensive response to increased levels of oxidative stress and plays a vital role in maintaining conformation and function in such an environment [319].

However, in the face of pathological increases in RNS and ROS levels, the mechanisms responsible for maintaining the reversibility of S-nitrosylation break down leading to a state described as protein hypernitrosylation [327]. This is important from a pathological perspective as levels of protein S-nitrosylation regulate many specific EC functions including tight junction permeability, inflammatory status and survival, reviewed in [328]. More specifically, excessive and irreversible S-nitrosylation in ECs is associated with disturbed fatty acid metabolism and compromised ETC function as evidenced by reduced activity of complexes I, III and IV [329, 330]. Evidence suggests that a state of hypernitrosylation is also a major cause of EC dysfunction and activation [319, 331]. One cause of such EC activation, driven by high levels of protein S-nitrosylation, appears to be loss of the normal level of association between VE-cadherin and beta-catenin and compromised small GTPase activity [332, 333]. Excessive and irreversible S-nitrosylation also disrupts mRNA splicing and translation in ECs, resulting in a dysfunctional proteome [331].

### Increased transcription of atherogenic miRNAs

The transcription and activity of miRNAs are influenced by changes in the methylation and histone acetylation status of DNA within the promoter regions of genes encoding their production. Thus, data confirming that many atherogenic mechanosensitive miRs are upregulated in a cellular environment dominated by excessive levels of ROS, RNS and PICs is unsurprising. Crucially, this scenario applies to miR-92a, which is activated by elevated levels of hydrogen peroxide [334, 335] and is widely regarded as an indispensable player in the development of atherosclerosis mediated either by disturbed flow or by increased oxidative stress. There is also accumulating evidence to suggest that other members of the miR-92 cluster, such as miR-92b, are upregulated in an environment of upregulated ROS production [336–338]. This is of interest as several transcripts of the miR-17-92 cluster appear to reduce the activity of KLFs and may well play an under-discussed role in the development of endothelial dysfunction in inflammatory conditions [152]*.* miR-34 is another KLF inhibitor upregulated by high levels of hydrogen peroxide [154, 339, 340]. This is also true of miR-200 [151, 341, 342], and it also plays a role in inhibiting KLFs [151]. Additionally, this miR may also encourage the development of atherosclerosis via a mechanism involving the disruption of the SIRT-1-FOXO3a signalling pathway which normally operates to limit ROS production in ECs likely by inhibiting the assembly and activation of NOX [342–344]. It should be emphasised that disrupted SIRT-1 signalling is an important element in the development of CVD [345]. miR-155 is another KLF inhibitor playing an important role in EC cell dysfunction induced in an environment of chronic inflammation, although its activation in these conditions appears to be secondary to elevated NF-κB rather than upregulated ROS [152, 346, 347].

miR-146 is yet another miRNA which plays a role in inhibiting KLFs and is also upregulated as a result of increased NF-κB activity [347, 348]. There is also evidence to suggest that this miR may be directly upregulated by ROS-mediated demethylation of DNA within the promoter region of the encoding gene [349]. miR-146 also belongs to a class of miRs described as “mitomiRs” whose upregulation can disturb the expression of mitochondrial genes governing the performance of the ETC leading to upregulated ROS production, compromised energy production and damage to functional and/or structural proteins within the organelle [350, 351]. This is of particular importance from the perspective of this paper as the activation of this group of miRs is considered to be a major element in the genesis and maintenance of EC senescence, reviewed in [351].

Thus far, we have suggested several abnormalities which could account for high rates of endothelial dysfunction and atherosclerosis in patients with neuroprogressive disorders in an environment of relatively reduced cholesterol. However, we have not considered evidence which demonstrates whether these abnormalities have actually been reported in patients with these illnesses. Hence, this omission will be addressed in the next section before considering treatment approaches.

## Potential generators of EC dysfunction and activation in MDD, BPD and SZ

### NF-κB

Elevated activity of NF-κB has been reported in the plasma and peripheral blood mononuclear cells (PBMCs) of patients with first-episode SZ before the onset of any treatment [352, 353]. Increased levels of NF-κB expression and activity have also been repeatedly reported in these compartments in patients subject to a diagnosis of MDD and BPD whether in the symptomatic phases of their illness or during remission [354, 355].

### Platelet activation

Increased inflammation-mediated PA, as measured by increased platelet volume, has also been repeatedly reported in patients with MDD [356–358]. There is also extensive evidence of PA in patients with BPD compared with healthy controls [359–361]. There are also data to suggest that the level of PA may be greater in patients with acute mania compared with patients in the depressive or euthymic phases of the illness [362]. The picture appears to be less clear in patients with SZ, however, likely due to the effects of anti-psychotic medication which may suppress at least some signalling pathways involved in stimulating PA [363]. That being said, there is accumulating evidence to suggest that PA may be increased in at least some treatment-naïve first-episode patients [360, 364, 365].

### Atherogenic miRs

Many of the miRs known to play a causative role in the development of EC dysfunction and atherosclerosis are also upregulated in many patients with neuroprogressive illnesses. For example, upregulation of miR-34 has been reported in drug-free MDD, BPD and SZ patients [366]. There is also evidence of upregulated miR-146 and miR-200 activity in patients with MDD and BPD, reviewed in [367]. There would appear to be no evidence that this is the case in SZ, however, although a recent review suggested that the expression of miR-92 was upregulated, or at least dysregulated, in some first-episode patients [368].

### Myeloperoxidase

Increased plasma MPO activity is another common finding in patients with MDD [369, 370], BPD [227, 371] and SZ [372]. In addition, there is some evidence to suggest that increased levels of this enzyme may be involved in the pathophysiology of neuroprogressive illnesses and may be a state marker in MDD [370] and BPD [371]. However, this apparent association may be because an elevated level of MPO is an accepted marker of systemic inflammation and oxidative stress, as noted above.

### Xanthine oxidase and uric acid

Many studies have produced copious evidence of increased XO activity and high levels of UA in the circulation in all phases of BPD [373–375], although serum UA levels appear to be at their highest in mania [58]. This may be a consequence of increased levels of inflammation in this phase of the illness compared with euthymia and depression, reviewed in [58]. Several prospective studies and meta-analyses have also confirmed an improvement in the symptoms of mania following XO inhibition via allopurinol [375, 376]. There have also been reports of increased XO activity in the brain of at least some SZ patients [377, 378], and there have been several reports of increased XO activity in patients in the periphery [379–381]. However, levels of XO activity and UA in the serum appear to be low in first-episode drug-naïve SZ patients [382, 383]. The situation in MDD is also mixed in that there is some evidence of increased XO activity in the brain and periphery of some MDD patients [384, 385] and there has been a report that high UA levels in MDD patients are predictive of a transition to bipolarity [386]. However, once again, the weight of evidence suggests that serum UA is low in the majority of MDD patients [387, 388].

The findings in patients presenting with first-episode SZ cited above are somewhat surprising as evidence suggests that the high levels of inflammation and oxidative stress reported in such individuals should promote the conversion of xanthine dehydrogenase to XO and increase levels of the latter [192, 389]. However, this apparent paradox might be explained by the high levels of allantoin reported in first-episode treatment-naïve SZ patients, which suggests increased UA oxidation [390].

Briefly, unlike other mammals, humans do not possess urate oxidase, and hence, allantoin production can only result from the action of oxidants. In this case, it should be noted that UA is very vulnerable to oxidation by peroxynitrite with the resultant production of a range of highly cytotoxic radicals whose role in pathology appears to be under-discussed [391, 392]. Hence, low UA levels may be detected in first-episode patients even with reasonable levels of XO activity. This proposal seems acceptable given evidence of high peroxynitrite activity in the plasma of such individuals [393]. There is also evidence to suggest that disturbed purine catabolism evident in first-episode treatment-naïve patient’s results in reduced levels of xanthene, which would also explain low UA levels even with relatively high levels of active XO [382, 383]. It should also be noted that UA is responsible for some 60% of radical scavenging capacity in blood, and thus, it is not difficult to conceive of a scenario in which high levels of ROS seen in many patients with MDD would lead to depleted UA levels in an environment of activated XO [382, 394]. A literature search fails to reveal any evidence of published research investigating circulating allantoin levels in MDD patients which could add support or otherwise for this proposition.

### Inflammation, oxidative stress, nitrosative stress and mitochondrial dysfunction

There is extensive evidence of increased inflammatory markers such as TNF-α and C-reactive protein (CRP) in the tissues of patients with SZ [31–33], BPD [34–37] and MDD [38–41]. The existence of elevated ROS and RNS, and a compromised anti-oxidant response network in the blood and tissues of these patients, has also been demonstrated beyond reasonable doubt [54–58]. The presence of gross mitochondrial dysfunction in the peripheral tissues, platelets and PBMCs of patients with MDD, BPD and SZ has also been repeatedly demonstrated [58–61].

### Interdependency of endothelial dysfunction and inflammation

There is extensive evidence of impaired vascular dysfunction, inflammation and senescence in MDD patients. This includes high levels of sICAM-1 and VCAM-1 [395, 396] von Willebrand factor (vWF) [397–399] and elevated levels of TNF-α, IL-6 and C-reactive protein, reviewed in [400]. In addition, vWF may be considered as a trait marker for MDD as this molecule is consistently higher in patients with depression irrespective of anti-depressant (AD) status [397, 399]. Furthermore, a strong positive correlation between sICAM and sVCAM-1 levels and the extent of white matter hyperintensities in MDD patients has been reported suggesting a causative role of endothelial dysfunction in the development of the illness [395]. This proposition is supported by a study reporting a positive and robust correlation between the extent of vascular inflammation and arterial stiffness and the severity of depressive symptoms and dysfunction [401], reviewed in [402]. Understandably, there has been a great deal of research into the causes of endothelial dysfunction in MDD and most evidence suggests that it may be due at least in part to increased NADPH oxidase-mediated superoxide levels in ECs and a subsequent reduction in NO-mediated vasodilation [403, 404], reviewed in [405].

Numerous research teams have provided evidence of endothelial activation inflammation and dysfunction in BPD irrespective of the phase of the illness. Levels of dysfunction may however vary between patients in the depressive euthymic and depressive states of this psychiatric illness and during the course of the illness [78, 396, 406]. For example, BPD patients in a later, progressive stage of disease display significantly higher levels of sICAM-1 levels compared to individuals in an earlier stage of their illness [407]. High sICAM levels are found in the manic and depressive phases of BPD suggesting that sICAM-1 may be a trait marker [396]. However, there is evidence to suggest that sICAM levels are higher in mania than the depressive phase of the illness, indicating that sICAM could also be a useful state marker in the illness [408]. Furthermore, levels of endocan and urotensin-II, which are markers of EC senescence and activation, respectively [409, 410], are higher in patients with acute mania than those in the euthymic state which in turn were higher than healthy controls [78]. Increased endothelial cell activation in mania is suggestive of elevated levels of NF-κB which is consistent with data demonstrating higher levels of inflammation and oxidative stress in mania compared to other phases of the illness—reviewed in [58]. Hence, the level of endothelial dysfunction seen in BPD may be related to high levels of systemic PICs, ROS and RNS.

There is extensive evidence of endothelial dysfunction and inflammation in many patients with SZ which includes high levels of sICAM-1, sVCAM1 and vWF in the periphery and high levels of VCAM-1, VE-cadherin and a range of tight junction proteins in the brain [411–415]. However, there is increasing evidence that endothelial activation and dysfunction may be confined to or at least be greatly enhanced in an inflammatory subtype of schizophrenia [411, 415]. However, in these latter patients, levels of vWF display a robust and positive correlation with disease severity [412, 416] and increase during psychotic episodes [413]. Finally, it is noteworthy that there is an inverse linear relationship between vWF levels and basal ganglia volume that strongly suggests the involvement of inflammation-mediated endothelial damage in the pathophysiology of the syndrome in at least some patients [79, 412].

### Effects of anti-depressants and anti-psychotic therapy on endothelial function

Several authors of large prospective studies have reported significantly reduced cardiovascular events in MDD patients who responded to antidepressant therapy (AD) compared to those who did not [417, 418]. Decreased platelet activity is another replicated finding in MDD patients in remission following prolonged AD consumption [419, 420]. Responders to AD display significant improvements in markers of endothelial dysfunction and inflammation as measured by increased flow-dependent endothelial-mediated dilation and decreased levels of IL-6 [397, 419, 421, 422]. For example Lopez-Vilchez and fellow workers’ reported endothelial inflammation and significant endothelial damage and inflammation in their trial participants at diagnosis which normalised following treatment with escitalopram for 24 weeks which suggests that endothelial dysfunction in MDD patients is reversible [397]. It is also noteworthy that the weight of evidence suggests that vascular and hemodynamic parameters improve in responders to AD therapy irrespective of cardiovascular risk or the consumption of medicines aimed at treating blood pressure known to impact vascular function [423]. ADs may not be equally effective in improving endothelial dysfunction however, and there is some evidence to suggest that improved endothelial function may be greater in males than females [424].

There is also some evidence to suggest that the benefits of low-dose lithium in BPD and stroke may arise in part from improved endothelial function and decreased levels of EC inflammation and death [425–427]. There is also some suggestion that this may be true of valproate and lamotrigine [428]. However, the data regarding valproate is mixed and there is evidence that this drug compromises endothelial function in many patients—reviewed in [429]. The data regarding the use of atypical psychotics in SZ looks equally bleak with accumulating evidence suggesting that atypical anti-psychotics have a detrimental effect on endothelial function [430, 431], reviewed in [432]. However, some authors have reported decreased levels of iCAM-1 in SZ patients following administration of atypical anti-psychotics suggesting that the effects of these drugs on the vascular endothelium are more complex than is generally appreciated [433], reviewed in [396].

### Socioeconomic behavioural and psychosocial factors in the development of CVD

Several behavioural psychosocial and socioeconomic factors are associated with increased risk of CVD [434–436]. Socioeconomic disadvantage (SED) is associated in longitudinal studies with significantly increased levels of T2D, obesity, MDD, anxiety, BPD, SZ and hypertension in adolescence and later adulthood [437–441]. These illnesses are all associated with high levels of vascular senescence [107, 442–444] and high levels of systemic inflammation reviewed in [56]. This is of relevance as endothelial cell senescence [73] and low-grade systemic inflammation [445, 446] play important causative roles in the development and acceleration of CVD. Hence, increased systemic low-grade inflammation and endothelial senescence in childhood and adolescence go some way to explaining the association between SED in children and increased risk of CVD in adulthood.

Several studies have reported a significant causative association between increased low-grade systemic inflammation and high-fat, high-carbohydrate diets [447, 448], sedentary behaviour or suboptimal physical activity [449, 450], lack of sleep [451, 452], smoking [453, 454] and alcohol consumption [455]. This contributes to an understanding of the various lifestyle risk factors operative during childhood and adolescence and the development of CVD.

Bipolar mania or depression and MDD are independently associated with increased endothelial dysfunction as assessed by the reactive hyperaemia index and flow-mediated dilation (FMD) [456, 457]. In addition, behaviours such as smoking, diet, physical activity and social interactions all influence the risk of developing MDD, BPD and SZ [458, 459]. Furthermore, these behaviours appear to be more common in socioeconomically disadvantaged children and adolescents [436]. These findings illustrate the complexity and interdependence of the factors underpinning experiences during childhood and behaviours during childhood and adolescence and future CVD risk and the need for holistic remedial measures. In addition, there is evidence to suggest that the behaviours and psychological distress associated with socioeconomic deprivation may result from limited life choices and are in many cases not related to personality or any underlying psychological abnormalities [460]. It is however interesting to note that the association between socioeconomic deprivation and CVD may stem from perceived rather than objective socioeconomic status [461]. In fact, there is data suggesting that lower subjective social status is associated with impaired EC function and vasodilation rather than objective measures of income and education [462, 463].

Finally, several authors have reported significant associations between type D personality (negative affectivity and social inhibition) [464, 465] and depressive or irritable temperament [466, 467]. The causes of these associations are not fully understood, but in the case of type D personality, adverse lifestyle choices predictive of CVD development appear to be an important factor while irritable temperament is predictive of increased vascular stiffness and hypertension [465, 468, 469]. There is also an argument that a type D personality and/or an irritable temperament make an individual more susceptible to the effects of environmental stressors and experience the physiological effects of chronic stress such as increased glucocorticoid receptor resistance [470]. This is relevant from the perspective of increased CVD risk as this state is an acknowledged cause of endothelial dysfunction and activation [471, 472]. Prolonged elevation of glucocorticosteroids also compromises endothelial function via several other routes such as increasing production of EC, hydrogen peroxide and superoxide levels and reducing the production of endothelial nitric oxide synthase [473, 474].

### The origin of endothelial dysfunction in mental illnesses—a working hypothesis

High levels of IL-6, IL-1 and TNF-α are commonly reported in patients with MDD and BPD both in the periphery and in the brain [475–478]. This is also true of many patients with SZ [60, 411]. In addition, there is a consensus that oxidative and nitrosative stress is involved in the pathophysiology of all three illnesses as previously discussed [58, 60]. This is relevant as numerous research teams have described that increased endothelial cell activation is stimulated by increased levels of ROS and RNS [479, 480]. Increased levels of TNF-α, IL-6 and IL-1 are also well-documented causes of increased activation, senescence and dysfunction of the vascular endothelium [481, 482]. In addition, ADs reduce levels of TNF-α, IL-6 and other PICs [483, 484]. They also reduce levels of ROS while stimulating enzymatic and non-enzymatic anti-oxidant systems [485, 486]. Hence, the improvements in vascular function following AD therapy may be related to reduced levels of inflammation and oxidative stress. In addition, lithium reduces oxidative stress and inflammation [487], suggesting that the proposed benefits of the drug on endothelial function may also be due to reduced levels of PICs, ROS and RNS. Thus, it seems reasonable to propose that the endothelial dysfunction seen in MDD, BPD and SZ is a consequence of the oxidative stress and inflammation attribute of the illnesses.

We tentatively suggest that the initial stages of endothelial dysfunction and activation are instigated by TNF-α and ROS which are secreted by activated macrophages. These appear to be involved in the pathophysiology of mental illnesses at a very early stage in their development [488, 489]. High levels of TNF-α and the ROS superoxide and hydroxyl radicals are vital elements in the development of ECs as they play a dominant role in degrading the protective glycolax layer lining the luminal side of the vascular endothelial layer which otherwise protects these cells against the effects of inflammatory mediators [490–492].

Once exposed, ECs are vulnerable to activation and/or damage resulting from engagement of TNF-α, IL-1β and IL-6 with their cognate receptors [490, 493]—reviewed in [494]. The resultant activation of NF-κB produces the same range of pathological consequences as seen in the process of atherosclerosis instigated by adverse changes in arterial blood flow described above. There is much evidence to support this process as an initial cause of endothelial dysfunction in mental illnesses and cytokine- and ROS-mediated atherosclerosis is now recognised as a separate endophenotype distinct from atherosclerotic processes exacerbated by dyslipidaemia [495].

However, despite our current working hypothesis, we would caution against the view of endothelial dysfunction as an epiphenomenon in MDD, BPD and SZ. In fact, there is extensive evidence that endothelial activation and dysfunction may play a major role in the development and exacerbation of systemic inflammation and increased activation of the coagulation cascade in a process described as immunothrombosis, and hence, an activated endothelium is likely to play a pathophysiological role in each of these illnesses [490, 496].

## Treatment suggestions

### Statins

There is extensive evidence of improved eNOS function following statin administration [497, 498]. However, the bulk of such evidence originates from in vitro studies involving levels far exceeding doses used in clinical studies [499]. In addition, there appears to be little or no benefit on eNOS function when a statin is administered in vivo [500]. Reports of decreased eNOS activity and NO levels, either during therapy or following discontinuation, are also a concern [501, 502]. However, there is extensive evidence of improved endothelial function in individuals prescribed statins for hyperlipidaemia and peripheral vascular disease and in patients at high risk due to cigarette consumption [500–504]. Unfortunately, this effect does not appear to extend to individuals with normal levels of cholesterol [500, 502, 503]. Similarly, evidence provided by research teams investigating the in vivo effect of statin administration on platelet function suggests that any measurable benefit is limited to ADP-stimulated PA in patients with hyperlipidaemia [505, 506]. Despite such ambiguity, evidence that in vivo administration of statins produces a significant reduction in MPO levels in conditions such as T2D and acute coronary syndrome is encouraging, although there does not seem to be any published evidence regarding statin-mediated effects on MPO levels in patients with normal levels of cholesterol [507, 508]. In addition, there are data, albeit from animal and in vitro studies, to suggest that statin therapy might upregulate levels of KLF-4 and/or KLF-2, which is clearly a desirable therapeutic attribute [509–511]. However, there are also reports of decreased KLF-2 and KLF-4 following statin administration [509–511]. There is also some suggestion that statin-induced muscle damage may be mediated, at least in part, by increasing the activity of XO [512].

More positively, several research teams have reported significant decreases in CRP levels following prolonged statin therapy, which is significant as CRP levels are a powerful predictor of myocardial infarction [513–515]. There is also some evidence to suggest that statin usage reduces circulating levels of TNF-α in patients with hyperlipidaemia and may also reduce activity of NF-κB in established CVD [516–518]. The situation as far as the management of oxidative stress is concerned appears to be more uncertain, however, with a suggestion that lipid peroxidation may be alleviated in the circulation following statin therapy, but levels of ROS may well be increased. For example, a recent meta-analysis concluded that malondialdehyde (MDA) levels in the bloodstream were reduced in patients with hyperlipidaemia compared with pre-treatment levels [519]. However, this does not appear to be the case in people with normal cholesterol levels [520]. Furthermore, there is accumulating evidence to suggest that statin usage increases mtROS production leading to elevated levels of circulating and intracellular hydrogen peroxide [521, 522], reviewed in [523].

There is also accumulating evidence suggesting that statin usage inhibits the enzymes of the ETC in muscle cells and elsewhere leading to significant levels of mitochondrial dysfunction and increasing levels of hydrogen peroxide [521, 524]. There is also increasing concern about statin toxicity [525] and increased risk of developing T2D [526, 527]. The data associating statin usage with increased oxidative stress and mitochondrial dysfunction clearly give pause for thought as oxidative stress and mitochondrial dysfunction are thought to play a pivotal role in the pathophysiology of neuroprogressive illnesses, as discussed above.

The mechanisms explaining statin-induced mitochondrial dysfunction are not fully understood but would appear to involve other factors in addition to inhibition of the ETC, such as compromised AMPK- and mTOR-regulated signalling pathways [521, 524]. Perhaps most importantly, this phenomenon also appears to be due to impaired synthesis of coenzyme Q_10_ (CoQ) [528, 529]. The potentially paramount importance of this latter mechanism is emphasised by evidence that statin-induced myopathy, resulting from induced mitochondrial dysfunction, may be greatly ameliorated or even extinguished following supplementation with CoQ [530, 531]. Hence, combining the use of statins and CoQ would appear to be advisable if the former is administered to the patients described in this paper. There is also the potential for therapeutic synergy between the two preparations as far as relieving endothelial dysfunction and inhibiting the development or progression of atherosclerosis is concerned, reviewed in [532]. Hence, we consider this proposal below.

### Coenzyme Q_10_

There is a substantial and accumulating body of evidence suggesting significant and relatively large improvements in endothelial function following CoQ administration in individuals with established CVD and those who are CVD-free [533], reviewed in [534]. Moreover, these positive effects appear to be mediated by improved mitochondrial function and the reduction in mtROS-mediated EC dysfunction and reduced EC senescence [535–538]. There are also reports of improvement in endothelial function in T2D and CVD following CoQ supplementation in patients optimally treated with statins [539, 540]. These data would appear to strengthen further the argument for combining these two preparations in an attempt to mitigate CVD risk in patients with neuroprogressive illnesses [539, 540]. Given the above information, it probably comes as no surprise to learn that there are several studies suggesting that CoQ arrests the development of atherosclerosis (reviewed in [541]), CVD morbidity and CVD mortality [542], although it should be noted that the results reported by such studies are somewhat inconsistent [543].

Several research teams have also reported reduced PA following CoQ supplementation, which is consistent with a potential role in mitigating the risk of developing CVD [544–546]. However, there do not appear to be any published studies investigating the effect of CoQ on XO in humans and the evidence regarding any effect on UA is mixed, with decreases and increases in circulating levels being reported [547, 548]. In addition, data supporting the use of CoQ supplementation as a means of reducing MPO levels in the circulation are currently limited to the results of a solitary study confirming a significant benefit when co-administered with n-3 polyunsaturated fatty acids (PUFAs) [549]. Furthermore, a literature search has failed to locate any published study examining the effects of CoQ on KLFs, whether in vivo or in vitro.

The anti-inflammatory effect of CoQ supplementation is well established as several recent meta-analyses and prospective studies have reported a significant decrease in PICs and surrogate markers of elevated ROS and RNS in the peripheral circulation following CoQ administration in several illnesses, most notably metabolic syndrome and multiple sclerosis [550–553]. There is also some evidence to suggest that these effects are mediated by reduced activity of NF-κB [535, 554]. Several authors have also reported significantly improved mitochondrial function following the administration of standard formulations of CoQ and a formulation modified specifically to target mitochondria and often described as mitoQ [535, 537, 538].

In addition, there is evidence to suggest that CoQ supplementation may reduce the severity of symptoms experienced by patients with BPD in the depressive phase of their illness [555, 556]. There is also a solitary study reporting a significant reduction in the negative symptoms suffered by many patients with SZ following prolonged supplementation with this quinone [557]. Furthermore, CoQ supplementation has the potential to alleviate the shortfall in the production of this molecule seen in many patients with MDD [558]. The cause of this observed phenomenon is not fully understood, but it should be noted that chronic oxidative stress is a major cause of CoQ depletion [559–561]. It should also be noted that depleted CoQ levels are an independent cause of mitochondrial dysfunction [562, 563]. This coenzyme also has an excellent record of tolerability and safety established in a plethora of long-term studies, even at levels up to 2400 mg per day [564, 565].

CoQ depletion in a cellular environment of chronic oxidative stress may also be potentially addressed with the addition of n-3 PUFAs which improves the synthesis of the former molecule [566]. There is also evidence to suggest that a combination of CoQ and n-3 PUFAs, most notably docosahexaenoic acid (DHA), or eicosapentaenoic acid (EPA), may result in synergistic benefits in the treatment of atherosclerosis [567]. This would also seem to be true of a combination of n-3 PUFAs and statins [568], reviewed in [569]. Moreover, recent data suggest that the benefits may be even greater with “triple therapy” without any significant decrease in tolerability or overall increase in side effects [570]. Hence, the examination of potential benefits of n-3 PUFA supplementation and its ability to target identified contributors to the development of endothelial dysfunction and atherosclerosis will form the final section of this paper.

### n-3 PUFAs

Several reviews and meta-analyses have highlighted significant and relatively large improvements in endothelial function following supplementation with EPA or DHA as measured by flow-mediated dilation [571–573]. The fact that this benefit would appear to apply to individuals with a high risk of developing CVD and individuals whose risk of developing such a disease appears to be normal or low is encouraging [571–573]. Data suggesting that dietary supplementation with EPA and/or DHA improves platelet function and decreases PA in low- and high-risk individuals also suggests that PUFA supplementation may be of value in addressing high CVD risk in patients with neuroprogressive illnesses [574, 575], reviewed in [576]. A solitary report of improved KLF-4 activity following DHA or EPA is also noteworthy [577]. However, there would appear to be no effect on MPO levels following supplementation with DHA, at least in healthy volunteers, although the doses of PUFAs involved were low and MPO levels were normal in the participants involved [578].

RCTs and meta-analyses involving human participants without and with evidence of underlying pathology have reported significant reductions in levels of PICs in the circulation following prolonged dietary supplementation with n-3 PUFAs [579–581]. There is extensive evidence of a significant and large reduction in levels of circulating oxidative stress markers such as MDA and isoprostanes following n-3 PUFA supplementation [582–584]. There is also evidence to suggest that EPA or DHA improves mitochondrial function and may protect mitochondrial membranes from radical-mediated damage [585, 586].

A recent large study reported a negative correlation between plasma EPA concentration and levels of IL-6 and TNF-α in patients with BPD [587]. Reduced circulating PUFA levels would also appear to be associated with increased circulating markers of inflammation and oxidative stress in patients with SZ, at least in the acute phase of their illness [588]. These results are echoed by meta-analyses reporting low circulating PUFA levels in the blood of patients with MDD, which also appears to be associated with increased production of PICs and ROS [589]. Finally, there is also accumulating evidence suggesting that DHA and/or EPA supplementation may make a significant contribution to alleviating symptoms in patients with neuroprogressive illnesses, reviewed in [590, 591].

## Conclusions

In this paper, it has been shown how systemic inflammation, oxidative stress and mitochondrial dysfunction may drive the development of endothelial dysfunction and atherosclerosis. In particular, circulating PICs and ROS may induce inflammation, oxidative stress and mitochondrial dysfunction within ECs, either directly or indirectly via inducing high levels of platelets and PA, and increased MPO and XO activity, which are independently associated with increased cardiovascular risk. The applications of these and related molecular mechanisms to MDD, BPD and SZ have been described in detail, including evidence for the role of potential generators of EC dysfunction and activation in such neuroprogressive disorders. It is recommended that the treatment suggestions based on these molecular mechanisms which are mentioned in this paper should be the subject of further, well-powered RCTs.

## Data Availability

As above, this is a review of publicly available data.

## References

[1] Wu Q, Kling JM (2016). Depression and the risk of myocardial infarction and coronary death: a meta-analysis of prospective cohort studies. Medicine (Baltimore).

[2] Gan Y, Gong Y, Tong X, Sun H, Cong Y, Dong X, Wang Y, Xu X, Yin X, Deng J (2014). Depression and the risk of coronary heart disease: a meta-analysis of prospective cohort studies. BMC Psychiatry.

[3] Correll CU, Solmi M, Veronese N, Bortolato B, Rosson S, Santonastaso P, Thapa-Chhetri N, Fornaro M, Gallicchio D, Collantoni E (2017). Prevalence, incidence and mortality from cardiovascular disease in patients with pooled and specific severe mental illness: a large-scale meta-analysis of 3,211,768 patients and 113,383,368 controls. World Psychiatry.

[4] Miller C, Bauer MS (2014). Excess mortality in bipolar disorders. Curr Psychiatry Rep.

[5] Hayes JF, Miles J, Walters K, King M, Osborn DP (2015). A systematic review and meta-analysis of premature mortality in bipolar affective disorder. Acta Psychiatr Scand.

[6] Goldstein BI, Carnethon MR, Matthews KA, McIntyre RS, Miller GE, Raghuveer G, Stoney CM, Wasiak H, McCrindle BW (2015). Major depressive disorder and bipolar disorder predispose youth to accelerated atherosclerosis and early cardiovascular disease: a scientific statement from the American Heart Association. Circulation.

[7] Crump C, Sundquist K, Winkleby MA, Sundquist J (2013). Comorbidities and mortality in bipolar disorder: a Swedish national cohort study. JAMA Psychiatry.

[8] Gale CR, Batty GD, Osborn DP, Tynelius P, Rasmussen F (2014). Mental disorders across the adult life course and future coronary heart disease: evidence for general susceptibility. Circulation.

[9] Hayes JF, Marston L, Walters K, King MB, Osborn DPJ (2017). Mortality gap for people with bipolar disorder and schizophrenia: UK-based cohort study 2000-2014. Br J Psychiatry.

[10] Aksay SS, Bumb JM, Janke C, Biemann R, Borucki K, Lederbogen F, Deuschle M, Sartorius A, Kranaster L (2016). Serum lipid profile changes after successful treatment with electroconvulsive therapy in major depression: a prospective pilot trial. J Affect Disord.

[11] Hummel J, Westphal S, Weber-Hamann B, Gilles M, Lederbogen F, Angermeier T, Luley C, Deuschle M, Kopf D (2011). Serum lipoproteins improve after successful pharmacologic antidepressant treatment: a randomized open-label prospective trial. J Clin Psychiatry.

[12] Ong KL, Morris MJ, McClelland RL, Maniam J, Allison MA, Rye KA (2016). Lipids, lipoprotein distribution and depressive symptoms: the Multi-Ethnic Study of Atherosclerosis. Transl Psychiatry.

[13] Lehto SM, Niskanen L, Tolmunen T, Hintikka J, Viinamaki H, Heiskanen T, Honkalampi K, Kokkonen M, Koivumaa-Honkanen H (2010). Low serum HDL-cholesterol levels are associated with long symptom duration in patients with major depressive disorder. Psychiatry Clin Neurosci.

[14] Parekh A, Smeeth D, Milner Y, Thure S (2017). The role of lipid biomarkers in major depression. Healthcare (Basel).

[15] Persons JE, Fiedorowicz JG (2016). Depression and serum low-density lipoprotein: a systematic review and meta-analysis. J Affect Disord.

[16] van Reedt Dortland AK, Giltay EJ, van Veen T, van Pelt J, Zitman FG, Penninx BW (2010). Associations between serum lipids and major depressive disorder: results from the Netherlands Study of Depression and Anxiety (NESDA). J Clin Psychiatry.

[17] Peterfalvi A, Nemeth N, Herczeg R, Tenyi T, Miseta A, Czeh B, Simon M (2019). Examining the influence of early life stress on serum lipid profiles and cognitive functioning in depressed patients. Front Psychol.

[18] Huang YJ, Tsai SY, Chung KH, Chen PH, Huang SH, Kuo CJ (2018). State-dependent alterations of lipid profiles in patients with bipolar disorder. Int J Psychiatry Med.

[19] Wysokinski A, Strzelecki D, Kloszewska I (2015). Levels of triglycerides, cholesterol, LDL, HDL and glucose in patients with schizophrenia, unipolar depression and bipolar disorder. Diab Metabol Syndrome.

[20] Su M, Li E, Tang C, Zhao Y, Liu R, Gao K (2019). Comparison of blood lipid profile/thyroid function markers between unipolar and bipolar depressed patients and in depressed patients with anhedonia or suicidal thoughts. Mol Med.

[21] Huang JT, Wang L, Prabakaran S, Wengenroth M, Lockstone HE, Koethe D, Gerth CW, Gross S, Schreiber D, Lilley K (2008). Independent protein-profiling studies show a decrease in apolipoprotein A1 levels in schizophrenia CSF, brain and peripheral tissues. Mol Psychiatry.

[22] Misiak B, Stanczykiewicz B, Laczmanski L, Frydecka D (2017). Lipid profile disturbances in antipsychotic-naive patients with first-episode non-affective psychosis: a systematic review and meta-analysis. Schizophr Res.

[23] Pillinger T, Beck K, Stubbs B, Howes OD (2017). Cholesterol and triglyceride levels in first-episode psychosis: systematic review and meta-analysis. Br J Psychiatry.

[24] Zhang J, Chen L, Delzell E, Muntner P, Hillegass WB, Safford MM, Millan IY, Crowson CS, Curtis JR (2014). The association between inflammatory markers, serum lipids and the risk of cardiovascular events in patients with rheumatoid arthritis. Ann Rheum Dis.

[25] Myasoedova E, Crowson CS, Kremers HM, Roger VL, Fitz-Gibbon PD, Therneau TM, Gabriel SE (2011). Lipid paradox in rheumatoid arthritis: the impact of serum lipid measures and systemic inflammation on the risk of cardiovascular disease. Ann Rheum Dis.

[26] Lind L, Lithell H (1994). Impaired glucose and lipid metabolism seen in intensive care patients is related to severity of illness and survival. Clin Intensive Care.

[27] Gabay C, Kushner I (1999). Acute-phase proteins and other systemic responses to inflammation. N Engl J Med.

[28] Popa C, Netea MG, van Riel PL, van der Meer JW, Stalenhoef AF (2007). The role of TNF-alpha in chronic inflammatory conditions, intermediary metabolism, and cardiovascular risk. J Lipid Res.

[29] Feingold KR, Grunfeld C (2016). Effect of inflammation on HDL structure and function. Curr Opin Lipidol.

[30] Khovidhunkit W, Kim MS, Memon RA, Shigenaga JK, Moser AH, Feingold KR, Grunfeld C (2004). Effects of infection and inflammation on lipid and lipoprotein metabolism: mechanisms and consequences to the host. J Lipid Res.

[31] Fillman SG, Weickert TW, Lenroot RK, Catts SV, Bruggemann JM, Catts VS, Weickert CS (2016). Elevated peripheral cytokines characterize a subgroup of people with schizophrenia displaying poor verbal fluency and reduced Broca's area volume. Mol Psychiatry.

[32] Boerrigter D, Weickert TW, Lenroot R, O'Donnell M, Galletly C, Liu D, Burgess M, Cadiz R, Jacomb I, Catts VS (2017). Using blood cytokine measures to define high inflammatory biotype of schizophrenia and schizoaffective disorder. J Neuroinflammation.

[33] Momtazmanesh S, Zare-Shahabadi A, Rezaei N. Cytokine alterations in schizophrenia: an updated review. Front Psychiatry. 2019;10(892). 10.3389/fpsyt.2019.00892 PMC6915198 31908647

[34] Modabbernia A, Taslimi S, Brietzke E, Ashrafi M (2013). Cytokine alterations in bipolar disorder: a meta-analysis of 30 studies. Biol Psychiatry.

[35] Brietzke E, Stertz L, Fernandes BS, Kauer-Sant'anna M, Mascarenhas M, Escosteguy Vargas A, Chies JA, Kapczinski F (2009). Comparison of cytokine levels in depressed, manic and euthymic patients with bipolar disorder. J Affect Disord.

[36] Munkholm K, Vinberg M, Vedel Kessing L (2013). Cytokines in bipolar disorder: a systematic review and meta-analysis. J Affect Disord.

[37] Goldsmith DR, Rapaport MH, Miller BJ (2016). A meta-analysis of blood cytokine network alterations in psychiatric patients: comparisons between schizophrenia, bipolar disorder and depression. Mol Psychiatry.

[38] Maes M, Berk M, Goehler L, Song C, Anderson G, Galecki P, Leonard B (2012). Depression and sickness behavior are Janus-faced responses to shared inflammatory pathways. BMC Med.

[39] Berk M, Williams LJ, Jacka FN, O’Neil A, Pasco JA, Moylan S, Allen NB, Stuart AL, Hayley AC, Byrne ML (2013). So depression is an inflammatory disease, but where does the inflammation come from?. BMC Med.

[40] Chen LP, Dai HY, Dai ZZ, Xu CT, Wu RH (2014). Anterior cingulate cortex and cerebellar hemisphere neurometabolite changes in depression treatment: a 1H magnetic resonance spectroscopy study. Psychiatry Clin Neurosci.

[41] Zou W, Feng R, Yang Y (2018). Changes in the serum levels of inflammatory cytokines in antidepressant drug-naive patients with major depression. PLoS One.

[42] Taleb S (2016). Inflammation in atherosclerosis. Arch Cardiovas Dis.

[43] Bäck M, Yurdagul A, Tabas I, Öörni K, Kovanen PT (2019). Inflammation and its resolution in atherosclerosis: mediators and therapeutic opportunities. Nat Rev Cardiol.

[44] Liu Y, Tie L (2019). Apolipoprotein M and sphingosine-1-phosphate complex alleviates TNF-alpha-induced endothelial cell injury and inflammation through PI3K/AKT signaling pathway. BMC Cardiovasc Disord.

[45] Panchal SK, Brown L. Cholesterol versus inflammation as cause of chronic diseases. Nutrients. 2019;11(10). 10.3390/nu11102332 PMC6835531 31581553

[46] Ridker PM, Everett BM, Thuren T, MacFadyen JG, Chang WH, Ballantyne C, Fonseca F, Nicolau J, Koenig W, Anker SD (2017). Antiinflammatory therapy with canakinumab for atherosclerotic disease. N Engl J Med.

[47] Moriya J (2019). Critical roles of inflammation in atherosclerosis. J Cardiol.

[48] Yang X, Li Y, Li Y, Ren X, Zhang X, Hu D, Gao Y, Xing Y, Shang H (2017). Oxidative stress-mediated atherosclerosis: mechanisms and therapies. Front Physiol.

[49] Kattoor AJ, Pothineni NVK, Palagiri D, Mehta JL (2017). Oxidative stress in atherosclerosis. Curr Atheroscler Rep.

[50] Marchio P, Guerra-Ojeda S, Vila JM, Aldasoro M, Victor VM, Mauricio MD (2019). Targeting early atherosclerosis: a focus on oxidative stress and inflammation. Oxidative Med Cell Longev.

[51] Madamanchi NR, Runge MS (2007). Mitochondrial dysfunction in atherosclerosis. Circ Res.

[52] Peng W, Cai G, Xia Y, Chen J, Wu P, Wang Z, Li G, Wei D (2019). Mitochondrial dysfunction in atherosclerosis. DNA Cell Biol.

[53] Yu EPK, Reinhold J, Yu H, Starks L, Uryga AK, Foote K, Finigan A, Figg N, Pung YF, Logan A (2017). Mitochondrial respiration is reduced in atherosclerosis, promoting necrotic core formation and reducing relative fibrous cap thickness. Arterioscler Thromb Vasc Biol.

[54] Flatow J, Buckley P, Miller BJ (2013). Meta-analysis of oxidative stress in schizophrenia. Biol Psychiatry.

[55] Liu T, Zhong S, Liao X, Chen J, He T, Lai S, Jia Y (2015). A meta-analysis of oxidative stress markers in depression. PLoS One.

[56] Morris G, Puri BK, Walker AJ, Maes M, Carvalho AF, Walder K, Berk M. Shared pathways for neuroprogression and somatoprogression in neuropsychiatric disorders. Neurosci Biobehav Rev. 2019;107:862–882. 10.1016/j.neubiorev.2019.09.025 31545987

[57] Morris G, Stubbs B, Kohler CA, Walder K, Slyepchenko A, Berk M, Carvalho AF (2018). The putative role of oxidative stress and inflammation in the pathophysiology of sleep dysfunction across neuropsychiatric disorders: focus on chronic fatigue syndrome, bipolar disorder and multiple sclerosis. Sleep Med Rev.

[58] Morris G, Walder K, McGee SL, Dean OM, Tye SJ, Maes M, Berk M (2017). A model of the mitochondrial basis of bipolar disorder. Neurosci Biobehav Rev.

[59] Pei L, Wallace DC (2018). Mitochondrial etiology of neuropsychiatric disorders. Biol Psychiatry.

[60] Morris G, Berk M (2015). The many roads to mitochondrial dysfunction in neuroimmune and neuropsychiatric disorders. BMC Med.

[61] Callaly E, Walder K, Morris G, Maes M, Debnath M, Berk M (2015). Mitochondrial dysfunction in the pathophysiology of bipolar disorder: effects of pharmacotherapy. Mini Rev Medi Chemi.

[62] Szewczyk A, Jarmuszkiewicz W, Koziel A, Sobieraj I, Nobik W, Lukasiak A, Skup A, Bednarczyk P, Drabarek B, Dymkowska D (2015). Mitochondrial mechanisms of endothelial dysfunction. Pharmacol Rep.

[63] Tang X, Luo Y-X, Chen H-Z, Liu D-P (2014). Mitochondria, endothelial cell function, and vascular diseases. Front Physiol.

[64] Kluge MA, Fetterman JL, Vita JA (2013). Mitochondria and endothelial function. Circ Res.

[65] Incalza MA, D'Oria R, Natalicchio A, Perrini S, Laviola L, Giorgino F (2018). Oxidative stress and reactive oxygen species in endothelial dysfunction associated with cardiovascular and metabolic diseases. Vasc Pharmacol.

[66] Haybar H, Shahrabi S, Rezaeeyan H, Shirzad R, Saki N (2019). Endothelial cells: from dysfunction mechanism to pharmacological effect in cardiovascular disease. Cardiovasc Toxicol.

[67] Szmitko PE, Wang C-H, Weisel RD, JRd A, Anderson TJ, Verma S (2003). New markers of inflammation and endothelial cell activation. Circulation.

[68] Teixeira BC, Lopes AL, Macedo RCO, Correa CS, Ramis TR, Ribeiro JL, Reischak-Oliveira A (2014). Inflammatory markers, endothelial function and cardiovascular risk. J Vasc Brasileiro.

[69] Gimbrone MA, García-Cardeña G (2016). Endothelial cell dysfunction and the pathobiology of atherosclerosis. Circ Res.

[70] Theodorou K, Boon RA (2018). Endothelial cell metabolism in atherosclerosis. Front Cell Dev Biol.

[71] Khan SY, Awad EM, Oszwald A, Mayr M, Yin X, Waltenberger B, Stuppner H, Lipovac M, Uhrin P, Breuss JM (2017). Premature senescence of endothelial cells upon chronic exposure to TNFα can be prevented by N-acetyl cysteine and plumericin. Sci Rep.

[72] Widlansky ME, Gokce N, Keaney JF, Vita JA (2003). The clinical implications of endothelial dysfunction. J Am Coll Cardiol.

[73] Childs BG, Li H, van Deursen JM (2018). Senescent cells: a therapeutic target for cardiovascular disease. J Clin Invest.

[74] Yeboah J, Folsom AR, Burke GL, Johnson C, Polak JF, Post W, Lima JA, Crouse JR, Herrington DM (2009). Predictive value of brachial flow-mediated dilation for incident cardiovascular events in a population-based study: the multi-ethnic study of atherosclerosis. Circulation.

[75] Rubinshtein R, Kuvin JT, Soffler M, Lennon RJ, Lavi S, Nelson RE, Pumper GM, Lerman LO, Lerman A (2010). Assessment of endothelial function by non-invasive peripheral arterial tonometry predicts late cardiovascular adverse events. Eur Heart J.

[76] Widmer RJ, Lerman A (2014). Endothelial dysfunction and cardiovascular disease. Glob Cardiol Sci Pract.

[77] Shechter M, Shechter A, Koren-Morag N, Feinberg MS, Hiersch L (2014). Usefulness of brachial artery flow-mediated dilation to predict long-term cardiovascular events in subjects without heart disease. Am J Cardiol.

[78] Oral E, Halici Z, Cinar I, Ozcan E, Kutlu Z (2019). Evaluation of endothelial dysfunction in bipolar affective disorders: serum endocan and urotensin-II levels. Clin Psychopharmacol Neurosci.

[79] Goldstein BI, Young LT (2013). Toward clinically applicable biomarkers in bipolar disorder: focus on BDNF, inflammatory markers, and endothelial function. Curr Psychiatry Rep.

[80] Vetter MW, Martin B-J, Fung M, Pajevic M, Anderson TJ, Raedler TJ (2015). Microvascular dysfunction in schizophrenia: a case–control study. NPJ Schizophr.

[81] Nguyen TT, Dev SI, Chen G, Liou SC, Martin AS, Irwin MR, Carroll JE, Tu X, Jeste DV, Eyler LT (2018). Abnormal levels of vascular endothelial biomarkers in schizophrenia. Eur Arch Psychiatry Clin Neurosci.

[82] Rybakowski JK, Wykretowicz A, Heymann-Szlachcinska A, Wysocki H (2006). Impairment of endothelial function in unipolar and bipolar depression. Biol Psychiatry.

[83] Tsoupras A, Lordan R, Zabetakis I (2018). Inflammation, not cholesterol, is a cause of chronic disease. Nutrients.

[84] Higgins P, Dawson J, Lees KR, McArthur K, Quinn TJ, Walters MR (2012). Xanthine oxidase inhibition for the treatment of cardiovascular disease: a systematic review and meta-analysis. Cardiovasc Ther.

[85] Nussbaum C, Klinke A, Adam M, Baldus S, Sperandio M (2013). Myeloperoxidase: a leukocyte-derived protagonist of inflammation and cardiovascular disease. Antioxid Redox Signal.

[86] Rajendran P, Rengarajan T, Thangavel J, Nishigaki Y, Sakthisekaran D, Sethi G, Nishigaki I (2013). The vascular endothelium and human diseases. Int J Biol Sci.

[87] Fledderus JO, Boon RA, Volger OL, Hurttila H, Yla-Herttuala S, Pannekoek H, Levonen AL, Horrevoets AJ (2008). KLF2 primes the antioxidant transcription factor Nrf2 for activation in endothelial cells. Arterioscler Thromb Vasc Biol.

[88] Nayak L, Lin Z, Jain MK (2011). “Go with the flow”: how Krüppel-like factor 2 regulates the vasoprotective effects of shear stress. Antioxid Redox Signal.

[89] Chiplunkar AR, Curtis BC, Eades GL, Kane MS, Fox SJ, Haar JL, Lloyd JA (2013). The Kruppel-like factor 2 and Kruppel-like factor 4 genes interact to maintain endothelial integrity in mouse embryonic vasculogenesis. BMC Dev Biol.

[90] Young A, Wu W, Sun W, Benjamin Larman H, Wang N, Li YS, Shyy JY, Chien S, Garcia-Cardena G (2009). Flow activation of AMP-activated protein kinase in vascular endothelium leads to Kruppel-like factor 2 expression. Arterioscler Thromb Vasc Biol.

[91] Zhong F, Lee K, He JC (2018). Role of Krüppel-like factor-2 in kidney disease. Nephrology.

[92] Doddaballapur A, Michalik KM, Manavski Y, Lucas T, Houtkooper RH, You X, Chen W, Zeiher AM, Potente M, Dimmeler S (2015). Laminar shear stress inhibits endothelial cell metabolism via KLF2-mediated repression of PFKFB3. Arterioscler Thromb Vasc Biol.

[93] Harding IC, Mitra R, Mensah SA, Nersesyan A, Bal NN, Ebong EE (2019). Endothelial barrier reinforcement relies on flow-regulated glycocalyx, a potential therapeutic target. Biorheology.

[94] Fukai T, Ushio-Fukai M (2011). Superoxide dismutases: role in redox signaling, vascular function, and diseases. Antioxid Redox Signal.

[95] Ganguli A, Persson L, Palmer IR, Evans I, Yang L, Smallwood R, Black R, Qwarnstrom EE (2005). Distinct NF-kappaB regulation by shear stress through Ras-dependent IkappaBalpha oscillations: real-time analysis of flow-mediated activation in live cells. Circ Res.

[96] Mohan S, Koyoma K, Thangasamy A, Nakano H, Glickman RD, Mohan N (2007). Low shear stress preferentially enhances IKK activity through selective sources of ROS for persistent activation of NF-kappaB in endothelial cells. Am J Physiol Cell Physiol.

[97] Kadohama T, Akasaka N, Nishimura K, Hoshino Y, Sasajima T, Sumpio BE (2006). p38 Mitogen-activated protein kinase activation in endothelial cell is implicated in cell alignment and elongation induced by fluid shear stress. Endothelium.

[98] Cuhlmann S, Van der Heiden K, Saliba D, Tremoleda JL, Khalil M, Zakkar M, Chaudhury H, Luong le A, Mason JC, Udalova I (2011). Disturbed blood flow induces RelA expression via c-Jun N-terminal kinase 1: a novel mode of NF-kappaB regulation that promotes arterial inflammation. Circ Res.

[99] Amaya R, Pierides A, Tarbell JM (2015). The interaction between fluid wall shear stress and solid circumferential strain affects endothelial gene expression. PLoS One.

[100] Baeriswyl DC, Prionisti I, Peach T, Tsolkas G, Chooi KY, Vardakis J, Morel S, Diagbouga MR, Bijlenga P, Cuhlmann S (2019). Disturbed flow induces a sustained, stochastic NF-κB activation which may support intracranial aneurysm growth in vivo. Sci Rep.

[101] Pan L, Hong Z, Yu L, Gao Y, Zhang R, Feng H, Su L, Wang G (2017). Shear stress induces human aortic endothelial cell apoptosis via interleukin1 receptorassociated kinase 2induced endoplasmic reticulum stress. Mol Med Rep.

[102] Bailey KA, Haj FG, Simon SI, Passerini AG (2017). Atherosusceptible shear stress activates endoplasmic reticulum stress to promote endothelial inflammation. Sci Rep.

[103] Maamoun H, Abdelsalam SS, Zeidan A, Korashy HM, Agouni A (2019). Endoplasmic reticulum stress: a critical molecular driver of endothelial dysfunction and cardiovascular disturbances associated with diabetes. Int J Mol Sci.

[104] Lenna S, Han R, Trojanowska M (2014). Endoplasmic reticulum stress and endothelial dysfunction. IUBMB Life.

[105] Warboys CM, de Luca A, Amini N, Luong L, Duckles H, Hsiao S, White A, Biswas S, Khamis R, Chong CK (2014). Disturbed flow promotes endothelial senescence via a p53-dependent pathway. Arterioscler Thromb Vasc Biol.

[106] Vion A-C, Kheloufi M, Hammoutene A, Poisson J, Lasselin J, Devue C, Pic I, Dupont N, Busse J, Stark K (2017). Autophagy is required for endothelial cell alignment and atheroprotection under physiological blood flow. Proc Natl Acad Sci.

[107] Katsuumi G, Shimizu I, Yoshida Y, Minamino T: Vascular senescence in cardiovascular and metabolic diseases. Front Cardiovas Med 2018, 5(18). 10.3389/fcvm.2018.00018 PMC5845435 29556500

[108] Tabas I (2010). The role of endoplasmic reticulum stress in the progression of atherosclerosis. Circ Res.

[109] Moore KJ, Sheedy FJ, Fisher EA (2013). Macrophages in atherosclerosis: a dynamic balance. Nat Rev Immunol.

[110] Mitroulis I, Alexaki VI, Kourtzelis I, Ziogas A, Hajishengallis G, Chavakis T (2015). Leukocyte integrins: role in leukocyte recruitment and as therapeutic targets in inflammatory disease. Pharmacol Ther.

[111] Čejková S, Králová-Lesná I, Poledne R (2016). Monocyte adhesion to the endothelium is an initial stage of atherosclerosis development. Cor et Vasa.

[112] Ramji DP, Davies TS (2015). Cytokines in atherosclerosis: key players in all stages of disease and promising therapeutic targets. Cytokine Growth Factor Rev.

[113] Yu XH, Fu YC, Zhang DW, Yin K, Tang CK (2013). Foam cells in atherosclerosis. Clin Chim Acta.

[114] Chistiakov DA, Melnichenko AA, Myasoedova VA, Grechko AV, Orekhov AN (2017). Mechanisms of foam cell formation in atherosclerosis. J Mol Med.

[115] Chistiakov DA, Bobryshev YV, Orekhov AN (2016). Macrophage-mediated cholesterol handling in atherosclerosis. J Cell Mol Med.

[116] Fang Y, Wu D, Birukov KG (2019). Mechanosensing and mechanoregulation of endothelial cell functions. Compr Physiol.

[117] Conway DE, Breckenridge MT, Hinde E, Gratton E, Chen CS, Schwartz MA (2013). Fluid shear stress on endothelial cells modulates mechanical tension across VE-cadherin and PECAM-1. Curr Biol.

[118] Coon BG, Baeyens N, Han J, Budatha M, Ross TD, Fang JS, Yun S, Thomas JL, Schwartz MA (2015). Intramembrane binding of VE-cadherin to VEGFR2 and VEGFR3 assembles the endothelial mechanosensory complex. J Cell Biol.

[119] Conway DE, Schwartz MA (2015). Mechanotransduction of shear stress occurs through changes in VE-cadherin and PECAM-1 tension: implications for cell migration. Cell Adhes Migr.

[120] Dejana E, Vestweber D (2013). The role of VE-cadherin in vascular morphogenesis and permeability control. Prog Mol Biol Transl Sci.

[121] Conway DE, Coon BG, Budatha M, Arsenovic PT, Orsenigo F, Wessel F, Zhang J, Zhuang Z, Dejana E, Vestweber D (2017). VE-cadherin phosphorylation regulates endothelial fluid shear stress responses through the polarity protein LGN. Curr Biol.

[122] Sidibé A, Imhof BA (2014). VE-cadherin phosphorylation decides: vascular permeability or diapedesis. Nat Immunol.

[123] Kroon J, Heemskerk N, Kalsbeek MJT, de Waard V, van Rijssel J, van Buul JD (2017). Flow-induced endothelial cell alignment requires the RhoGEF Trio as a scaffold protein to polarize active Rac1 distribution. Mol Biol Cell.

[124] Timmerman I, Heemskerk N, Kroon J, Schaefer A, van Rijssel J, Hoogenboezem M, van Unen J, Goedhart J, Gadella TW, Yin T (2015). A local VE-cadherin and Trio-based signaling complex stabilizes endothelial junctions through Rac1. J Cell Sci.

[125] Rho SS, Ando K, Fukuhara S (2017). Dynamic regulation of vascular permeability by vascular endothelial cadherin-mediated endothelial cell-cell junctions. J Nippon Med School.

[126] Mandyam CD, Villalpando EG, Steiner NL, Quach LW, Fannon MJ, Somkuwar SS (2017). Platelet endothelial cell adhesion molecule-1 and oligodendrogenesis: significance in alcohol use disorders. Brain Sci.

[127] Feng YM, Chen XH, Zhang X (2016). Roles of PECAM-1 in cell function and disease progression. Eur Rev Med Pharmacol Sci.

[128] Park S, Sorenson CM, Sheibani N (2015). PECAM-1 isoforms, eNOS and endoglin axis in regulation of angiogenesis. Clin Sci.

[129] Lertkiatmongkol P, Paddock C, Newman DK, Zhu J, Thomas MJ, Newman PJ (2016). The role of sialylated glycans in human platelet endothelial cell adhesion molecule 1 (PECAM-1)-mediated trans homophilic interactions and endothelial cell barrier function. J Biol Chem.

[130] Stevens HY, Melchior B, Bell KS, Yun S, Yeh J-C, Frangos JA (2008). PECAM-1 is a critical mediator of atherosclerosis. Dis Models Mech.

[131] Feaver RE, Gelfand BD, Blackman BR (2013). Human haemodynamic frequency harmonics regulate the inflammatory phenotype of vascular endothelial cells. Nat Commun.

[132] Chistiakov DA, Orekhov AN, Bobryshev YV (2016). Endothelial PECAM-1 and its function in vascular physiology and atherogenic pathology. Exp Mol Pathol.

[133] Di Lorenzo A, Lin MI, Murata T, Landskroner-Eiger S, Schleicher M, Kothiya M, Iwakiri Y, Yu J, Huang PL, Sessa WC (2013). eNOS-derived nitric oxide regulates endothelial barrier function through VE-cadherin and Rho GTPases. J Cell Sci.

[134] Zhuang Y, Peng H, Mastej V, Chen W (2016). MicroRNA regulation of endothelial junction proteins and clinical consequence. Mediat Inflamm.

[135] Fernández-Hernando C, Suárez Y (2018). MicroRNAs in endothelial cell homeostasis and vascular disease. Curr Opin Hematol.

[136] Sun X, Belkin N, Feinberg MW (2013). Endothelial microRNAs and atherosclerosis. Curr Atheroscler Rep.

[137] Kumar S, Kim CW, Simmons RD, Jo H (2014). Role of flow-sensitive microRNAs in endothelial dysfunction and atherosclerosis: mechanosensitive athero-miRs. Arterioscler Thromb Vasc Biol.

[138] Kumar S, Williams D, Sur S, Wang JY, Jo H (2019). Role of flow-sensitive microRNAs and long noncoding RNAs in vascular dysfunction and atherosclerosis. Vasc Pharmacol.

[139] Marin T, Gongol B, Chen Z, Woo B, Subramaniam S, Chien S, Shyy JY (2013). Mechanosensitive microRNAs-role in endothelial responses to shear stress and redox state. Free Radic Biol Med.

[140] Feinberg MW, Moore KJ (2016). MicroRNA regulation of atherosclerosis. Circ Res.

[141] Gou L, Zhao L, Song W, Wang L, Liu J, Zhang H, Huang Y, Lau CW, Yao X, Tian XY (2018). Inhibition of miR-92a suppresses oxidative stress and improves endothelial function by upregulating heme oxygenase-1 in db/db mice. Antioxid Redox Signal.

[142] Loyer X, Potteaux S, Vion AC, Guerin CL, Boulkroun S, Rautou PE, Ramkhelawon B, Esposito B, Dalloz M, Paul JL (2014). Inhibition of microRNA-92a prevents endothelial dysfunction and atherosclerosis in mice. Circ Res.

[143] Fang Y, Davies PF (2012). Site-specific microRNA-92a regulation of Kruppel-like factors 4 and 2 in atherosusceptible endothelium. Arterioscler Thromb Vasc Biol.

[144] Wu W, Xiao H, Laguna-Fernandez A, Villarreal G, Wang KC, Geary GG, Zhang Y, Wang WC, Huang HD, Zhou J (2011). Flow-dependent regulation of Kruppel-like factor 2 is mediated by microRNA-92a. Circulation.

[145] Qin X, Wang X, Wang Y, Tang Z, Cui Q, Xi J, Li YS, Chien S, Wang N (2010). MicroRNA-19a mediates the suppressive effect of laminar flow on cyclin D1 expression in human umbilical vein endothelial cells. Proc Natl Acad Sci U S A.

[146] Zhong L, Simard MJ, Huot J (2018). Endothelial microRNAs regulating the NF-kappaB pathway and cell adhesion molecules during inflammation. FASEB J.

[147] SenBanerjee S, Lin Z, Atkins GB, Greif DM, Rao RM, Kumar A, Feinberg MW, Chen Z, Simon DI, Luscinskas FW (2004). KLF2 is a novel transcriptional regulator of endothelial proinflammatory activation. J Exp Med.

[148] Zhou G, Hamik A, Nayak L, Tian H, Shi H, Lu Y, Sharma N, Liao X, Hale A, Boerboom L (2012). Endothelial Kruppel-like factor 4 protects against atherothrombosis in mice. J Clin Invest.

[149] Taganov KD, Boldin MP, Chang KJ, Baltimore D (2006). NF-kappaB-dependent induction of microRNA miR-146, an inhibitor targeted to signaling proteins of innate immune responses. Proc Natl Acad Sci U S A.

[150] Yamakuchi M, Hashiguchi T (2018). Endothelial cell aging: how miRNAs contribute?. J Clin Med.

[151] Bartoszewski R, Serocki M, Janaszak-Jasiecka A, Bartoszewska S, Kochan-Jamrozy K, Piotrowski A, Króliczewski J, Collawn JF (2017). miR-200b downregulates Kruppel Like Factor 2 (KLF2) during acute hypoxia in human endothelial cells. Eur J Cell Biol.

[152] Neth P, Nazari-Jahantigh M, Schober A, Weber C (2013). MicroRNAs in flow-dependent vascular remodelling. Cardiovasc Res.

[153] Pan Y, Hui X, Hoo RLC, Ye D, Chan CYC, Feng T, Wang Y, Lam KSL, Xu A (2019). Adipocyte-secreted exosomal microRNA-34a inhibits M2 macrophage polarization to promote obesity-induced adipose inflammation. J Clin Invest.

[154] Chen Q, Li L, Tu Y, Zheng LL, Liu W, Zuo XY, He YM, Zhang SY, Zhu W, Cao JP (2014). MiR-34a regulates apoptosis in liver cells by targeting the KLF4 gene. Cell Mol Biol Lett.

[155] Pircher J, Merkle M, Wörnle M, Ribeiro A, Czermak T, Stampnik Y, Mannell H, Niemeyer M, Vielhauer V, Krötz F (2012). Prothrombotic effects of tumor necrosis factor alpha in vivo are amplified by the absence of TNF-alpha receptor subtype 1 and require TNF-alpha receptor subtype 2. Arthritis Res Ther.

[156] Pignatelli P, De Biase L, Lenti L, Tocci G, Brunelli A, Cangemi R, Riondino S, Grego S, Volpe M, Violi F (2005). Tumor necrosis factor-alpha as trigger of platelet activation in patients with heart failure. Blood.

[157] Jang JY, Min JH, Chae YH, Baek JY, Wang SB, Park SJ, Oh GT, Lee S-H, Ho Y-S, Chang T-S (2014). Reactive oxygen species play a critical role in collagen-induced platelet activation via SHP-2 oxidation. Antioxid Redox Signal.

[158] Qiao J, Arthur JF, Gardiner EE, Andrews RK, Zeng L, Xu K (2018). Regulation of platelet activation and thrombus formation by reactive oxygen species. Redox Biol.

[159] Smith TL, Weyrich AS (2011). Platelets as central mediators of systemic inflammatory responses. Thromb Res.

[160] Jenne CN, Urrutia R, Kubes P (2013). Platelets: bridging hemostasis, inflammation, and immunity. Int J Lab Hematol.

[161] Freedman JE (2008). Oxidative stress and platelets. Arterioscler Thromb Vasc Biol.

[162] Violi F, Pignatelli P, Basili S (2010). Nutrition, supplements, and vitamins in platelet function and bleeding. Circulation.

[163] El Haouari M (2019). Platelet oxidative stress and its relationship with cardiovascular diseases in type 2 diabetes mellitus patients. Curr Med Chem.

[164] Hamilos M, Petousis S, Parthenakis F (2018). Interaction between platelets and endothelium: from pathophysiology to new therapeutic options. Cardiovasc Diagn Ther.

[165] Celik S, Langer H, Stellos K, May AE, Shankar V, Kurz K, Katus HA, Gawaz MP, Dengler TJ (2007). Platelet-associated LIGHT (TNFSF14) mediates adhesion of platelets to human vascular endothelium. Thromb Haemost.

[166] Celik S, Shankar V, Richter A, Hippe HJ, Akhavanpoor M, Bea F, Erbel C, Urban S, Blank N, Wambsganss N (2009). Proinflammatory and prothrombotic effects on human vascular endothelial cells of immune-cell-derived LIGHT. Eur J Med Res.

[167] Otterdal K, Smith C, Oie E, Pedersen TM, Yndestad A, Stang E, Endresen K, Solum NO, Aukrust P, Damås JK (2006). Platelet-derived LIGHT induces inflammatory responses in endothelial cells and monocytes. Blood.

[168] Gerdes N, Seijkens T, Lievens D, Kuijpers MJE, Winkels H, Projahn D, Hartwig H, Beckers L, Megens RTA, Boon L (2016). Platelet CD40 exacerbates atherosclerosis by transcellular activation of endothelial cells and leukocytes. Arterioscler Thromb Vasc Biol.

[169] Michel NA, Zirlik A, Wolf D: CD40L and its receptors in atherothrombosis—an update. Front Cardiovas Med 2017, 4(40). 10.3389/fcvm.2017.00040 PMC5477003 28676852

[170] Heiss EH, Schachner D, Zimmermann K, Dirsch VM (2013). Glucose availability is a decisive factor for Nrf2-mediated gene expression. Redox Biol.

[171] Deshauer C, Morgan AM, Ryan EO, Handel TM, Prestegard JH, Wang X (2015). Interactions of the chemokine CCL5/RANTES with medium-sized chondroitin sulfate ligands. Structure.

[172] von Hundelshausen P, Schmitt MMN: Platelets and their chemokines in atherosclerosis—clinical applications. Front Physiol 2014, 5(294). 10.3389/fphys.2014.00294 PMC4126210 25152735

[173] Jones KL, Maguire JJ, Davenport AP (2011). Chemokine receptor CCR5: from AIDS to atherosclerosis. Br J Pharmacol.

[174] Øynebråten I, Barois N, Bergeland T, Küchler AM, Bakke O, Haraldsen G (2015). Oligomerized, filamentous surface presentation of RANTES/CCL5 on vascular endothelial cells. Sci Rep.

[175] Sonmez O, Sonmez M (2017). Role of platelets in immune system and inflammation. Porto Biomed J.

[176] Deppermann C, Kubes P (2018). Start a fire, kill the bug: the role of platelets in inflammation and infection. Innate Immun.

[177] Carnevale R, Bartimoccia S, Nocella C, Di Santo S, Loffredo L, Illuminati G, Lombardi E, Boz V, Del Ben M, De Marco L (2014). LDL oxidation by platelets propagates platelet activation via an oxidative stress-mediated mechanism. Atherosclerosis.

[178] Carnevale R, Pignatelli P, Lenti L, Buchetti B, Sanguigni V, Di Santo S, Violi F (2007). LDL are oxidatively modified by platelets via GP91(phox) and accumulate in human monocytes. FASEB J.

[179] Sachais BS, Kuo A, Nassar T, Morgan J, Kariko K, Williams KJ, Feldman M, Aviram M, Shah N, Jarett L (2002). Platelet factor 4 binds to low-density lipoprotein receptors and disrupts the endocytic machinery, resulting in retention of low-density lipoprotein on the cell surface. Blood.

[180] Nassar T, Sachais BS, Se A, Kowalska MA, Bdeir K, Leitersdorf E, Hiss E, Ziporen L, Aviram M, Cines D (2003). Platelet factor 4 enhances the binding of oxidized low-density lipoprotein to vascular wall cells. J Biol Chem.

[181] Li X, Meng X, Gao X, Pang X, Wang Y, Wu X, Deng X, Zhang Q, Sun C, Li Y (2018). Elevated serum xanthine oxidase activity is associated with the development of type 2 diabetes: a prospective cohort study. Diabetes Care.

[182] Battelli MG, Bortolotti M, Polito L, Bolognesi A (2018). The role of xanthine oxidoreductase and uric acid in metabolic syndrome. Biochim Biophys Acta Mol basis Dis.

[183] Battelli MG, Bolognesi A, Polito L (2014). Pathophysiology of circulating xanthine oxidoreductase: new emerging roles for a multi-tasking enzyme. Biochim Biophys Acta (BBA) - Mol Basis Dis.

[184] Schuchardt M, Herrmann J, Tolle M, van der Giet M (2017). Xanthine oxidase and its role as target in cardiovascular disease: cardiovascular protection by enzyme inhibition?. Curr Pharm Des.

[185] Alem MM (2018). Allopurinol and endothelial function: a systematic review with meta-analysis of randomized controlled trials. Cardiovasc Ther.

[186] Xin W, Mi S, Lin Z (2016). Allopurinol therapy improves vascular endothelial function in subjects at risk for cardiovascular diseases: a meta-analysis of randomized controlled trials. Cardiovasc Ther.

[187] Cicero AFG, Pirro M, Watts GF, Mikhailidis DP, Banach M, Sahebkar A (2018). Effects of allopurinol on endothelial function: a systematic review and meta-analysis of randomized placebo-controlled trials. Drugs.

[188] Bredemeier M, Lopes LM, Eisenreich MA, Hickmann S, Bongiorno GK, d’Avila R, Morsch ALB, da Silva Stein F, Campos GGD (2018). Xanthine oxidase inhibitors for prevention of cardiovascular events: a systematic review and meta-analysis of randomized controlled trials. BMC Cardiovasc Disord.

[189] Munzel T, Camici GG, Maack C, Bonetti NR, Fuster V, Kovacic JC (2017). Impact of oxidative stress on the heart and vasculature: part 2 of a 3-part series. J Am Coll Cardiol.

[190] George J. Role of urate, xanthine oxidase and the effects of allopurinol in vascular oxidative stress. Vasc Health Risk Manag. 2009:265. 10.2147/vhrm.s4265 PMC2672460 19436671

[191] Gao X, Zhang H, Belmadani S, Wu J, Xu X, Elford H, Potter BJ, Zhang C (2008). Role of TNF-alpha-induced reactive oxygen species in endothelial dysfunction during reperfusion injury. Am J Phys Heart Circ Phys.

[192] Zhang C, Hein TW, Wang W, Ren Y, Shipley RD, Kuo L (2006). Activation of JNK and xanthine oxidase by TNF-alpha impairs nitric oxide-mediated dilation of coronary arterioles. J Mol Cell Cardiol.

[193] Ogura J, Kuwayama K, Sasaki S, Kaneko C, Koizumi T, Yabe K, Tsujimoto T, Takeno R, Takaya A, Kobayashi M (2015). Reactive oxygen species derived from xanthine oxidase interrupt dimerization of breast cancer resistance protein, resulting in suppression of uric acid excretion to the intestinal lumen. Biochem Pharmacol.

[194] Ives A, Nomura J, Martinon F, Roger T, LeRoy D, Miner JN, Simon G, Busso N, So A (2015). Xanthine oxidoreductase regulates macrophage IL1β secretion upon NLRP3 inflammasome activation. Nat Commun.

[195] Maruhashi T, Hisatome I, Kihara Y, Higashi Y (2018). Hyperuricemia and endothelial function: from molecular background to clinical perspectives. Atherosclerosis.

[196] Desco M-C, Asensi M, Márquez R, Martínez-Valls J, Vento M, Pallardó FV, Sastre J, Viña J (2002). Xanthine oxidase is involved in free radical production in type 1 diabetes. Protect Allopurinol.

[197] Landmesser U, Spiekermann S, Preuss C, Sorrentino S, Fischer D, Manes C, Mueller M, Drexler H (2007). Angiotensin II induces endothelial xanthine oxidase activation. Arterioscler Thromb Vasc Biol.

[198] Houston M, Estevez A, Chumley P, Aslan M, Marklund S, Parks DA, Freeman BA (1999). Binding of xanthine oxidase to vascular endothelium: kinetic characterization and oxidative impairment of nitric oxide-dependent signaling. J Biol Chem.

[199] Tomiyama H, Higashi Y, Takase B, Node K, Sata M, Inoue T, Ishibashi Y, Ueda S, Shimada K, Yamashina A (2011). Relationships among hyperuricemia, metabolic syndrome, and endothelial function. Am J Hypertens.

[200] Cicero AFG, Rosticci M, Parini A, Baronio C, D'Addato S, Borghi C (2014). Serum uric acid is inversely proportional to estimated stroke volume and cardiac output in a large sample of pharmacologically untreated subjects: data from the Brisighella Heart Study. Intern Emerg Med.

[201] Volterrani M, Iellamo F, Sposato B, Romeo F (2016). Uric acid lowering therapy in cardiovascular diseases. Int J Cardiol.

[202] Ndrepepa G (2018). Uric acid and cardiovascular disease. Clin Chim Acta.

[203] Muiesan ML, Agabiti-Rosei C, Paini A, Salvetti M (2016). Uric acid and cardiovascular disease: an update. Eur Cardiol.

[204] Rahimi-Sakak F, Maroofi M, Rahmani J, Bellissimo N, Hekmatdoost A (2019). Serum uric acid and risk of cardiovascular mortality: a systematic review and dose-response meta-analysis of cohort studies of over a million participants. BMC Cardiovasc Disord.

[205] Chang C-C, Wu C-H, Liu L-K, Chou R-H, Kuo C-S, Huang P-H, Chen L-K, Lin S-J (2018). Association between serum uric acid and cardiovascular risk in nonhypertensive and nondiabetic individuals: the Taiwan I-Lan Longitudinal Aging Study. Sci Rep.

[206] Kang DH, Han L, Ouyang X, Kahn AM, Kanellis J, Li P, Feng L, Nakagawa T, Watanabe S, Hosoyamada M (2005). Uric acid causes vascular smooth muscle cell proliferation by entering cells via a functional urate transporter. Am J Nephrol.

[207] Price KL, Sautin YY, Long DA (2006). Human vascular smooth muscle cells express a urate transporter. J Am Soc Nephrol.

[208] Liu S, Yuan Y, Zhou Y, Zhao M, Chen Y, Cheng J, Lu Y, Liu J (2017). Phloretin attenuates hyperuricemia-induced endothelial dysfunction through co-inhibiting inflammation and GLUT9-mediated uric acid uptake. J Cell Mol Med.

[209] Xu L, Shi Y, Zhuang S, Liu N (2017). Recent advances on uric acid transporters. Oncotarget.

[210] Zhen H, Gui F (2017). The role of hyperuricemia on vascular endothelium dysfunction. Biomed Rep.

[211] Cai W, Duan X-M, Liu Y, Yu J, Tang Y-L, Liu Z-L, Jiang S, Zhang C-P, Liu J-Y, Xu J-X (2017). Uric acid induces endothelial dysfunction by activating the HMGB1/RAGE signaling pathway. Biomed Res Int.

[212] Yang X, Gu J, Lv H, Li H, Cheng Y, Liu Y, Jiang Y (2019). Uric acid induced inflammatory responses in endothelial cells via up-regulating(pro)renin receptor. Biomed Pharmacother.

[213] Liang WY, Zhu XY, Zhang JW, Feng XR, Wang YC, Liu ML (2015). Uric acid promotes chemokine and adhesion molecule production in vascular endothelium via nuclear factor-kappa B signaling. Nutr Metabol Cardiovasc Dis.

[214] Rabadi MM, Kuo M-C, Ghaly T, Rabadi SM, Weber M, Goligorsky MS, Ratliff BB (2012). Interaction between uric acid and HMGB1 translocation and release from endothelial cells. Am J Physiol Renal Physiol.

[215] Braga TT, Forni MF, Correa-Costa M, Ramos RN, Barbuto JA, Branco P, Castoldi A, Hiyane MI, Davanso MR, Latz E (2017). Soluble uric acid activates the NLRP3 inflammasome. Sci Rep.

[216] Shi Y, Evans JE, Rock KL (2003). Molecular identification of a danger signal that alerts the immune system to dying cells. Nature.

[217] Park J-H, Jin YM, Hwang S, Cho D-H, Kang D-H, Jo I (2013). Uric acid attenuates nitric oxide production by decreasing the interaction between endothelial nitric oxide synthase and calmodulin in human umbilical vein endothelial cells: a mechanism for uric acid-induced cardiovascular disease development. Nitric oxide.

[218] Verzola D, Ratto E, Villaggio B, Parodi EL, Pontremoli R, Garibotto G, Viazzi F (2014). Uric acid promotes apoptosis in human proximal tubule cells by oxidative stress and the activation of NADPH oxidase NOX 4. PLoS One.

[219] K-s L, Z-h P, Cheng W-j, C-f D, Tong H (2016). Endoplasmic reticulum stress-induced apoptosis in the development of reproduction. J Reprod Contracept.

[220] Komori H, Yamada K, Tamai I (2018). Hyperuricemia enhances intracellular urate accumulation via down-regulation of cell-surface BCRP/ABCG2 expression in vascular endothelial cells. Biochim Biophys Acta Biomembr.

[221] Kong J, Chalcraft K, Mandur TS, Jimenez-Saiz R, Walker TD, Goncharova S, Gordon ME, Naji L, Flader K, Larche M (2015). Comprehensive metabolomics identifies the alarmin uric acid as a critical signal for the induction of peanut allergy. Allergy.

[222] Kanda A, Ishida S (2019). (Pro)renin receptor: involvement in diabetic retinopathy and development of molecular targeted therapy. J Diab Investig.

[223] Yu M-A, Sánchez-Lozada LG, Johnson RJ, Kang D-H (2010). Oxidative stress with an activation of the renin–angiotensin system in human vascular endothelial cells as a novel mechanism of uric acid-induced endothelial dysfunction. J Hypertens.

[224] Corry DB, Eslami P, Yamamoto K, Nyby MD, Makino H, Tuck ML (2008). Uric acid stimulates vascular smooth muscle cell proliferation and oxidative stress via the vascular renin–angiotensin system. J Hypertens.

[225] Uraoka M, Ikeda K, Nakagawa Y, Koide M, Akakabe Y, Nakano-Kurimoto R, Takahashi T, Matoba S, Yamada H, Okigaki M (2009). Prorenin induces ERK activation in endothelial cells to enhance neovascularization independently of the renin-angiotensin system. Biochem Biophys Res Commun.

[226] Ho E, Karimi Galougahi K, Liu C-C, Bhindi R, Figtree GA (2013). Biological markers of oxidative stress: applications to cardiovascular research and practice. Redox Biol.

[227] Khan AA, Alsahli MA, Rahmani AH (2018). Myeloperoxidase as an active disease biomarker: recent biochemical and pathological perspectives. Med Sci (Basel).

[228] Aratani Y (2018). Myeloperoxidase: its role for host defense, inflammation, and neutrophil function. Arch Biochem Biophys.

[229] Odobasic D, Kitching AR, Holdsworth SR (2016). Neutrophil-mediated regulation of innate and adaptive immunity: the role of myeloperoxidase. J Immunol Res.

[230] Delporte C, Van Antwerpen P, Vanhamme L, Roumeguere T, Zouaoui Boudjeltia K (2013). Low-density lipoprotein modified by myeloperoxidase in inflammatory pathways and clinical studies. Mediat Inflamm.

[231] Roumeguere T, Zouaoui Boudjeltia K, Babar S, Nuyens V, Rousseau A, Van Antwerpen P, Ducobu J, Wespes E, Vanhaeverbeek M (2010). Effects of phosphodiesterase inhibitors on the inflammatory response of endothelial cells stimulated by myeloperoxidase-modified low-density lipoprotein or tumor necrosis factor alpha. Eur Urol.

[232] Boudjeltia KZ, Legssyer I, Van Antwerpen P, Kisoka RL, Babar S, Moguilevsky N, Delree P, Ducobu J, Remacle C, Vanhaeverbeek M (2006). Triggering of inflammatory response by myeloperoxidase-oxidized LDL. Biochem Cell Biol.

[233] Sokolov AV, Kostevich VA, Runova OL, Gorudko IV, Vasilyev VB, Cherenkevich SN, Panasenko OM (2014). Proatherogenic modification of LDL by surface-bound myeloperoxidase. Chem Phys Lipids.

[234] Wang G, Qian P, Jackson FR, Qian G, Wu G (2008). Sequential activation of JAKs, STATs and xanthine dehydrogenase/oxidase by hypoxia in lung microvascular endothelial cells. Int J Biochem Cell Biol.

[235] Jerke U, Rolle S, Purfürst B, Luft FC, Nauseef WM, Kettritz R (2013). β2 integrin-mediated cell-cell contact transfers active myeloperoxidase from neutrophils to endothelial cells. J Biol Chem.

[236] Yiginer O, Ozcelik F, Inanc T, Aparci M, Ozmen N, Cingozbay BY, Kardesoglu E, Suleymanoglu S, Sener G, Cebeci BS (2008). Allopurinol improves endothelial function and reduces oxidant-inflammatory enzyme of myeloperoxidase in metabolic syndrome. Clin Res Cardiol.

[237] Stamp LK, Turner R, Khalilova IS, Zhang M, Drake J, Forbes LV, Kettle AJ (2014). Myeloperoxidase and oxidation of uric acid in gout: implications for the clinical consequences of hyperuricaemia. Rheumatology (Oxford).

[238] Astern JM, Pendergraft WF, Falk RJ, Jennette JC, Schmaier AH, Mahdi F, Preston GA (2007). Myeloperoxidase interacts with endothelial cell-surface cytokeratin 1 and modulates bradykinin production by the plasma Kallikrein-Kinin system. Am J Pathol.

[239] Baldus S, Heitzer T, Eiserich JP, Lau D, Mollnau H, Ortak M, Petri S, Goldmann B, Duchstein H-J, Berger J (2004). Myeloperoxidase enhances nitric oxide catabolism during myocardial ischemia and reperfusion. Free Radic Biol Med.

[240] Eiserich JP, Baldus S, Brennan M-L, Ma W, Zhang C, Tousson A, Castro L, Lusis AJ, Nauseef WM, White CR (2002). Myeloperoxidase, a leukocyte-derived vascular NO oxidase. Sci (New York).

[241] Maiocchi S, Dang L, Morris J, Thomas S, Rees M (2015). PP28 - inhibition of myeloperoxidase-mediated endothelial dysfunction by nitroxides. Free Radic Biol Med.

[242] Bienert GP, Moller AL, Kristiansen KA, Schulz A, Moller IM, Schjoerring JK, Jahn TP (2007). Specific aquaporins facilitate the diffusion of hydrogen peroxide across membranes. J Biol Chem.

[243] Al Ghouleh I, Frazziano G, Rodriguez AI, Csányi G, Maniar S, St Croix CM, Kelley EE, Egaña LA, Song GJ, Bisello A (2013). Aquaporin 1, Nox1, and Ask1 mediate oxidant-induced smooth muscle cell hypertrophy. Cardiovasc Res.

[244] Sies H (2017). Hydrogen peroxide as a central redox signaling molecule in physiological oxidative stress: oxidative eustress. Redox Biol.

[245] Morawietz H (2011). Endothelial NADPH oxidases: friends or foes?. Basic Res Cardiol.

[246] Oliveira-Marques V, Marinho HS, Cyrne L, Antunes F (2009). Role of hydrogen peroxide in NF-kappaB activation: from inducer to modulator. Antioxid Redox Signal.

[247] Brandes RP, Weissmann N, Schroder K (2014). Nox family NADPH oxidases: molecular mechanisms of activation. Free Radic Biol Med.

[248] Chen K, Kirber MT, Xiao H, Yang Y, Keaney JF (2008). Regulation of ROS signal transduction by NADPH oxidase 4 localization. J Cell Biol.

[249] Dikalov S (2011). Cross talk between mitochondria and NADPH oxidases. Free Radic Biol Med.

[250] Daiber A, Di Lisa F, Oelze M, Kroller-Schon S, Steven S, Schulz E, Munzel T (2017). Crosstalk of mitochondria with NADPH oxidase via reactive oxygen and nitrogen species signalling and its role for vascular function. Br J Pharmacol.

[251] Sartoretto JL, Kalwa H, Pluth MD, Lippard SJ, Michel T (2011). Hydrogen peroxide differentially modulates cardiac myocyte nitric oxide synthesis. Proc Natl Acad Sci.

[252] Silva BR, Pernomian L, Grando MD, Bendhack LM (2014). Phenylephrine activates eNOS Ser1177 phosphorylation and nitric oxide signaling in renal hypertensive rat aorta. Eur J Pharmacol.

[253] Koju N, Taleb A, Zhou J, Lv G, Yang J, Cao X, Lei H, Ding Q (2019). Pharmacological strategies to lower crosstalk between nicotinamide adenine dinucleotide phosphate (NADPH) oxidase and mitochondria. Biomed Pharmacother.

[254] Lee DY, Wauquier F, Eid AA, Roman LJ, Ghosh-Choudhury G, Khazim K, Block K, Gorin Y (2013). Nox4 NADPH oxidase mediates peroxynitrite-dependent uncoupling of endothelial nitric-oxide synthase and fibronectin expression in response to angiotensin II: role of mitochondrial reactive oxygen species. J Biol Chem.

[255] Förstermann U, Xia N, Li H (2017). Roles of vascular oxidative stress and nitric oxide in the pathogenesis of atherosclerosis. Circ Res.

[256] Förstermann U, Sessa WC (2012). Nitric oxide synthases: regulation and function. Eur Heart J.

[257] Verhaar MC, Westerweel PE, van Zonneveld AJ, Rabelink TJ (2004). Free radical production by dysfunctional eNOS. Heart.

[258] Bendall JK, Douglas G, McNeill E, Channon KM, Crabtree MJ (2014). Tetrahydrobiopterin in cardiovascular health and disease. Antioxid Redox Signal.

[259] Meza CA, La Favor JD, Kim DH, Hickner RC. Endothelial dysfunction: is there a hyperglycemia-induced imbalance of NOX and NOS? Int J Mol Sci. 2019;20(15). 10.3390/ijms20153775 PMC6696313 31382355

[260] Gielis JF, Quirynen L, Briedé JJ, Roelant E, Cos P, Van Schil PEY (2017). Pathogenetic role of endothelial nitric oxide synthase uncoupling during lung ischaemia–reperfusion injury†. Eur J Cardiothorac Surg.

[261] Forstermann U, Munzel T (2006). Endothelial nitric oxide synthase in vascular disease: from marvel to menace. Circulation.

[262] Oberhuber R, Riede G, Cardini B, Bernhard D, Messner B, Watschinger K, Steger C, Brandacher G, Pratschke J, Golderer G (2016). Impaired endothelial nitric oxide synthase homodimer formation triggers development of transplant vasculopathy - insights from a murine aortic transplantation model. Sci Rep.

[263] Daiber A, Xia N, Steven S, Oelze M, Hanf A, Kröller-Schön S, Münzel T, Li H (2019). New therapeutic implications of endothelial nitric oxide synthase (eNOS) function/dysfunction in cardiovascular disease. Int J Mol Sci.

[264] Li H, Forstermann U (2013). Uncoupling of endothelial NO synthase in atherosclerosis and vascular disease. Curr Opin Pharmacol.

[265] Vasileiou PVS, Evangelou K, Vlasis K, Fildisis G, Panayiotidis MI, Chronopoulos E, Passias P-G, Kouloukoussa M, Gorgoulis VG, Havaki S (2019). Mitochondrial homeostasis and cellular senescence. Cells.

[266] Correia-Melo C, Passos JF (2015). Mitochondria: are they causal players in cellular senescence?. Biochim Biophys Acta.

[267] Minamino T, Miyauchi H, Yoshida T, Ishida Y, Yoshida H, Komuro I (2002). Endothelial cell senescence in human atherosclerosis: role of telomere in endothelial dysfunction. Circulation.

[268] Childs BG, Baker DJ, Wijshake T, Conover CA, Campisi J, van Deursen JM (2016). Senescent intimal foam cells are deleterious at all stages of atherosclerosis. Science.

[269] Siasos G, Tsigkou V, Kosmopoulos M, Theodosiadis D, Simantiris S, Tagkou NM, Tsimpiktsioglou A, Stampouloglou PK, Oikonomou E, Mourouzis K (2018). Mitochondria and cardiovascular diseases-from pathophysiology to treatment. Ann Transl Med.

[270] Wang Y, Tabas I (2014). Emerging roles of mitochondria ROS in atherosclerotic lesions: causation or association?. J Atheroscler Thromb.

[271] Dorighello GG, Paim BA, Kiihl SF, Ferreira M, Catharino RR, Vercesi AE, Oliveira HCF (2016). Correlation between mitochondrial reactive oxygen and severity of atherosclerosis. Oxidative Med Cell Longev.

[272] Liu R, Liu H, Ha Y, Tilton RG, Zhang W (2014). Oxidative stress induces endothelial cell senescence via downregulation of Sirt6. Biomed Res Int.

[273] Cid-Castro C, Hernández-Espinosa DR, Morán J (2018). ROS as regulators of mitochondrial dynamics in neurons. Cell Mol Neurobiol.

[274] Ježek J, Cooper KF, Strich R (2018). Reactive oxygen species and mitochondrial dynamics: the yin and yang of mitochondrial dysfunction and cancer progression. Antioxidants (Basel).

[275] Kim B, Song YS (2016). Mitochondrial dynamics altered by oxidative stress in cancer. Free Radic Res.

[276] Hung CH-L, Cheng SS-Y, Cheung Y-T, Wuwongse S, Zhang NQ, Ho Y-S, Lee SM-Y, Chang RC-C (2018). A reciprocal relationship between reactive oxygen species and mitochondrial dynamics in neurodegeneration. Redox Biol.

[277] Caja S, Enriquez JA (2017). Mitochondria in endothelial cells: sensors and integrators of environmental cues. Redox Biol.

[278] Quintero M, Colombo SL, Godfrey A, Moncada S (2006). Mitochondria as signaling organelles in the vascular endothelium. Proc Natl Acad Sci U S A.

[279] Shenouda SM, Widlansky ME, Chen K, Xu G, Holbrook M, Tabit CE, Hamburg NM, Frame AA, Caiano TL, Kluge MA (2011). Altered mitochondrial dynamics contributes to endothelial dysfunction in diabetes mellitus. Circulation.

[280] Ong SB, Subrayan S, Lim SY, Yellon DM, Davidson SM, Hausenloy DJ (2010). Inhibiting mitochondrial fission protects the heart against ischemia/reperfusion injury. Circulation.

[281] Borodkina AV, Shatrova AN, Deryabin PI, Griukova AA, Abushik PA, Antonov SM, Nikolsky NN, Burova EB (2016). Calcium alterations signal either to senescence or to autophagy induction in stem cells upon oxidative stress. Aging.

[282] Ziegler DV, Wiley CD, Velarde MC (2015). Mitochondrial effectors of cellular senescence: beyond the free radical theory of aging. Aging Cell.

[283] Hom JR, Gewandter JS, Michael L, Sheu SS, Yoon Y (2007). Thapsigargin induces biphasic fragmentation of mitochondria through calcium-mediated mitochondrial fission and apoptosis. J Cell Physiol.

[284] Tilokani L, Nagashima S, Paupe V, Prudent J (2018). Mitochondrial dynamics: overview of molecular mechanisms. Essays Biochem.

[285] Li L, Pan R, Li R, Niemann B, Aurich A-C, Chen Y, Rohrbach S (2011). Mitochondrial biogenesis and peroxisome proliferator–activated receptor-γ coactivator-1α (PGC-1α) deacetylation by physical activity. Intact Adipocytokine Signal Required.

[286] Kauppinen A, Suuronen T, Ojala J, Kaarniranta K, Salminen A (2013). Antagonistic crosstalk between NF-kappaB and SIRT1 in the regulation of inflammation and metabolic disorders. Cell Signal.

[287] Salminen A, Kaarniranta K, Kauppinen A (2013). Crosstalk between oxidative stress and SIRT1: impact on the aging process. Int J Mol Sci.

[288] Kida Y, Goligorsky MS (2016). Sirtuins, cell senescence, and vascular aging. Can J Cardiol.

[289] Morris G, Puri BK, Walker AJ, Berk M, Walder K, Bortolasci CC, Marx W, Carvalho AF, Maes M: The compensatory antioxidant response system with a focus on neuroprogressive disorders. Prog Neuro-Psychopharmacol Biol Psychiatry 2019;95:109708. 10.1016/j.pnpbp.2019.109708 31351160

[290] Morris G, Maes M, Berk M, Puri BK (2019). Myalgic encephalomyelitis or chronic fatigue syndrome: how could the illness develop?. Metab Brain Dis.

[291] Wang M, Li G, Yang Z, Wang L, Zhang L, Wang T, Zhang Y, Zhang S, Han Y, Jia L (2017). Uncoupling protein 2 downregulation by hypoxia through repression of peroxisome proliferator-activated receptor γ promotes chemoresistance of non-small cell lung cancer. Oncotarget.

[292] Corona JC, Duchen MR (2015). PPARγ and PGC-1α as therapeutic targets in Parkinson’s. Neurochem Res.

[293] Shimasaki Y, Pan N, Messina LM, Li C, Chen K, Liu L, Cooper MP, Vita JA, Keaney JF (2013). Uncoupling protein 2 impacts endothelial phenotype via p53-mediated control of mitochondrial dynamics. Circ Res.

[294] Koziel A, Sobieraj I, Jarmuszkiewicz W (2015). Increased activity of mitochondrial uncoupling protein 2 improves stress resistance in cultured endothelial cells exposed in vitro to high glucose levels. Am J Phys Heart Circ Phys.

[295] Sun J, Pu Y, Wang P, Chen S, Zhao Y, Liu C, Shang Q, Zhu Z, Liu D (2013). TRPV1-mediated UCP2 upregulation ameliorates hyperglycemia-induced endothelial dysfunction. Cardiovasc Diabetol.

[296] Ganss R (2015). Keeping the balance right: regulator of G protein signaling 5 in vascular physiology and pathology. Prog Mol Biol Transl Sci.

[297] Bretón-Romero R, Lamas S (2014). Hydrogen peroxide signaling in vascular endothelial cells. Redox Biol.

[298] Cai H (2005). Hydrogen peroxide regulation of endothelial function: origins, mechanisms, and consequences. Cardiovasc Res.

[299] Enesa K, Ito K, Luong LA, Thorbjornsen I, Phua C, Dean J, Haskard DO, Boyle J, Adcock I, To Y (2008). Hydrogen peroxide prolongs nuclear localization of NF-κB in activated cells by suppressing negative regulatory mechanisms. J Biol Chem.

[300] Gough DR, Cotter TG (2011). Hydrogen peroxide: a Jekyll and Hyde signalling molecule. Cell Death Dis.

[301] van Bergen LA, Roos G, De Proft F (2014). From thiol to sulfonic acid: modeling the oxidation pathway of protein thiols by hydrogen peroxide. J Phys Chem A.

[302] Poole LB (2015). The basics of thiols and cysteines in redox biology and chemistry. Free Radic Biol Med.

[303] Fang J, Holmgren A (2006). Inhibition of thioredoxin and thioredoxin reductase by 4-hydroxy-2-nonenal in vitro and in vivo. J Am Chem Soc.

[304] García-Nogales P, Almeida A, Bolaños JP (2003). Peroxynitrite protects neurons against nitric oxide-mediated apoptosis: a key role for glucose-6-phosphate dehydrogenase activity in neuroprotection. J Biol Chem.

[305] Chauvin JR, Pratt DA (2017). On the reactions of thiols, sulfenic acids, and sulfinic acids with hydrogen peroxide. Angew Chem Inte Ed Engl.

[306] Cyrne L, Oliveira-Marques V, Marinho HS, Antunes F (2013). H2O2 in the induction of NF-kappaB-dependent selective gene expression. Methods Enzymol.

[307] Tilstra JS, Robinson AR, Wang J, Gregg SQ, Clauson CL, Reay DP, Nasto LA, St Croix CM, Usas A, Vo N (2012). NF-κB inhibition delays DNA damage-induced senescence and aging in mice. J Clin Invest.

[308] Vaughan S, Jat PS (2011). Deciphering the role of nuclear factor-kappaB in cellular senescence. Aging.

[309] Salminen A, Kauppinen A, Kaarniranta K (2012). Emerging role of NF-κB signaling in the induction of senescence-associated secretory phenotype (SASP). Cell Signal.

[310] Nakajima H, Mochizuki N (2017). Flow pattern-dependent endothelial cell responses through transcriptional regulation. Cell Cycle.

[311] Jha P, Das H (2017). KLF2 in regulation of NF-κB-mediated immune cell function and inflammation. Int J Mol Sci.

[312] Johnson RF, Perkins ND (2012). Nuclear factor-kappaB, p53, and mitochondria: regulation of cellular metabolism and the Warburg effect. Trends Biochem Sci.

[313] Londhe P, Yu PY, Ijiri Y, Ladner KJ, Fenger JM, London C, Houghton PJ, Guttridge DC: Classical NF-κB metabolically reprograms sarcoma cells through regulation of hexokinase 2. Front Oncol 2018, 8(104). 10.3389/fonc.2018.00104 PMC5904193 29696133

[314] Tornatore L, Thotakura AK, Bennett J, Moretti M, Franzoso G (2012). The nuclear factor kappa B signaling pathway: integrating metabolism with inflammation. Trends Cell Biol.

[315] Mussbacher M, Salzmann M, Brostjan C, Hoesel B, Schoergenhofer C, Datler H, Hohensinner P, Basílio J, Petzelbauer P, Assinger A *et al*: Cell type-specific roles of NF-κB linking inflammation and thrombosis. Front Immunol 2019, 10(85). 10.3389/fimmu.2019.00085 PMC6369217 30778349

[316] Dey P, Panga V, Raghunathan S (2016). A cytokine signalling network for the regulation of inducible nitric oxide synthase expression in rheumatoid arthritis. PLoS One.

[317] Abramson SB (2008). Nitric oxide in inflammation and pain associated with osteoarthritis. Arthritis Res Ther.

[318] Arias-Salvatierra D, Silbergeld EK, Acosta-Saavedra LC, Calderon-Aranda ES (2011). Role of nitric oxide produced by iNOS through NF-kappaB pathway in migration of cerebellar granule neurons induced by lipopolysaccharide. Cell Signal.

[319] Lima B, Forrester MT, Hess DT, Stamler JS (2010). S-nitrosylation in cardiovascular signaling. Circ Res.

[320] Nakamura T, Tu S, Akhtar Mohd W, Sunico Carmen R, S-i O, Lipton Stuart A (2013). Aberrant protein S-nitrosylation in neurodegenerative diseases. Neuron.

[321] S-i O, Lipton SA (2015). S-Nitrosylation in neurogenesis and neuronal development. Biochim Biophys Acta.

[322] Fan W, Fang R, Wu X, Liu J, Feng M, Dai G, Chen G, Wu G (2015). Shear-sensitive microRNA-34a modulates flow-dependent regulation of endothelial inflammation. J Cell Sci.

[323] Sweet DR, Fan L, Hsieh PN, Jain MK: Krüppel-like factors in vascular inflammation: mechanistic insights and therapeutic potential. Fron Cardiovasc Med 2018, 5(6). 10.3389/fcvm.2018.00006 PMC5807683 29459900

[324] Lukiw WJ (2012). NF-κB-regulated, proinflammatory miRNAs in Alzheimer’s disease. Alzheimers Res Ther.

[325] Nakamura T, Lipton SA (2016). Protein S-nitrosylation as a therapeutic target for neurodegenerative diseases. Trends Pharmacol Sci.

[326] Morris G, Berk M, Klein H, Walder K, Galecki P, Maes M (2017). Nitrosative stress, hypernitrosylation, and autoimmune responses to nitrosylated proteins: new pathways in neuroprogressive disorders including depression and chronic fatigue syndrome. Mol Neurobiol.

[327] Morris G, Walder K, Carvalho AF, Tye SJ, Lucas K, Berk M, Maes M (2018). The role of hypernitrosylation in the pathogenesis and pathophysiology of neuroprogressive diseases. Neurosci Biobehav Rev.

[328] Iwakiri Y (2011). S-nitrosylation of proteins: a new insight into endothelial cell function regulated by eNOS-derived NO. Nitric Oxide.

[329] Doulias P-T, Tenopoulou M, Greene JL, Raju K, Ischiropoulos H (2013). Nitric oxide regulates mitochondrial fatty acid metabolism through reversible protein S-nitrosylation. Sci Signal.

[330] Gould N, Doulias P-T, Tenopoulou M, Raju K, Ischiropoulos H (2013). Regulation of protein function and signaling by reversible cysteine S-nitrosylation. J Biol Chem.

[331] Xu X, Qiu H, Shi F, Wang Z, Wang X, Jin L, Chi L, Zhang Q (2019). The protein S-nitrosylation of splicing and translational machinery in vascular endothelial cells is susceptible to oxidative stress induced by oxidized low-density lipoprotein. J Proteome.

[332] Guequen A, Carrasco R, Zamorano P, Rebolledo L, Burboa P, Sarmiento J, Boric MP, Korayem A, Duran WN, Sanchez FA (2016). S-nitrosylation regulates VE-cadherin phosphorylation and internalization in microvascular permeability. Am J Phys Heart Circ Phys.

[333] Marin N, Zamorano P, Carrasco R, Mujica P, Gonzalez FG, Quezada C, Meininger CJ, Boric MP, Duran WN, Sanchez FA (2012). S-Nitrosation of beta-catenin and p120 catenin: a novel regulatory mechanism in endothelial hyperpermeability. Circ Res.

[334] Shang F, Wang SC, Hsu CY, Miao Y, Martin M, Yin Y, Wu CC, Wang YT, Wu G, Chien S (2017). MicroRNA-92a mediates endothelial dysfunction in CKD. J Am Soc Nephrol.

[335] Chen Z, Wen L, Martin M, Hsu CY, Fang L, Lin FM, Lin TY, Geary MJ, Geary GG, Zhao Y (2015). Oxidative stress activates endothelial innate immunity via sterol regulatory element binding protein 2 (SREBP2) transactivation of microRNA-92a. Circulation.

[336] Huang T, Wang-Johanning F, Zhou F, Kallon H, Wei Y (2016). MicroRNAs serve as a bridge between oxidative stress and gastric cancer (review). Int J Oncol.

[337] Engedal N, Žerovnik E, Rudov A, Galli F, Olivieri F, Procopio AD, Rippo MR, Monsurrò V, Betti M, Albertini MC (2018). From oxidative stress damage to pathways, networks, and autophagy via microRNAs. Oxidative Med Cell Longev.

[338] Heid J, Cencioni C, Ripa R, Baumgart M, Atlante S, Milano G, Scopece A, Kuenne C, Guenther S, Azzimato V (2017). Age-dependent increase of oxidative stress regulates microRNA-29 family preserving cardiac health. Sci Rep.

[339] Wang S, Guo C, Yu M, Ning X, Yan B, Zhao J, Yang A, Yan H (2018). Identification of H2O2 induced oxidative stress associated microRNAs in HLE-B3 cells and their clinical relevance to the progression of age-related nuclear cataract. BMC Ophthalmol.

[340] Wan Y, Cui R, Gu J, Zhang X, Xiang X, Liu C, Qu K, Lin T (2017). Identification of four oxidative stress-responsive microRNAs, miR-34a-5p, miR-1915-3p, miR-638, and miR-150-3p, in hepatocellular carcinoma. Oxidative Med Cell Longev.

[341] Magenta A, Cencioni C, Fasanaro P, Zaccagnini G, Greco S, Sarra-Ferraris G, Antonini A, Martelli F, Capogrossi MC (2011). miR-200c is upregulated by oxidative stress and induces endothelial cell apoptosis and senescence via ZEB1 inhibition. Cell Death Differ.

[342] Carlomosti F, D'Agostino M, Beji S, Torcinaro A, Rizzi R, Zaccagnini G, Maimone B, Di Stefano V, De Santa F, Cordisco S (2017). Oxidative stress-induced miR-200c disrupts the regulatory loop among SIRT1, FOXO1, and eNOS. Antioxid Redox Signal.

[343] Zhang W, Huang Q, Zeng Z, Wu J, Zhang Y, Chen Z (2017). Sirt1 inhibits oxidative stress in vascular endothelial cells. Oxidative Med Cell Longev.

[344] Zarzuelo MJ, Lopez-Sepulveda R, Sanchez M, Romero M, Gomez-Guzman M, Ungvary Z, Perez-Vizcaino F, Jimenez R, Duarte J (2013). SIRT1 inhibits NADPH oxidase activation and protects endothelial function in the rat aorta: implications for vascular aging. Biochem Pharmacol.

[345] Kong X, Guan J, Li J, Wei J, Wang R (2017). P66(Shc)-SIRT1 regulation of oxidative stress protects against cardio-cerebral vascular disease. Mol Neurobiol.

[346] Zhang J, Cao Q, Li S, Lu X, Zhao Y, Guan JS, Chen JC, Wu Q, Chen GQ (2013). 3-Hydroxybutyrate methyl ester as a potential drug against Alzheimer’s disease via mitochondria protection mechanism. Biomaterials.

[347] Devier DJ, Lovera JF, Lukiw WJ: Increase in NF-κB-sensitive miRNA-146a and miRNA-155 in multiple sclerosis (MS) and pro-inflammatory neurodegeneration. Front Mol Neurosci 2015, 8(5). 10.3389/fnmol.2015.00005 PMC4345893 25784854

[348] Huang Y, Crawford M, Higuita-Castro N, Nana-Sinkam P, Ghadiali SN (2012). miR-146a regulates mechanotransduction and pressure-induced inflammation in small airway epithelium. FASEB J.

[349] Cheleschi S, De Palma A, Pascarelli NA, Giordano N, Galeazzi M, Tenti S, Fioravanti A. Could oxidative stress regulate the expression of microRNA-146a and microRNA-34a in human osteoarthritic chondrocyte cultures? Int J Mol Sci. 2017;18(12). 10.3390/ijms18122660 PMC5751262 29292727

[350] Rippo MR, Olivieri F, Monsurro V, Prattichizzo F, Albertini MC, Procopio AD (2014). MitomiRs in human inflamm-aging: a hypothesis involving miR-181a, miR-34a and miR-146a. Exp Gerontol.

[351] Giuliani A, Prattichizzo F, Micolucci L, Ceriello A, Procopio AD, Rippo MR (2017). Mitochondrial (dys) function in inflammaging: do mitomiRs influence the energetic, oxidative, and inflammatory status of senescent cells?. Mediat Inflamm.

[352] Leirer DJ, Iyegbe CO, Di Forti M, Patel H, Carra E, Fraietta S, Colizzi M, Mondelli V, Quattrone D, Lally J (2019). Differential gene expression analysis in blood of first episode psychosis patients. Schizophr Res.

[353] Pesce M, Ferrone A, Rizzuto A, Tatangelo R, Iezzi I, Ladu S, Franceschelli S, Speranza L, Patruno A, Felaco M, et al. The SHP-1 expression is associated with cytokines and psychopathological status in unmedicated first episode schizophrenia patients. Brain Behav Immun. 2014;41. 10.1016/j.bbi.2014.04.008 24793756

[354] Miklowitz DJ, Portnoff LC, Armstrong CC, Keenan-Miller D, Breen EC, Muscatell KA, Eisenberger NI, Irwin MR (2016). Inflammatory cytokines and nuclear factor-kappa B activation in adolescents with bipolar and major depressive disorders. Psychiatry Res.

[355] Spiliotaki M, Salpeas V, Malitas P, Alevizos V, Moutsatsou P (2006). Altered glucocorticoid receptor signaling cascade in lymphocytes of bipolar disorder patients. Psychoneuroendocrinology.

[356] Ormonde do Carmo MB, Mendes-Ribeiro AC, Matsuura C, Pinto VL, Mury WV, Pinto NO, Moss MB, Ferraz MR, Brunini TM (2015). Major depression induces oxidative stress and platelet hyperaggregability. J Psychiatr Res.

[357] Cai L, Xu L, Wei L, Chen W (2017). Relationship of mean platelet volume to MDD: a retrospective study. Shanghai Arch Psychiatry.

[358] Qiu H, Liu Y, He H, Wu Y, He W, Huang G, He J (2018). The association between mean platelet volume levels and poststroke depression. Brain Behav.

[359] Fontoura PC, Pinto VL, Matsuura C, Resende Ade C, de Bem GF, Ferraz MR, Cheniaux E, Brunini TM, Mendes-Ribeiro AC (2012). Defective nitric oxide-cyclic guanosine monophosphate signaling in patients with bipolar disorder: a potential role for platelet dysfunction. Psychosom Med.

[360] Wysokiński A, Szczepocka E (2016). Platelet parameters (PLT, MPV, P-LCR) in patients with schizophrenia, unipolar depression and bipolar disorder. Psychiatry Res.

[361] Mert DG, Terzi H (2016). Mean platelet volume in bipolar disorder: the search for an ideal biomarker. Neuropsychiatr Dis Treat.

[362] Inanli I, Aydin M, Caliskan AM, Eren I (2019). Neutrophil/lymphocyte ratio, monocyte/lymphocyte ratio, and mean platelet volume as systemic inflammatory markers in different states of bipolar disorder. Nordic J Psychiatry.

[363] Wu CC, Tsai FM, Chen ML, Wu S, Lee MC, Tsai TC, Wang LK, Wang CH (2016). Antipsychotic drugs inhibit platelet aggregation via P2Y 1 and P2Y 12 receptors. Biomed Res Int.

[364] Semiz M, Yucel H, Kavakci O, Yildirim O, Zorlu A, Yilmaz MB, Kucukdurmaz Z, Canan F (2013). Atypical antipsychotic use is an independent predictor for the increased mean platelet volume in patients with schizophrenia: a preliminary study. J Res Med Sci.

[365] Lee J, Powell V, Remington G (2014). Mean platelet volume in schizophrenia unaltered after 1year of clozapine exposure. Schizophr Res.

[366] Camkurt MA, Findikli E, Izci F, Kurutas EB, Tuman TC (2016). Evaluation of malondialdehyde, superoxide dismutase and catalase activity and their diagnostic value in drug naive, first episode, non-smoker major depression patients and healthy controls. Psychiatry Res.

[367] Geaghan M, Cairns MJ (2015). MicroRNA and posttranscriptional dysregulation in psychiatry. Biol Psychiatry.

[368] He K, Guo C, Guo M, Tong S, Zhang Q, Sun H, He L, Shi Y (2019). Identification of serum microRNAs as diagnostic biomarkers for schizophrenia. Hereditas.

[369] Vaccarino V, Brennan M-L, Miller AH, Bremner JD, Ritchie JC, Lindau F, Veledar E, Su S, Murrah NV, Jones L (2008). Association of major depressive disorder with serum myeloperoxidase and other markers of inflammation: a twin study. Biol Psychiatry.

[370] Talarowska M, Szemraj J, Galecki P (2015). Myeloperoxidase gene expression and cognitive functions in depression. Adv Med Sci.

[371] Selek S, Altindag A, Saracoglu G, Aksoy N (2015). Oxidative markers of myeloperoxidase and catalase and their diagnostic performance in bipolar disorder. J Affect Disord.

[372] Reponen E, Dieset I, Tesli M, Mørch RH, Steen NE, Hope S, Vedal TSJ, Aas M, Szabo A, Gohar SM (2019). T8. Atherogenic lipid ratios related to myeloperoxidase and C-reactive protein levels in psychotic disorders. Schizophr Bull.

[373] Kesebir S, Süner O, Yaylaci ET, Bayrak A, Turan C (2013). Increased uric acid levels in bipolar disorder: is it trait or state?. J Biol Regul Homeost Agents.

[374] Albert U, De Cori D, Aguglia A, Barbaro F, Bogetto F, Maina G (2015). Increased uric acid levels in bipolar disorder subjects during different phases of illness. J Affect Disord.

[375] Bartoli F, Crocamo C, Clerici M, Carra G (2017). Allopurinol as add-on treatment for mania symptoms in bipolar disorder: systematic review and meta-analysis of randomised controlled trials. Br J Psychiatry.

[376] Chen AT, Malmstrom T, Nasrallah HA (2018). Allopurinol augmentation in acute mania: a meta-analysis of placebo-controlled trials. J Affect Disord.

[377] Gültekin BK, Kesebir S, Kabak SG, Ergün FF, Tatlidil Yaylaci E (2014). Are uric acid levels different from healthy subjects in bipolar affective disorder and schizophrenia?: relationship between clinical improvement and episode severity in male patients. Noro Psikiyatr Ars.

[378] Michel TM, Sheldrick AJ, Camara S, Grunblatt E, Schneider F, Riederer P (2011). Alteration of the pro-oxidant xanthine oxidase (XO) in the thalamus and occipital cortex of patients with schizophrenia. World J Biol Psychiatry.

[379] Godin O, Llorca PM, Girerd N, Leboyer M, Fond G (2015). Metabolic syndrome, abdominal obesity and hyperuricemia in schizophrenia: results from the FACE-SZ dataset. Eur Psychiatry.

[380] Rajan S, Zalpuri I, Harrington A, Cimpeanu C, Song X, Fan X (2016). Relationship between serum uric acid level and cardiometabolic risks in nondiabetic patients with schizophrenia. Int Clin Psychopharmacol.

[381] Nagamine T (2010). Abnormal laboratory values during the acute and recovery phases in schizophrenic patients: a retrospective study. Neuropsychiatr Dis Treat.

[382] Yao JK, Dougherty GG, Reddy RD, Keshavan MS, Montrose DM, Matson WR, McEvoy J, Kaddurah-Daouk R (2010). Homeostatic imbalance of purine catabolism in first-episode neuroleptic-naïve patients with schizophrenia. PLoS One.

[383] Yao JK, Dougherty GG, Reddy RD, Matson WR, Kaddurah-Daouk R, Keshavan MS (2013). Associations between purine metabolites and monoamine neurotransmitters in first-episode psychosis. Front Cell Neurosci.

[384] Herken H, Gurel A, Selek S, Armutcu F, Ozen ME, Bulut M, Kap O, Yumru M, Savas HA, Akyol O (2007). Adenosine deaminase, nitric oxide, superoxide dismutase, and xanthine oxidase in patients with major depression: impact of antidepressant treatment. Arch Med Res.

[385] Michel TM, Camara S, Tatschner T, Frangou S, Sheldrick AJ, Riederer P, Grunblatt E (2010). Increased xanthine oxidase in the thalamus and putamen in depression. World J Biol Psychiatry.

[386] Dos Santos Oliveira PM, Santos V, Coroa M, Ribeiro J, Madeira N (2019). Serum uric acid as a predictor of bipolarity in individuals with a major depressive episode. Bipolar Disord.

[387] Black CN, Bot M, Scheffer PG, Snieder H, Penninx BWJH (2018). Uric acid in major depressive and anxiety disorders. J Affect Disord.

[388] Bartoli F, Trotta G, Crocamo C, Malerba MR, Clerici M, Carrà G (2018). Antioxidant uric acid in treated and untreated subjects with major depressive disorder: a meta-analysis and meta-regression. Eur Arch Psychiatry Clin Neurosci.

[389] Komaki Y, Sugiura H, Koarai A, Tomaki M, Ogawa H, Akita T, Hattori T, Ichinose M (2005). Cytokine-mediated xanthine oxidase upregulation in chronic obstructive pulmonary disease's airways. Pulm Pharmacol Ther.

[390] Xuan J, Pan G, Qiu Y, Yang L, Su M, Liu Y, Chen J, Feng G, Fang Y, Jia W (2011). Metabolomic profiling to identify potential serum biomarkers for schizophrenia and risperidone action. J Proteome Res.

[391] Gersch C, Palii SP, Imaram W, Kim KM, Karumanchi SA, Angerhofer A, Johnson RJ, Henderson GN (2009). Reactions of peroxynitrite with uric acid: formation of reactive intermediates, alkylated products and triuret, and in vivo production of triuret under conditions of oxidative stress. Nucleosides Nucleotides Nucleic Acids.

[392] Sautin YY, Johnson RJ (2008). Uric acid: the oxidant-antioxidant paradox. Nucleosides Nucleotides Nucleic Acids.

[393] Dietrich-Muszalska A, Malinowska J, Olas B, Głowacki R, Bald E, Wachowicz B, Rabe-Jabłońska J (2012). The oxidative stress may be induced by the elevated homocysteine in schizophrenic patients. Neurochem Res.

[394] Lin KM, Lu CL, Hung KC, Wu PC, Pan CF, Wu CJ, Syu RS, Chen JS, Hsiao PJ, Lu KC. The paradoxical role of uric acid in osteoporosis. Nutrients. 2019;11(9). 10.3390/nu11092111 PMC6769742 31491937

[395] Tchalla AE, Wellenius GA, Sorond FA, Travison TG, Dantoine T, Lipsitz LA. Elevated circulating vascular cell adhesion molecule-1 (sVCAM-1) is associated with concurrent depressive symptoms and cerebral white matter hyperintensities in older adults. BMC Geriatr. 2015;15(1). 10.1186/s12877-015-0063-7 PMC4453284 26040277

[396] Müller N. The role of intercellular adhesion molecule-1 in the pathogenesis of psychiatric disorders. Front Pharmacol. 2019;10. 10.3389/fphar.2019.01251 PMC6883971 31824303

[397] Lopez-Vilchez I, Diaz-Ricart M, Navarro V, Torramade S, Zamorano-Leon J, Lopez-Farre A, Galan AM, Gasto C, Escolar G (2016). Endothelial damage in major depression patients is modulated by SSRI treatment, as demonstrated by circulating biomarkers and an in vitro cell model. Transl Psychiatry.

[398] Poljask B, Milisav I. NAD+ as the link between oxidative stress, inflammation, caloric restriction, exercise, DNA repair, longevity, and health span. Rejuvenation Res 2016;19(5):406–413. 10.1089/rej.2015.1767 26725653

[399] Bot M, Chan MK, Jansen R, Lamers F, Vogelzangs N, Steiner J, Leweke FM, Rothermundt M, Cooper J, Bahn S (2015). Serum proteomic profiling of major depressive disorder. Transl Psychiatry.

[400] Kalkman HO (2020). The association between vascular inflammation and depressive disorder. Causality, biomarkers and targeted treatment. Pharmaceuticals (Basel).

[401] van Sloten TT, Schram MT, Adriaanse MC, Dekker JM, Nijpels G, Teerlink T, Scheffer PG, Pouwer F, Schalkwijk CG, Stehouwer CD (2014). Endothelial dysfunction is associated with a greater depressive symptom score in a general elderly population: the Hoorn Study. Psychol Med.

[402] Peng L, Bi S, Liu X, Long T, Zhao Y, Li F, Yang T, Zhang C (2020). Association between depressive symptoms and arterial stiffness: a cross-sectional study in the general Chinese population. BMJ Open.

[403] Palta P, Samuel LJ, Miller ERI, Szanton SL (2014). Depression and oxidative stress: results from a meta-analysis of observational studies. Psychosom Med.

[404] Greaney JL, Saunders EFH, Santhanam L, Alexander LM (2019). Oxidative stress contributes to microvascular endothelial dysfunction in men and women with major depressive disorder. Circ Res.

[405] Tonhajzerova I, Sekaninova N, Bona Olexova L, Visnovcova Z (2020). Novel insight into neuroimmune regulatory mechanisms and biomarkers linking major depression and vascular diseases: the dilemma continues. Int J Mol Sci.

[406] Brietzke E, Teixeira AL (2010). Similar immune profile in bipolar disorder and schizophrenia: selective increase in soluble tumor necrosis factor receptor I and von Willebrand factor. Bipolar Disord.

[407] Reininghaus EZ, Lackner N, Birner A, Bengesser S, Fellendorf FT, Platzer M, Rieger A, Queissner R, Kainzbauer N, Reininghaus B (2016). Extracellular matrix proteins matrix metallopeptidase 9 (MMP9) and soluble intercellular adhesion molecule 1 (sICAM-1) and correlations with clinical staging in euthymic bipolar disorder. Bipolar Disord.

[408] Schaefer M, Sarkar S, Schwarz M, Friebe A (2016). Soluble intracellular adhesion molecule-1 in patients with unipolar or bipolar affective disorders: results from a pilot trial. Neuropsychobiology.

[409] Kali A, Shetty KSR (2014). Endocan: a novel circulating proteoglycan. Indian J Pharmacol.

[410] Blanco FJ, Bernabéu C (2012). The splicing factor SRSF1 as a marker for endothelial senescence. Front Physiol.

[411] Cai HQ, Catts VS, Webster MJ, Galletly C, Liu D, O’Donnell M, Weickert TW, Weickert CS (2020). Increased macrophages and changed brain endothelial cell gene expression in the frontal cortex of people with schizophrenia displaying inflammation. Mol Psychiatry.

[412] Dieset I, Haukvik UK, Melle I, Røssberg JI, Ueland T, Hope S, Dale AM, Djurovic S, Aukrust P, Agartz I (2015). Association between altered brain morphology and elevated peripheral endothelial markers--implications for psychotic disorders. Schizophr Res.

[413] Hope S, Ueland T, Steen NE, Dieset I, Lorentzen S, Berg AO, Agartz I, Aukrust P, Andreassen OA (2013). Interleukin 1 receptor antagonist and soluble tumor necrosis factor receptor 1 are associated with general severity and psychotic symptoms in schizophrenia and bipolar disorder. Schizophr Res.

[414] Cristiano VB, Vieira Szortyka MF, Lobato MI, Ceresér KM, Belmonte-de-Abreu P (2017). Postural changes in different stages of schizophrenia is associated with inflammation and pain: a cross-sectional observational study. Int J Psychiatry Clin Pract.

[415] Schwarz MJ, Riedel M, Ackenheil M, Müller N (2000). Decreased levels of soluble intercellular adhesion molecule-1 (sICAM-1) in unmedicated and medicated schizophrenic patients. Biol Psychiatry.

[416] Shcherbakova IV, Kaleda VG, Barkhatova AN, Kliushnik TP (2005). Markers of endothelial dysfunction in attack-like schizophrenia. Zhurnal nevrologii i psikhiatrii imeni SS Korsakova.

[417] Kimmel SE, Schelleman H, Berlin JA, Oslin DW, Weinstein RB, Kinman JL, Sauer WH, Lewis JD (2011). The effect of selective serotonin re-uptake inhibitors on the risk of myocardial infarction in a cohort of patients with depression. Br J Clin Pharmacol.

[418] Santangelo A, Testaì M, Barbagallo P, Crisafulli C, Grasso S, Manuele S, Muscarà G, Rizzotto M, Tomarchio M, Maugeri D (2009). Use of specific serotonin reuptake inhibitors (SSRIs) (sertraline or citalopram) in the treatment of depression reduces the cardiovascular risk in the elderly: evidence from a Sicilian population >80 years recovered in the assisted sanitary residences (RSA). Arch Gerontol Geriatr.

[419] Tseng YL, Chiang ML, Huang TF, Su KP, Lane HY, Lai YC (2010). A selective serotonin reuptake inhibitor, citalopram, inhibits collagen-induced platelet aggregation and activation. Thromb Res.

[420] McCloskey DJ, Postolache TT, Vittone BJ, Nghiem KL, Monsale JL, Wesley RA, Rick ME (2008). Selective serotonin reuptake inhibitors: measurement of effect on platelet function. Transl Res.

[421] van Zyl LT, Lespérance F, Frasure-Smith N, Malinin AI, Atar D, Laliberté MA, Serebruany VL (2009). Platelet and endothelial activity in comorbid major depression and coronary artery disease patients treated with citalopram: the Canadian Cardiac Randomized Evaluation of Antidepressant and Psychotherapy Efficacy Trial (CREATE) biomarker sub-study. J Thromb Thrombolysis.

[422] Pizzi C, Mancini S, Angeloni L, Fontana F, Manzoli L, Costa GM (2009). Effects of selective serotonin reuptake inhibitor therapy on endothelial function and inflammatory markers in patients with coronary heart disease. Clin Pharmacol Ther.

[423] Kokras N, Papadopoulou E, Georgiopoulos G, Dalla C, Petropoulos I, Kontogiannis C, Laina A, Bampatsias D, Stellos K, Kouzoupis AV (2019). The effect of treatment response on endothelial function and arterial stiffness in depression. A prospective study. J Affect Disord.

[424] Hantsoo L, Czarkowski KA, Child J, Howes C, Epperson CN (2014). Selective serotonin reuptake inhibitors and endothelial function in women. J Women's Health (Larchmt).

[425] Mohammadianinejad SE, Majdinasab N, Sajedi SA, Abdollahi F, Moqaddam MM, Sadr F (2014). The effect of lithium in post-stroke motor recovery: a double-blind, placebo-controlled, randomized clinical trial. Clin Neuropharmacol.

[426] Lyoo IK, Dager SR, Kim JE, Yoon SJ, Friedman SD, Dunner DL, Renshaw PF (2010). Lithium-induced gray matter volume increase as a neural correlate of treatment response in bipolar disorder: a longitudinal brain imaging study. Neuropsychopharmacology.

[427] Li W, Li R, Zhao S, Jiang C, Liu Z, Tang X (2018). Lithium posttreatment alleviates blood–brain barrier injury after intracerebral hemorrhage in rats. Neuroscience.

[428] Wang Z-f, Fessler EB, Chuang D-M (2011). Beneficial effects of mood stabilizers lithium, valproate and lamotrigine in experimental stroke models. Acta Pharmacol Sin.

[429] Murugavel S, Bugyei-Twum A, Matkar PN, Al-Mubarak H, Chen HH, Adam M, Jain S, Narang T, Abdin RM, Qadura M *et al*: Valproic acid induces endothelial-to-mesenchymal transition-like phenotypic switching. Front Pharmacol 2018, 9(737). 10.3389/fphar.2018.00737 PMC6050396 30050438

[430] Ellingrod VL, Taylor SF, Brook RD, Evans SJ, Zöllner SK, Grove TB, Gardner KM, Bly MJ, Pop-Busui R, Dalack G (2011). Dietary, lifestyle and pharmacogenetic factors associated with arteriole endothelial-dependent vasodilatation in schizophrenia patients treated with atypical antipsychotics (AAPs). Schizophr Res.

[431] Burghardt K, Grove T, Ellingrod V (2014). Endothelial nitric oxide synthetase genetic variants, metabolic syndrome and endothelial function in schizophrenia. J Psychopharmacol.

[432] Xu H, Zhuang X (2019). Atypical antipsychotics-induced metabolic syndrome and nonalcoholic fatty liver disease: a critical review. Neuropsychiatr Dis Treat.

[433] Kavzoglu S, Hariri A (2013). Intracellular adhesion molecule (ICAM-1), vascular cell adhesion molecule (VCAM-1) and E-selectin levels in first episode schizophrenic patients. Bull Clin Psychopharmacol.

[434] Clark AM, DesMeules M, Luo W, Duncan AS, Wielgosz A (2009). Socioeconomic status and cardiovascular disease: risks and implications for care. Nat Rev Cardiol.

[435] Cohen S, Janicki-Deverts D, Chen E, Matthews KA (2010). Childhood socioeconomic status and adult health. Ann N Y Acad Sci.

[436] Schultz WM, Kelli HM, Lisko JC, Varghese T, Shen J, Sandesara P, Quyyumi AA, Taylor HA, Gulati M, Harold JG (2018). Socioeconomic status and cardiovascular outcomes. Circulation.

[437] East P, Doom J, Delker E, Blanco E, Burrows R, Correa-Burrows P, Lozoff B, Gahagan S (2020). Childhood socioeconomic hardship, family conflict, and young adult hypertension: the Santiago Longitudinal Study. Soc Sci Med.

[438] Reid BM, Doom JR, Argote RB, Correa-Burrows P, Lozoff B, Blanco E, Gahagan S (2020). Pathways to inflammation in adolescence through early adversity, childhood depressive symptoms, and body mass index: a prospective longitudinal study of Chilean infants. Brain Behav Immun.

[439] Kivimäki M, Vahtera J, Tabák AG, Halonen JI, Vineis P, Pentti J, Pahkala K, Rovio S, Viikari J, Kähönen M (2018). Neighbourhood socioeconomic disadvantage, risk factors, and diabetes from childhood to middle age in the Young Finns Study: a cohort study. Lancet Public Health.

[440] Lee SC, DelPozo-Banos M, Lloyd K, Jones I, Walters JTR, Owen MJ, O'Donovan M, John A (2020). Area deprivation, urbanicity, severe mental illness and social drift — a population-based linkage study using routinely collected primary and secondary care data. Schizophr Res.

[441] Martin JL, McLean G, Park J, Martin DJ, Connolly M, Mercer SW, Smith DJ (2014). Impact of socioeconomic deprivation on rate and cause of death in severe mental illness. BMC Psychiatry.

[442] Diniz BS, Reynolds Iii CF, Sibille E, Bot M, Penninx BWJH (2019). Major depression and enhanced molecular senescence abnormalities in young and middle-aged adults. Transl Psychiatry.

[443] Palmer AK, Gustafson B, Kirkland JL, Smith U (2019). Cellular senescence: at the nexus between ageing and diabetes. Diabetologia.

[444] Harvey A, Montezano AC, Touyz RM (2015). Vascular biology of ageing-implications in hypertension. J Mol Cell Cardiol.

[445] Kaptoge S, Di Angelantonio E, Pennells L, Wood AM, White IR, Gao P, Walker M, Thompson A, Sarwar N, Caslake M (2012). C-reactive protein, fibrinogen, and cardiovascular disease prediction. N Engl J Med.

[446] Esser N, Legrand-Poels S, Piette J, Scheen AJ, Paquot N (2014). Inflammation as a link between obesity, metabolic syndrome and type 2 diabetes. Diabetes Res Clin Pract.

[447] Fung TT, McCullough ML, Newby PK, Manson JE, Meigs JB, Rifai N, Willett WC, Hu FB (2005). Diet-quality scores and plasma concentrations of markers of inflammation and endothelial dysfunction. Am J Clin Nutr.

[448] Akbaraly TN, Shipley MJ, Ferrie JE, Virtanen M, Lowe G, Hamer M, Kivimaki M (2015). Long-term adherence to healthy dietary guidelines and chronic inflammation in the prospective Whitehall II study. Am J Med.

[449] Hamer M, Sabia S, Batty GD, Shipley MJ, Tabák AG, Singh-Manoux A, Kivimaki M (2012). Physical activity and inflammatory markers over 10 years: follow-up in men and women from the Whitehall II cohort study. Circulation.

[450] Jarvie JL, Whooley MA, Regan MC, Sin NL, Cohen BE (2014). Effect of physical activity level on biomarkers of inflammation and insulin resistance over 5 years in outpatients with coronary heart disease (from the Heart and Soul Study). Am J Cardiol.

[451] Motivala SJ (2011). Sleep and inflammation: psychoneuroimmunology in the context of cardiovascular disease. Ann Behav Med.

[452] Qin B, Deng Y (2015). Overexpression of circadian clock protein cryptochrome (CRY) 1 alleviates sleep deprivation-induced vascular inflammation in a mouse model. Immunol Lett.

[453] Shiels MS, Katki HA, Freedman ND, Purdue MP, Wentzensen N, Trabert B, Kitahara CM, Furr M, Li Y, Kemp TJ (2014). Cigarette smoking and variations in systemic immune and inflammation markers. J Natl Cancer Inst.

[454] Asthana A, Johnson HM, Piper ME, Fiore MC, Baker TB, Stein JH (2010). Effects of smoking intensity and cessation on inflammatory markers in a large cohort of active smokers. Am Heart J.

[455] Leclercq S, de Timary P, Delzenne NM, Stärkel P (2017). The link between inflammation, bugs, the intestine and the brain in alcohol dependence. Transl Psychiatry.

[456] Schmitz S, Abosi O, Persons J, Sinkey C, Fiedorowicz J (2018). Impact of mood on endothelial function and arterial stiffness in bipolar disorder. Heart Mind.

[457] Shi H, Feng G, Wang Z, Zhou C, Zhong G, Hu Y, Wang G (2015). Relationships between depressive symptoms and endothelial function among outpatients of a general hospital in China. Med Sci Monit.

[458] Velten J, Bieda A, Scholten S, Wannemüller A, Margraf J (2018). Lifestyle choices and mental health: a longitudinal survey with German and Chinese students. BMC Public Health.

[459] Mintzer J, Donovan KA, Kindy AZ, Lock SL, Chura LR, Barracca N (2019). Lifestyle choices and brain health. Front Med (Lausanne).

[460] Morris G, Berk M, Maes M, Carvalho AF, Puri BK (2019). Socioeconomic deprivation, adverse childhood experiences and medical disorders in adulthood: mechanisms and associations. Mol Neurobiol.

[461] Tang KL, Rashid R, Godley J, Ghali WA (2016). Association between subjective social status and cardiovascular disease and cardiovascular risk factors: a systematic review and meta-analysis. BMJ Open.

[462] Cooper DC, Milic MS, Mills PJ, Bardwell WA, Ziegler MG, Dimsdale JE (2010). Endothelial function: the impact of objective and subjective socioeconomic status on flow-mediated dilation. Ann Behav Med.

[463] Tang KL, Pilote L, Behlouli H, Godley J, Ghali WA (2018). An exploration of the subjective social status construct in patients with acute coronary syndrome. BMC Cardiovasc Disord.

[464] Kupper N, Denollet J (2018). Type D personality as a risk factor in coronary heart disease: a review of current evidence. Curr Cardiol Rep.

[465] Kim C-H, Noh I-K, Ryu JM, Bae EJ, Cho HJ, Kim MS (2020). Canonical correlation between behavioral-psychological variables and predictors of coronary artery disease prognosis. Int J Environ Res Public Health.

[466] Bezgin CH, Bezgin T, Kesebir S (2016). Temperament and character profiles and psychiatric comorbidities in patients with coronary artery or valvular heart disease: relationship with cardiac disease severity. J Clin Med Res.

[467] Eory A, Gonda X, Lang Z, Torzsa P, Kalman J, Kalabay L, Rihmer Z (2014). Personality and cardiovascular risk: association between hypertension and affective temperaments—a cross-sectional observational study in primary care settings. Eur J Gen Pract.

[468] Korosi BZ, Batta D, Gonda X, Rihmer Z, Nemcsik-Bencze Z, László A, Vecsey-Nagy M, Nemcsik J. Association between irritable affective temperament and nighttime peripheral and central systolic blood pressure in hypertension. Artery Res. 2019;25.

[469] László A, Tabák Á, Kőrösi B, Eörsi D, Torzsa P, Cseprekál O, Tislér A, Reusz G, Nemcsik-Bencze Z, Gonda X (2016). Association of affective temperaments with blood pressure and arterial stiffness in hypertensive patients: a cross-sectional study. BMC Cardiovasc Disord.

[470] Wright RJ (2009). Stress and acquired glucocorticoid resistance: a relationship hanging in the balance. J Allergy Clin Immunol.

[471] Verhoeven F, Prati C, Maguin-Gaté K, Wendling D, Demougeot C (2016). Glucocorticoids and endothelial function in inflammatory diseases: focus on rheumatoid arthritis. Arthritis Res Ther.

[472] Zielińska KA, Van Moortel L, Opdenakker G, De Bosscher K, Van den Steen PE: Endothelial response to glucocorticoids in inflammatory diseases. Front Immunol 2016, 7(592). 10.3389/fimmu.2016.00592 PMC5155119 28018358

[473] Iuchi T, Akaike M, Mitsui T, Ohshima Y, Shintani Y, Azuma H, Matsumoto T (2003). Glucocorticoid excess induces superoxide production in vascular endothelial cells and elicits vascular endothelial dysfunction. Circ Res.

[474] Brotman DJ, Girod JP, Garcia MJ, Patel JV, Gupta M, Posch A, Saunders S, Lip GYH, Worley S, Reddy S (2005). Effects of short-term glucocorticoids on cardiovascular biomarkers. J Clin Endocrinol Metabol.

[475] Haapakoski R, Mathieu J, Ebmeier KP, Alenius H, Kivimäki M (2015). Cumulative meta-analysis of interleukins 6 and 1β, tumour necrosis factor α and C-reactive protein in patients with major depressive disorder. Brain Behav Immun.

[476] Howren MB, Lamkin DM, Suls J (2009). Associations of depression with C-reactive protein, IL-1, and IL-6: a meta-analysis. Psychosom Med.

[477] Dowlati Y, Herrmann N, Swardfager W, Liu H, Sham L, Reim EK. A meta-analysis of cytokines in major depression. Biol Psychiatry. 2010;67. 10.1016/j.biopsych.2009.09.033 20015486

[478] Liu Y, Ho RC-M, Mak A (2012). Interleukin (IL)-6, tumour necrosis factor alpha (TNF-α) and soluble interleukin-2 receptors (sIL-2R) are elevated in patients with major depressive disorder: a meta-analysis and meta-regression. J Affect Disord.

[479] Sena CM, Leandro A, Azul L, Seiça R, Perry G (2018). Vascular oxidative stress: impact and therapeutic approaches. Front Physiol.

[480] Silva B, Pernomian L, Bendhack L: Contribution of oxidative stress to endothelial dysfunction in hypertension. Front Physiol 2012, 3(441). 10.3389/fphys.2012.00441 PMC3514688 23227009

[481] Zhang C (2008). The role of inflammatory cytokines in endothelial dysfunction. Basic Res Cardiol.

[482] van der Poll T, van de Veerdonk FL, Scicluna BP, Netea MG (2017). The immunopathology of sepsis and potential therapeutic targets. Nat Rev Immunol.

[483] Szałach ŁP, Lisowska KA, Cubała WJ (2019). The influence of antidepressants on the immune system. Arch Immunol Ther Exp.

[484] Gałecki P, Mossakowska-Wójcik J, Talarowska M (2018). The anti-inflammatory mechanism of antidepressants - SSRIs, SNRIs. Prog Neuro-Psychopharmacol Biol Psychiatry.

[485] Kotan VO, Sarandol E, Kirhan E, Ozkaya G, Kirli S. Effects of long-term antidepressant treatment on oxidative status in major depressive disorder: a 24-week follow-up study. Prog Neuro-Psychopharmacol Biol Psychiatry. 2011;35. 10.1016/j.pnpbp.2011.03.021 21515329

[486] Behr GA, Moreira JCF, Frey BN. Preclinical and clinical evidence of antioxidant effects of antidepressant agents: implications for the pathophysiology of major depressive disorder. Oxidative Med Cell Longev. 2012;2012. 10.1155/2012/609421 PMC3368202 22693652

[487] Morris G, Berk M (2016). The putative use of lithium in Alzheimer’s disease. Curr Alzheimer Res.

[488] Beumer W, Gibney SM, Drexhage RC, Pont-Lezica L, Doorduin J, Klein HC, Steiner J, Connor TJ, Harkin A, Versnel MA (2012). The immune theory of psychiatric diseases: a key role for activated microglia and circulating monocytes. J Leukoc Biol.

[489] Sprague AH, Khalil RA (2009). Inflammatory cytokines in vascular dysfunction and vascular disease. Biochem Pharmacol.

[490] Ince C, Mayeux PR, Nguyen T, Gomez H, Kellum JA, Ospina-Tascon GA, Hernandez G, Murray P, De Backer D (2016). The endothelium in sepsis. Shock.

[491] Iba T, Levy JH (2019). Derangement of the endothelial glycocalyx in sepsis. J Thromb Haemostasis.

[492] Uchimido R, Schmidt EP, Shapiro NI (2019). The glycocalyx: a novel diagnostic and therapeutic target in sepsis. Crit Care.

[493] Steinhagen F, Schmidt SV, Schewe J-C, Peukert K, Klinman DM, Bode C (2020). Immunotherapy in sepsis - brake or accelerate?. Pharmacol Ther.

[494] Chang JC (2019). Sepsis and septic shock: endothelial molecular pathogenesis associated with vascular microthrombotic disease. Thromb J.

[495] Genkel VV, Shaposhnik II (2020). Conceptualization of heterogeneity of chronic diseases and atherosclerosis as a pathway to precision medicine: endophenotype, endotype, and residual cardiovascular risk. Int J Chronic Dis.

[496] Vardon-Bounes F, Ruiz S, Gratacap M-P, Garcia C, Payrastre B, Minville V (2019). Platelets are critical key players in sepsis. Int J Mol Sci.

[497] Li X, Wang P, Xu X, Wang Y, Xia Y, Wang D (2009). Simvastatin increases the activity of endothelial nitric oxide synthase via enhancing phosphorylation. J Huazhong Univ Sci Technol Med Sci.

[498] Rossoni LV, Wareing M, Wenceslau CF, Al-Abri M, Cobb C, Austin C (2011). Acute simvastatin increases endothelial nitric oxide synthase phosphorylation via AMP-activated protein kinase and reduces contractility of isolated rat mesenteric resistance arteries. Clin Sci (London).

[499] Oesterle A, Laufs U, Liao JK (2017). Pleiotropic effects of statins on the cardiovascular system. Circ Res.

[500] Agewall S, Hernberg A (2006). Atorvastatin normalizes endothelial function in healthy smokers. Clin Sci (London).

[501] Martínez Aguilar E, De Haro Miralles J, Flórez González A, Varela Casariego C, Bleda Moreno S, Acín García F (2009). In vivo confirmation of the role of statins in reducing nitric oxide and C-reactive protein levels in peripheral arterial disease. Eur J Vasc Endovasc Surg.

[502] Margaritis M, Channon KM, Antoniades C (2014). Statins as regulators of redox state in the vascular endothelium: beyond lipid lowering. Antioxid Redox Signal.

[503] Beckman JA, Liao JK, Hurley S, Garrett LA, Chui D, Mitra D, Creager MA (2004). Atorvastatin restores endothelial function in normocholesterolemic smokers independent of changes in low-density lipoprotein. Circ Res.

[504] John S, Schneider MP, Delles C, Jacobi J, Schmieder RE (2005). Lipid-independent effects of statins on endothelial function and bioavailability of nitric oxide in hypercholesterolemic patients. Am Heart J.

[505] Sikora J, Kostka B, Marczyk I, Krajewska U, Chałubiński M, Broncel M (2013). Effect of statins on platelet function in patients with hyperlipidemia. Arch Med Sci.

[506] Barale C, Frascaroli C, Senkeev R, Cavalot F, Russo I (2018). Simvastatin effects on inflammation and platelet activation markers in hypercholesterolemia. Biomed Res Int.

[507] Zhou T, Zhou SH, Qi SS, Shen XQ, Zeng GF, Zhou HN (2006). The effect of atorvastatin on serum myeloperoxidase and CRP levels in patients with acute coronary syndrome. Clin Chim Acta.

[508] Stenvinkel P, Rodríguez-Ayala E, Massy ZA, Qureshi AR, Barany P, Fellström B, Heimburger O, Lindholm B, Alvestrand A (2006). Statin treatment and diabetes affect myeloperoxidase activity in maintenance hemodialysis patients. Clin J Am Soc Nephrol.

[509] Yoshida T, Yamashita M, Iwai M, Hayashi M (2016). Endothelial Kruppel-like factor 4 mediates the protective effect of statins against ischemic AKI. J Am Soc Nephrol.

[510] Gao Y, Liu XF, Lu XC, Ma C, Cao J, Fan L (2012). Protective effects of atorvastatin against oxidized LDL-induced downregulation of KLF expression in EA.hy926 cells. Int J Mol Med.

[511] Li Y, Xian M, Yang B, Ying M, He Q (2017). Inhibition of KLF4 by statins reverses adriamycin-induced metastasis and cancer stemness in osteosarcoma cells. Stem Cell Rep.

[512] Alis R, Sanchis-Gomar F, Risso-Ballester J, Perez-Quilis C, Cortell-Ballester J, Romagnoli M, Blesa JR, Emanuele E (2015). Inhibition of xanthine oxidase to prevent statin-induced myalgia and rhabdomiolysis. Atherosclerosis.

[513] Stewart RA (2009). Predicting benefit from statins by C-reactive protein, LDL-cholesterol or absolute cardiovascular risk. Futur Cardiol.

[514] Rosendo AB, Lima LO, Dal-Pizzol F, Almeida S (2010). Lipid and C-reactive protein levels, cardiovascular disease risk factors and simvastatin treatment in Brazilian individuals. Inflammation.

[515] Datta S, Iqbal Z, Prasad KR (2011). Comparison between serum hsCRP and LDL cholesterol for search of a better predictor for ischemic heart disease. Indian J Clin Biochem.

[516] Mandosi E, Fallarino M, Gatti A, Carnovale A, Rossetti M, Lococo E, Buchetti B, Filetti S, Lenti L, Morano S (2010). Atorvastatin downregulates monocyte CD36 expression, nuclear NFkappaB and TNFalpha levels in type 2 diabetes. J Atheroscler Thromb.

[517] Xie W, Li P, Wang Z, Chen J, Lin Z, Liang X, Mo Y (2014). Rosuvastatin may reduce the incidence of cardiovascular events in patients with acute coronary syndromes receiving percutaneous coronary intervention by suppressing miR-155/SHIP-1 signaling pathway. Cardiovasc Ther.

[518] Rezaie-Majd A, Maca T, Bucek RA, Valent P, Muller MR, Husslein P, Kashanipour A, Minar E, Baghestanian M (2002). Simvastatin reduces expression of cytokines interleukin-6, interleukin-8, and monocyte chemoattractant protein-1 in circulating monocytes from hypercholesterolemic patients. Arterioscler Thromb Vasc Biol.

[519] Zinellu A, Paliogiannis P, Usai MF, Carru C, Mangoni AA (2019). Effect of statin treatment on circulating malondialdehyde concentrations: a systematic review and meta-analysis. Ther Adv Chronic Dis.

[520] Rasmussen ST, Andersen JT, Nielsen TK, Cejvanovic V, Petersen KM, Henriksen T, Weimann A, Lykkesfeldt J, Poulsen HE (2016). Simvastatin and oxidative stress in humans: a randomized, double-blinded, placebo-controlled clinical trial. Redox Biol.

[521] Bouitbir J, Sanvee GM, Panajatovic MV, Singh F, Krahenbuhl S: Mechanisms of statin-associated skeletal muscle-associated symptoms. Pharmacol Res 2019:104201. 10.1016/j.phrs.2019.03.010 30877064

[522] Yilmaz MI, Baykal Y, Kilic M, Sonmez A, Bulucu F, Aydin A, Sayal A, Kocar IH (2004). Effects of statins on oxidative stress. Biol Trace Elem Res.

[523] Liu A, Wu Q, Guo J, Ares I, Rodriguez JL, Martinez-Larranaga MR, Yuan Z, Anadon A, Wang X, Martinez MA (2019). Statins: adverse reactions, oxidative stress and metabolic interactions. Pharmacol Ther.

[524] Ramachandran R, Wierzbicki AS (2017). Statins, muscle disease and mitochondria. J Clin Med.

[525] Abid H, Cartier D, Hamieh A, Francois-Bellan AM, Bucharles C, Pothion H, Manecka DL, Leprince J, Adriouch S, Boyer O (2019). AMPK activation of PGC-1alpha/NRF-1-dependent SELENOT gene transcription promotes PACAP-induced neuroendocrine cell differentiation through tolerance to oxidative stress. Mol Neurobiol.

[526] Lin ZF, Wang CY, Shen LJ, Hsiao FY, Lin Wu FL (2016). Statin use and the risk for incident diabetes mellitus in patients with acute coronary syndrome after percutaneous coronary intervention: a population-based retrospective cohort study in Taiwan. Can J Diabetes.

[527] Urbano F, Bugliani M, Filippello A, Scamporrino A, Di Mauro S, Di Pino A, Scicali R, Noto D, Rabuazzo AM, Averna M (2017). Atorvastatin but not pravastatin impairs mitochondrial function in human pancreatic islets and rat β-cells. Direct effect of oxidative stress. Sci Rep.

[528] Qu H, Y-y M, Chai H, Liang F, J-y Z, Z-y G, D-z S (2018). The effect of statin treatment on circulating coenzyme Q10 concentrations: an updated meta-analysis of randomized controlled trials. Eur J Med Res.

[529] Banach M, Serban C, Ursoniu S, Rysz J, Muntner P, Toth PP, Jones SR, Rizzo M, Glasser SP, Watts GF (2015). Statin therapy and plasma coenzyme Q10 concentrations--a systematic review and meta-analysis of placebo-controlled trials. Pharmacol Res.

[530] Qu H, Guo M, Chai H, Wang W, Gao ZY, Shi DZ (2018). Effects of coenzyme Q10 on statin-induced myopathy: an updated meta-analysis of randomized controlled trials. J Am Heart Assoc.

[531] Banach M, Serban C, Sahebkar A, Ursoniu S, Rysz J, Muntner P, Toth PP, Jones SR, Rizzo M, Glasser SP (2015). Effects of coenzyme Q10 on statin-induced myopathy: a meta-analysis of randomized controlled trials. Mayo Clin Proc.

[532] Morris G, Anderson G, Berk M, Maes M (2013). Coenzyme Q10 depletion in medical and neuropsychiatric disorders: potential repercussions and therapeutic implications. Mol Neurobiol.

[533] Mantle D, Hargreaves I (2019). Coenzyme Q10 and degenerative disorders affecting longevity: an overview. Antioxidants.

[534] Gao L, Mao Q, Cao J, Wang Y, Zhou X, Fan L (2012). Effects of coenzyme Q10 on vascular endothelial function in humans: a meta-analysis of randomized controlled trials. Atherosclerosis.

[535] Huo J, Xu Z, Hosoe K, Kubo H, Miyahara H, Dai J, Mori M, Sawashita J, Higuchi K (2018). Coenzyme Q10 prevents senescence and dysfunction caused by oxidative stress in vascular endothelial cells. Oxidative Med Cell Longev.

[536] Yang YK, Wang LP, Chen L, Yao XP, Yang KQ, Gao LG, Zhou XL (2015). Coenzyme Q10 treatment of cardiovascular disorders of ageing including heart failure, hypertension and endothelial dysfunction. Clin Chim Acta.

[537] Rossman MJ, Santos-Parker JR, Steward CAC, Bispham NZ, Cuevas LM, Rosenberg HL, Woodward KA, Chonchol M, Gioscia-Ryan RA, Murphy MP (2018). Chronic supplementation with a mitochondrial antioxidant (mitoQ) improves vascular function in healthy older adults. Hypertension.

[538] Dai Y-L, Luk T-H, Yiu K-H, Wang M, Yip PMC, Lee SWL, Li S-W, Tam S, Fong B, Lau C-P (2011). Reversal of mitochondrial dysfunction by coenzyme Q10 supplement improves endothelial function in patients with ischaemic left ventricular systolic dysfunction: a randomized controlled trial. Atherosclerosis.

[539] Hamilton SJ, Chew GT, Watts GF. Coenzyme Q_10_ improves endothelial dysfunction in statin-treated type 2 diabetic patients. Diabetes Care. 2009;32:810–2. 10.2337/dc08-1736 PMC2671099 19228872

[540] Tiano L, Belardinelli R, Carnevali P, Principi F, Seddaiu G, Littarru GP (2007). Effect of coenzyme Q10 administration on endothelial function and extracellular superoxide dismutase in patients with ischaemic heart disease: a double-blind, randomized controlled study. Eur Heart J.

[541] Suarez-Rivero JM, Pastor-Maldonado CJ, de la Mata M, Villanueva-Paz M, Povea-Cabello S, Alvarez-Cordoba M, Villalon-Garcia I, Suarez-Carrillo A, Talaveron-Rey M, Munuera M, et al. Atherosclerosis and coenzyme Q10. Int J Mol Sci. 2019;20(20). 10.3390/ijms20205195 PMC6834161 31635164

[542] Zozina VI, Covantev S, Goroshko OA, Krasnykh LM, Kukes VG (2018). Coenzyme Q10 in cardiovascular and metabolic diseases: current state of the problem. Curr Cardiol Rev.

[543] Ayers J, Cook J, Koenig RA, Sisson EM, Dixon DL (2018). Recent developments in the role of coenzyme Q10 for coronary heart disease: a systematic review. Curr Atheroscler Rep.

[544] Serebruany VL, Gurbel PA, Ordonez JV, Herzog WR, Rohde M, Mortensen SA, Folkers K (1997). Could coenzyme Q10 affect hemostasis by inhibiting platelet vitronectin (CD51/CD61) receptor?. Mol Asp Med.

[545] Serebruany VL, Ordonez JV, Herzog WR, Rohde M, Mortensen SA, Folkers K, Gurbel PA (1997). Dietary coenzyme Q10 supplementation alters platelet size and inhibits human vitronectin (CD51/CD61) receptor expression. J Cardiovasc Pharmacol.

[546] Ya F, Xu XR, Shi Y, Gallant RC, Song F, Zuo X, Zhao Y, Tian Z, Zhang C, Xu X (2019). Coenzyme Q10 upregulates platelet cAMP/PKA pathway and attenuates integrin αIIbβ3 signaling and thrombus growth. Mol Nutr Food Res.

[547] Belardinelli R, Muçaj A, Lacalaprice F, Solenghi M, Seddaiu G, Principi F, Tiano L, Littarru GP (2006). Coenzyme Q10 and exercise training in chronic heart failure. Eur Heart J.

[548] Moccia M, Capacchione A, Lanzillo R, Carbone F, Micillo T, Perna F, De Rosa A, Carotenuto A, Albero R, Matarese G (2019). Coenzyme Q10 supplementation reduces peripheral oxidative stress and inflammation in interferon-beta1a-treated multiple sclerosis. Ther Adv Neurol Disord.

[549] Barden AE, Shinde S, Burke V, Puddey IB, Beilin LJ, Irish AB, Watts GF, Mori TA (2018). The effect of n-3 fatty acids and coenzyme Q10 supplementation on neutrophil leukotrienes, mediators of inflammation resolution and myeloperoxidase in chronic kidney disease. Prostaglandins Other Lipid Mediat.

[550] Fan L, Feng Y, Chen GC, Qin LQ, Fu CL, Chen LH (2017). Effects of coenzyme Q10 supplementation on inflammatory markers: a systematic review and meta-analysis of randomized controlled trials. Pharmacol Res.

[551] Sanoobar M, Eghtesadi S, Azimi A, Khalili M, Jazayeri S, Reza Gohari M (2013). Coenzyme Q10 supplementation reduces oxidative stress and increases antioxidant enzyme activity in patients with relapsing-remitting multiple sclerosis. Int J Neurosci.

[552] Sanoobar M, Eghtesadi S, Azimi A, Khalili M, Khodadadi B, Jazayeri S, Gohari MR, Aryaeian N (2015). Coenzyme Q10 supplementation ameliorates inflammatory markers in patients with multiple sclerosis: a double blind, placebo, controlled randomized clinical trial. Nutr Neurosci.

[553] Zhai J, Bo Y, Lu Y, Liu C, Zhang L (2017). Effects of coenzyme Q10 on markers of inflammation: a systematic review and meta-analysis. PLoS One.

[554] Tsai KL, Huang YH, Kao CL, Yang DM, Lee HC, Chou HY, Chen YC, Chiou GY, Chen LH, Yang YP (2012). A novel mechanism of coenzyme Q10 protects against human endothelial cells from oxidative stress-induced injury by modulating NO-related pathways. J Nutr Biochem.

[555] Forester BP, Harper DG, Georgakas J, Ravichandran C, Madurai N, Cohen BM (2015). Antidepressant effects of open label treatment with coenzyme Q10 in geriatric bipolar depression. J Clin Psychopharmacol.

[556] Mehrpooya M, Yasrebifar F, Haghighi M, Mohammadi Y, Jahangard L (2018). Evaluating the effect of coenzyme Q10 augmentation on treatment of bipolar depression: a double-blind controlled clinical trial. J Clin Psychopharmacol.

[557] Maguire A, Hargreaves A, Gill M. Coenzyme Q10 and neuropsychiatric and neurological disorders: relevance for schizophrenia. Nutr Neurosci. 2018;1–14. 10.1080/1028415X.2018.15564. 10.1080/1028415X.2018.1556481 30537908

[558] Maes M, Mihaylova I, Kubera M, Uytterhoeven M, Vrydags N, Bosmans E. Lower plasma Coenzyme Q10 in depression: a marker for treatment resistance and chronic fatigue in depression and a risk factor to cardiovascular disorder in that illness. Neuroendocrinol Lett. 2009;30(4):462–9. 20010493

[559] Bello RI, Kagan VE, Tyurin V, Navarro F, Alcain FJ, Villalba JM (2003). Regeneration of lipophilic antioxidants by NAD(P)H:quinone oxidoreductase 1. Protoplasma.

[560] Gomez-Diaz C, Rodriguez-Aguilera JC, Barroso MP, Villalba JM, Navarro F, Crane FL, Navas P (1997). Antioxidant ascorbate is stabilized by NADH-coenzyme Q10 reductase in the plasma membrane. J Bioenerg Biomembr.

[561] Isobe C, Abe T, Terayama Y (2009). Increase in the oxidized/total coenzyme Q-10 ratio in the cerebrospinal fluid of Alzheimer’s disease patients. Dement Geriatr Cogn Disord.

[562] Morris G, Puri BK, Walker AJ, Maes M, Carvalho AF, Walder K, Mazza C, Berk M (2019). Myalgic encephalomyelitis/chronic fatigue syndrome: from pathophysiological insights to novel therapeutic opportunities. Pharmacol Res.

[563] Yubero D, Allen G, Artuch R, Montero R (2017). The value of coenzyme Q(10) determination in mitochondrial patients. J Clin Med.

[564] Hernandez-Camacho JD, Bernier M, Lopez-Lluch G, Navas P (2018). Coenzyme Q10 supplementation in aging and disease. Front Physiol.

[565] McGarry A, McDermott M, Kieburtz K, de Blieck EA, Beal F, Marder K, Ross C, Shoulson I, Gilbert P, Mallonee WM (2017). A randomized, double-blind, placebo-controlled trial of coenzyme Q10 in Huntington disease. Neurology.

[566] LFd RI, Burón MI, Clarke CF, Villalba JM (2018). Polyunsaturated fatty acids directly regulate coenzyme Q biosynthesis. FASEB J.

[567] Beharry KD, Cai CL, Henry MM, Chowdhury S, Valencia GB, Aranda JV. Co-enzyme Q10 and n-3 polyunsaturated fatty acid supplementation reverse intermittent hypoxia-induced growth restriction and improved antioxidant profiles in neonatal rats. Antioxidants (Basel). 2017;6(4). 10.3390/antiox6040103 PMC5745513 29258174

[568] Barter P, Ginsberg HN (2008). Effectiveness of combined statin plus omega-3 fatty acid therapy for mixed dyslipidemia. Am J Cardiol.

[569] Choi HD, Chae SM (2018). Comparison of efficacy and safety of combination therapy with statins and omega-3 fatty acids versus statin monotherapy in patients with dyslipidemia: a systematic review and meta-analysis. Medicine (Baltimore).

[570] Toth S, Sajty M, Pekarova T, Mughees A, Stefanic P, Katz M, Spisakova K, Pella J, Pella D (2017). Addition of omega-3 fatty acid and coenzyme Q10 to statin therapy in patients with combined dyslipidemia. J Basic Clin Physiol Pharmacol.

[571] Zehr KR, Walker MK (2018). Omega-3 polyunsaturated fatty acids improve endothelial function in humans at risk for atherosclerosis: a review. Prostaglandins Other Lipid Mediators.

[572] Wiest EF, Walsh-Wilcox MT, Walker MK (2017). Omega-3 polyunsaturated fatty acids protect against cigarette smoke-induced oxidative stress and vascular dysfunction. Toxicol Sci.

[573] Hu Y, Hu FB, Manson JE (2019). Marine omega-3 supplementation and cardiovascular disease: an updated meta-analysis of 13 randomized controlled trials involving 127 477 participants. J Am Heart Assoc.

[574] Cohen MG, Rossi JS, Garbarino J, Bowling R, Motsinger-Reif AA, Schuler C, Dupont AG, Gabriel D (2011). Insights into the inhibition of platelet activation by omega-3 polyunsaturated fatty acids: beyond aspirin and clopidogrel. Thromb Res.

[575] McEwen BJ, Morel-Kopp MC, Chen W, Tofler GH, Ward CM (2013). Effects of omega-3 polyunsaturated fatty acids on platelet function in healthy subjects and subjects with cardiovascular disease. Semin Thromb Hemost.

[576] Adili R, Hawley M, Holinstat M (2018). Regulation of platelet function and thrombosis by omega-3 and omega-6 polyunsaturated fatty acids. Prostaglandins Other Lipid Mediators.

[577] Prossomariti A, Scaioli E, Piazzi G, Fazio C, Bellanova M, Biagi E, Candela M, Brigidi P, Consolandi C, Balbi T (2017). Short-term treatment with eicosapentaenoic acid improves inflammation and affects colonic differentiation markers and microbiota in patients with ulcerative colitis. Sci Rep.

[578] Andersen VL, Vogt J, Obel T, Christensen JH, Schmidt EB (2010). The effect of N-3 fatty acids on plasma myeloperoxidase levels in healthy adults. Cell Mol Biology (Noisy-le-Grand).

[579] Tan A, Sullenbarger B, Prakash R, McDaniel JC (2018). Supplementation with eicosapentaenoic acid and docosahexaenoic acid reduces high levels of circulating proinflammatory cytokines in aging adults: a randomized, controlled study. Prostaglandins Leukot Essent Fat Acids.

[580] Becic T, Studenik C (2018). Effects of Omega-3 supplementation on adipocytokines in prediabetes and type 2 diabetes mellitus: systematic review and meta-analysis of randomized controlled trials. Diabetes Metab J.

[581] O’Mahoney LL, Matu J, Price OJ, Birch KM, Ajjan RA, Farrar D, Tapp R, West DJ, Deighton K, Campbell MD (2018). Omega-3 polyunsaturated fatty acids favourably modulate cardiometabolic biomarkers in type 2 diabetes: a meta-analysis and meta-regression of randomized controlled trials. Cardiovasc Diabetol.

[582] Meital LT, Windsor MT, Perissiou M, Schulze K, Magee R, Kuballa A, Golledge J, Bailey TG, Askew CD, Russell FD (2019). Omega-3 fatty acids decrease oxidative stress and inflammation in macrophages from patients with small abdominal aortic aneurysm. Sci Rep.

[583] Mazereeuw G, Herrmann N, Andreazza AC, Scola G, Ma DWL, Oh PI, Lanctôt KL (2017). Oxidative stress predicts depressive symptom changes with omega-3 fatty acid treatment in coronary artery disease patients. Brain Behav Immun.

[584] Mas E, Woodman RJ, Burke V, Puddey IB, Beilin LJ, Durand T, Mori TA (2010). The omega-3 fatty acids EPA and DHA decrease plasma F2-isoprostanes: results from two placebo-controlled interventions. Free Radic Res.

[585] Herbst EAF, Paglialunga S, Gerling C, Whitfield J, Mukai K, Chabowski A, Heigenhauser GJF, Spriet LL, Holloway GP (2014). Omega-3 supplementation alters mitochondrial membrane composition and respiration kinetics in human skeletal muscle. J Physiol.

[586] Gerling CJ, Mukai K, Chabowski A, Heigenhauser GJF, Holloway GP, Spriet LL, Jannas-Vela S: Incorporation of omega-3 fatty acids into human skeletal muscle sarcolemmal and mitochondrial membranes following 12 weeks of fish oil supplementation. Front Physiol 2019, 10(348). 10.3389/fphys.2019.00348 PMC6449797 30984028

[587] Koga N, Ogura J, Yoshida F, Hattori K, Hori H, Aizawa E, Ishida I, Kunugi H (2019). Altered polyunsaturated fatty acid levels in relation to proinflammatory cytokines, fatty acid desaturase genotype, and diet in bipolar disorder. Transl Psychiatry.

[588] Solberg DK, Refsum H, Andreassen OA, Bentsen H (2019). A five-year follow-up study of antioxidants, oxidative stress and polyunsaturated fatty acids in schizophrenia. Acta Neuropsychiatrica.

[589] Messamore E, Almeida DM, Jandacek RJ, McNamara RK (2017). Polyunsaturated fatty acids and recurrent mood disorders: phenomenology, mechanisms, and clinical application. Prog Lipid Res.

[590] Morris G, Walder K, Puri BK, Berk M, Maes M (2016). The deleterious effects of oxidative and nitrosative stress on palmitoylation, membrane lipid rafts and lipid-based cellular signalling: new drug targets in neuroimmune disorders. Mol Neurobiol.

[591] Reimers A, Ljung H (2019). The emerging role of omega-3 fatty acids as a therapeutic option in neuropsychiatric disorders. Ther Adv Psychopharmacol.

